# Discovery of Novel *Bmy1* Alleles Increasing β-Amylase Activity in Chinese Landraces and Tibetan Wild Barley for Improvement of Malting Quality via MAS

**DOI:** 10.1371/journal.pone.0072875

**Published:** 2013-09-03

**Authors:** Xue Gong, Sharon Westcott, Xiao-Qi Zhang, Guijun Yan, Reg Lance, Guoping Zhang, Dongfa Sun, Chengdao Li

**Affiliations:** 1 College of Plant Science and Technology, Huazhong Agricultural University, Wuhan City, China; 2 Department of Agriculture and Food Western Australia, Perth City, Australia; 3 Western Australia State Agricultural Biotechnology Centre, Murdoch University, Perth City, Australia; 4 School of Plant Biology, Faculty of Natural and Agricultural Sciences, The University of Western Australia, Perth City, Australia; 5 Faculty of Agriculture, Life and Environmental Sciences, Zhejiang University, Hangzhou City, China; Cankiri Karatekin University, Turkey

## Abstract

China has a large barley germplasm collection which has not been well characterized and is therefore underutilized. The *Bmy1* locus encoding the *β*-amylase enzyme on chromosome 4H has been well characterized in the worldwide barley germplasm collections due to its importance in the malting and brewing industry. The *Bmy1* locus was chosen as an indicator to understand genetic potential for improvement of malting quality in Chinese landraces and Tibetan wild barley. The genetic diversity of 91 barley accessions was assessed using allele specific Multiplex-ready molecular markers. Eight accessions were further sequenced, based on the Multiplex-ready marker diversity for *Bmy1* in the germplasm. Six of the eight accessions clustered together in a unique group, and showed similarities to ‘Haruna Nijo’, wild barley accession PI296896 and ‘Ashqelon’. Sequence comparisons with the known *Bmy1* alleles identified not only the existing 13 amino acid substitutions, but also a new substitution positioned at A387T from a Chinese landrace W127, which has the highest β-amylase activity. Two new alleles/haplotypes namely *Bmy1-Sd1c* and *Bmy1-Sd5* were designated based on different amino acid combinations. We identified new amino acid combination of C115, D165, V233, S347 and V430 in the germplasm. The broad variation in both *β*-amylase activity and amino acid composition provides novel alleles for the improvement of malting quality for different brewing styles, which indicates the high potential value of the Chinese landraces and Tibetan wild barley.

## Introduction

A significant high value use of barley is for malting to produce malt as a raw material for brewing beer and producing whiskey. Based on the European Brewing Convention, malting quality is determined by more than 30 traits/parameters [1]. A major process of malting and brewing is to convert starch into fermentable sugars. Four major enzymes are involved in this process, including α-amylase (α-1,4-glucan glucanohydrolase; EC 3.2.1.1), β-amylase (α-1,4-glucan maltohydrolase; EC 3.2.1.2), limit dextrinase (*α*-dextrin 6-glucanohydrolase; EC 3.2.1.142) and α-glucosidase (α -D-Glucoside glucohydrolase; EC 3.2.1.20) [2]. The combined contribution of the amylotic enzymes is known as diastatic power (DP). Depending on mashing and brewing processes, the requirement for DP can vary from low to moderate to very high. For example, starch adjuncts are used to brew beer in Asia and North America, thus a high to very high DP is required to degrade the extra starch. Alternatively, when full malt or liquid sugars are used to produce beers in Europe and Australia, lower or moderate levels of DP are preferred. New genetic variation for malting quality has always been a target for brewers and breeders.

Among malting quality parameters, beta-amylase is the most thoroughly studied and explored due to its importance for DP. The enzyme is encoded by *Bmy1* locus at the telomeric region of barley chromosome 4H [3]. The genetic diversity of *Bmy1* alleles and functions of the insertion/deletions (INDELs), single nucleotide polymorphisms (SNPs) in the *Bmy1* genomic and amino acid substitutions in the protein sequences are all well characterized in barley collections in Japan [4], Europe [5], North America [6], [7] and Australia [8]. The genetic structure of the *Bmy1* allele dictates the β-amylase isoenzyme type, activity, thermostability and enzyme/inhibitor ratio [3]. The genomic DNA sequence of *Bmy1* has a 5 kb segment, comprising 3.7 kb of coding region and 1.5 kb of DNA sequence upstream of the transcription initiation site. This gene contains seven exons and six introns, and the full-length cDNA sequence is encoded for a polypeptide of 535 amino acids. So far, seven haplotypes of *Bmy1* are known as *Bmy1-Sd1a*, *Bmy1-Sd1b*, *Bmy1-Sd2L*, *Bmy1-Sd2H*, *Bmy1-Sd2Ha*, *Bmy1-Sd3* and *Bmy1-Sd4* [5], [9]. The formation of these haplotypes appears to correspond with growth habit (spring or winter), row type (two or six row) and geographic region of the germplasm. In the germplasm collected from Asia and the Middle East, most of the haplotypes carry *Bmy1-Sd2H* with some signatures of *Bmy1-Sd3* and *Bmy1-Sd1b*. In contrast, the *Bmy1-Sd4* haplotype predominates in European germplasm [5], [9]. Spring barley varieties are more likely to have *Bmy1-Sd1a*, *Bmy1-Sd2H* and *Bmy1-Sd2L* haplotypes, while winter varieties are more likely to have the *Bmy1-Sd4* haplotype. Maylesheva-Otto and Roder [5] suggested that the SNP markers at amino acid positions 115, 233 and 347 are sufficient to discriminate most of the seven haplotypes.

China has the world's largest barley germplasm collection. The Qinghai-Tibetan plateau has been suggested as the centre of origin for Chinese landraces [10]. However, to date, the knowledge of the Chinese barley germplasm gene pool has been restricted to differences between Chinese landraces and Tibetan wild barley in some traits, such as salt tolerance [11] and protein content [12]. There is no comprehensive research on the genetic variation of malting quality traits [13]. In this study, we selected the *Bmy1* locus as an indicator to understand the genetic potential for improvement of malting quality using 91 accessions of Chinese landraces and Tibetan wild barley. Our results demonstrated that the Chinese barley germplasm is an underutilized but potentially highly valuable resource of the global barley gene pool.

## Materials and Methods

### Plant materials

**Figure 1:**
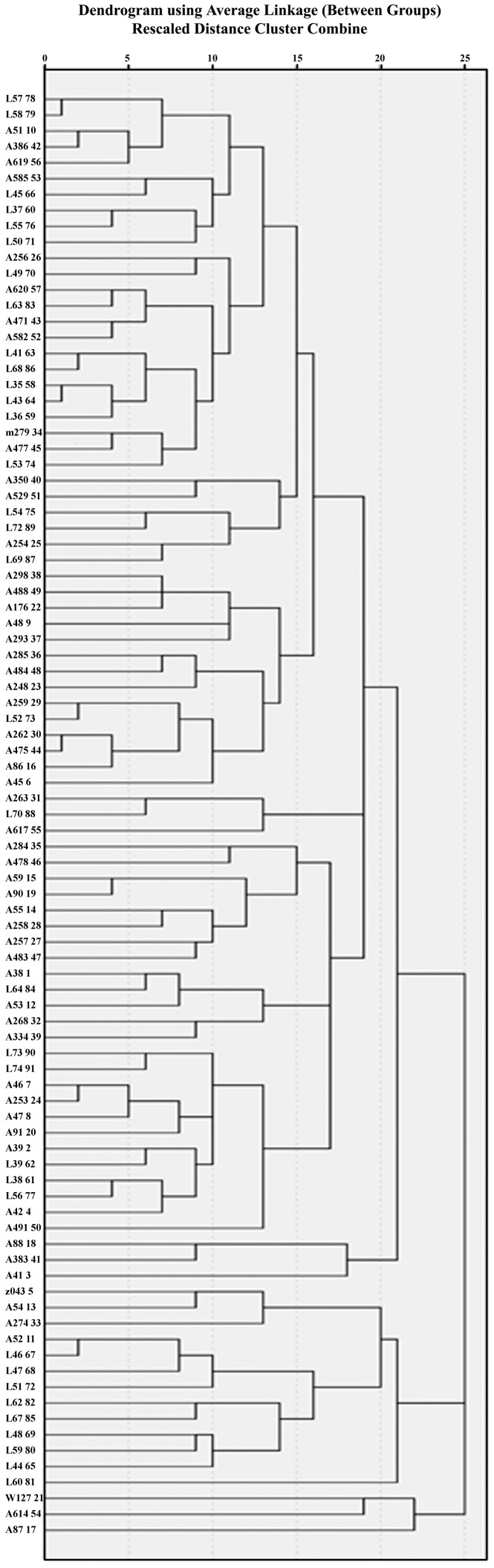
Genetic cluster of 91 accessions by Multiplex-ready SSR markers. The numbers after the accession numbers were the system number given by SPSS software.

Ninety-one barley accessions were initially selected from a small barley germplasm collection in Huazhong Agricultural University, including 34 Tibetan wild barley and 57 Chinese landraces (Figure 1, Table S1). These selected lines were grown in the experimental farm of Huajiachi campus, located in Hangzhou, China. The experiment was a complete block design with two replicates. An Israeli wild barley accession L46 (HUA032) was selected as a control. Based on molecular diversity study, eight barley accessions were selected for sequencing. These entries are z043 (zaofengyihao), W127 (W84-127), m279 (maerkangsileng) and wild *Hordeum vulgare* subsp. *spontaneum* accessions L35 (HUA01), L47 (HUA033), L48 (HUA052) and L68 (HUA0640).

### DNA extraction and diversity study

DNA extraction was conducted following the same method as Gong et al. [10]. Twelve pairs of Multiplex-ready primers hv1006, hv1010, hv1013, hv1014, hv1015, hv1016, hv1018, hv1019, hv1020, hv1021, hv1022, and hv1023 were employed to screen the 91 accessions (Table S2). These primers were designed based on INDELs to discriminate *Bmy1* haplotypes in the germplasm. Application of the Multiplex-ready primers followed the protocol by Hayden et al. [14]. The presence of a band was scored as 1 and the absence as 0. Data collected from the Multiplex-ready Polymerase Chain Reaction (PCR) screening were analyzed by SPSS software to construct the relationship between accessions (Figure 1).

### PCR purification and cloning

Twelve paired primers (Table S3) were designed to amplify *Bmy1* genomic sequences. The primers were synthesized by GeneWorks (Sydney, New South Wales). PCR was carried out in a final volume of 10 µL, containing 1 µL of the 25 ng µL^–1^ genomic DNA, 1.0 µL of 10 ×PCR buffer, 0.3 µL of 25 mM Mg^2+^, 0.4 µL of 10 mM dNTP mixture, 3 µL of cresol red, 1 µL of 10% pvp (polyvinylpyrrolidone), 1 µL of a 10 pmole solution of the forward and reverse primers, and 0.1 µL of Taq DNA polymerase (Invitrogen, Texas, America). PCR conditions for these primers were optimized to get the best result (Table S3). Two PCR programs were used for PCR amplification depending on the Tm. The first program involved one cycle of 94°C for 1 min, 61°C for 45 s, and 72°C for 1 min, 5 cycles from 60°C to 55°C with 1°C decrease by each cycle, followed by 25 cycles of 94°C for 1 min, 55°C for 30 s, and 72°C for 1 min, with a final extension of 72°C for 5 min. The second program consisted of five cycles of 94°C for 1 min, 62 for 45 s, and 72°C for 1 min, 20 cycles of 94°C for 1 min, 58°C for 45 s, and 72°C for 1 min, followed by 25 cycles of 94°C for 1 min, 55°C for 30 s, and 72°C for 1 min with a final extension at 72°C for 5 min. The two PCR programs were primer specific, e.g. for the same primer pair, all eight varieties used the same program. PCR products were resolved on 2% agarose gels and stained with ethidium bromide. Single band amplicons were later purified by QIAquick PCR purification kit (Qiagen). A final volume of 25 µl purified PCR product was prepared for plasmid cloning. Ligation kit (Promega) was used to ligate the target band into a pGEM-T easy vector. Two clones were selected for each individual fragment. DNA amplicon was sequenced in both directions for each cloned fragment of the *Bmy 1* gene, and an ABI-automated DNA sequencer (Applied Biosystems) was used to obtain the sequences from each primer pair. Template preparation and sequencing reactions were prepared according to the user's manual. Repeated experiments were conducted on any new nucleotide base identified in this study to elucidate any chance of experimental errors.

### Genomic DNA sequence assembly and alignment

*Bmy1* sequencing and sequence alignment were achieved by using Bioedit software [15]. The assembly and alignment process for one full sequence was as follows: **Step 1**, both 5′ to 3′ and 3’ to 5′ DNA strands of each cloned fragment were sequenced along its entire length, in order to eradicate the errors during reading and/or editing. The quality of each sequence was blasted online using NCBI BLAST (http://blast.ncbi.nlm.nih.gov). By this method 12 clean, 5′ to 3′ sequences amplified by each pair of primers were analyzed for each accession. **Step 2,** a Phred quality score of ≥25 was used to assemble allele contigs for each variety. **Step 3**, 8 full *Bmy1* sequences together with reference *Bmy1* sequences were aligned together by ClustalW analysis using default parameters. This step determined the similarities and differences among the eight selected accessions. Also, a genetic tree using the ‘DNA – > Neighbor phylogenetic tree’ method at *Bmy1* full sequence level was produced. **Step 4**, the full *Bmy1* sequence of the eight varieties was aligned individually with cDNA sequence of ‘Haruna Nijo’ to determine the cDNA sequence, as ‘Haruna Nijo’ has the beta-amylase with high enzyme activity and thermostability [8], [17]. The cDNAs of eight accessions together with reference *Bmy1* sequences were aligned together by ClustalW analysis using a default parameter to identify the SNP differences. **Step 5**, the eight cDNA sequences obtained from **Step 4** were converted into amino acid sequences using the ‘Translate Frame 1’ method.

#### Bmy1 genomic and amino acid sequence of sample accessions

Sequences of mRNA and full genomic *Bmy1* were submitted to and assigned by GeneBank for the following accession numbers: z043 (KF302660, KF302668), W127 (KF302661, KF302669), m279 (KF302662, KF302670), L35 (KF302663, KF302671), L46 (KF302664, KF302672), L47 (KF302665, KF302673), L48 (KF302666, KF302674) and L68 (KF302667, KF302675). Nineteen accessions representing the documented *Bmy1* SNPs and haplotypes were retrieved from GeneBank: Morex (EU589328), Harrington (FJ161079), HA52 (AJ301645), Strider (EU589327), Haruna Nijo (D21349), PI296897 (AF061204), AB75 (TrEMBL P82993), Adorra (AF061203), Hiproly (X52321), Steptoe (EF175470), Orca (EF175468), Legacy (FJ161080), Stander (EF175469), Tango (EF175471), UC958 (EF175472), UC960 (EF175473), Schooner (AF300799), Franklin (AF300800) and Ashqelon (FJ161078).

### Assay of β-amylase activity and statistic tests

*β*-amylase activity was assayed by using protocols in literature [17], but modified accordingly to the instructions of Betamyl assay kit (Megazyme International, Ireland Ltd). Each measurement was conducted in triplicate. The final *β*-amylase activity was defined as U g^–1^. Duncan's multiple range tests in GenStat (Version 14) was conducted to compare the differences of enzyme activity among samples.

## Results

### Screening for *Bmy1* diversity by INDELs

The Multiplex-ready PCR markers developed from INDELs divided the 91 accessions into two large groups. Group 1 had 16 accessions and Group 2 had 75 accessions. In Group 1, the two-rowed wild barleys clustered together, and the six-rowed landraces clustered together (Table S1, Figure 1). The distribution of the accessions in Group 2 did not show any specific pattern as wild barleys scattered among the landraces. Numerous small groups were also observed in each large group. Based on the classification of INDELs in *Bmy1* intronic and promoter sequences, as well as a previous genetic diversity study using the same materials [10], eight accessions were further selected to analyse enzyme activity and to sequence the full *Bmy1* sequence.

### *Bmy1* cDNA and amino acid polymorphisms

Twenty-one SNPs were identified in the cDNA sequences, resulting in 13 amino acid substitutions (Table 1). The amino acid T387A substitution was specific in the Chinese landrace W127, which was caused by an 1159 ^T→A^ substitution in the cDNA sequence. In this study, amino acid substitutions A453T (1357 ^A→T^), V488I (1462 ^G→A^) and G518R (1552 ^G→A^) appeared in Tibetan wild barley L48, Israeli wild barley L46 and North American barley Strider. Meanwhile, the M527I (1581 ^T→A^) amino acid substitution was identified in m279. The 1425 ^G→A^ substitution in L48 and L46 caused no amino acid substitution in the protein sequence (Table 1, Table S4).

**Table 1:** Single Nucleotide polymorphisms for the eight barley accessions compared with 18 accessions representing the *Bmy1* haplotypes.

Haplotypes	Genotypes	a185	343	402	471	495	531	591	666	698	702	736	741	868	945	1040	1159	1289	1293	1357	1414	1425	1462	1552	1581
		bP62L	R115C	T134	T157	D165E	V177	S197	V222	V233A	G234	F246L	R247	L290	Y315	L347S	T387A	V430S	N431	A453T	Q472K	L475	V488I	G518R	M527I
*Bmy1-Sd1a*	Morex	cC (P)	T (C)	T	A	G (E)	A	G	T	T (V)	G	T (F)	A	C	C	C (S)	A (T)	C (A)	T	G (A)	C (Q)	G	G (V)	G (G)	T (M)
	Franklin	C (P)	T (C)	T	A	G (E)	A	C	T	T (V)	G	T (F)	A	C	C	C (S)	A (T)	C (A)	T	G (A)	C (Q)	G	G (V)	G (G)	T (M)
	Harrington	C (P)	T (C)	T	A	G (E)	A	G	T	T (V)	G	T (F)	A	C	C	C (S)	A (T)	C (A)	T	G (A)	C (Q)	G	G (V)	G (G)	T (M)
	HA52	C (P)	T (C)	T	A	G (E)	A	G	T	T (V)	G	C (L)	A	C	C	C (S)	A (T)	C (A)	T	G (A)	C (Q)	G	G (V)	G (G)	T (M)
	z043	C (P)	T (C)	T	A	G (E)	A	G	T	T (V)	G	T (F)	A	C	C	C (S)	A (T)	C (A)	T	G (A)	C (Q)	G	G (V)	G (G)	T (M)
	L47	C (P)	T (C)	T	A	G (E)	A	G	T	T (V)	G	T (F)	A	C	C	C (S)	A (T)	C (A)	T	G (A)	C (Q)	G	G (V)	G (G)	T (M)
*Bmy1-Sd1b*	Strider	C (P)	T (C)	T	A	G (E)	G	G	T	T (V)	A	T (F)	A	C	C	C (S)	A (T)	T (V)	T	A (T)	C (Q)	A	A (I)	A (R)	T (M)
*Bmy1-Sd1c*	L46	C (P)	T (C)	C	A	G (E)	G	G	C	C (A)	A	T (F)	G	C	C	C (S)	A (T)	T (V)	T	A (T)	C (Q)	A	A (I)	A (R)	T (M)
*Bmy1-Sd2L*	Hiproly	C (P)	C (R)	T	A	C (D)	G	G	T	T (V)	A	T (F)	A	C	C	T (L)	A (T)	T (V)	T	G (A)	C (Q)	G	G (V)	G (G)	A (I)
	Adorra	C (P)	C (R)	T	A	C (D)	G	G	T	T (V)	A	T (F)	A	C	C	T (L)	A (T)	T (V)	T	G (A)	C (Q)	G	G (V)	G (G)	T (M)
	Schooner	C (P)	C (R)	T	C	C (D)	G	G	T	T (V)	A	T (F)	A	C	C	T (L)	A (T)	T (V)	T	G (A)	C (Q)	G	G (V)	G (G)	A (I)
	m279	C (P)	C (R)	T	A	C (D)	G	G	T	T (V)	A	T (F)	A	C	C	T (L)	A (T)	T (V)	T	G (A)	C (Q)	G	G (V)	G (G)	A (I)
*Bmy1-Sd2H*	Haruna Nijo	C (P)	C (R)	T	A	C (D)	A	G	C	C (A)	A	T (F)	G	T	T	C (S)	A (T)	T (V)	C	G (A)	C (Q)	G	G (V)	G (G)	T (M)
	L35	C (P)	C (R)	T	A	C (D)	G	G	C	C (A)	A	T (F)	G	C	C	C (S)	A (T)	T (V)	T	G (A)	C (Q)	G	G (V)	G (G)	T (M)
	L48	C (P)	C (R)	T	A	C (D)	G	G	C	C (A)	A	T (F)	G	C	C	C (S)	A (T)	T (V)	T	G (A)	C (Q)	A	A (I)	A (R)	T (M)
	L68	C (P)	C (R)	T	A	C (D)	G	G	C	C (A)	A	C (L)	G	C	C	C (S)	A (T)	T (V)	T	G (A)	C (Q)	G	G (V)	G (G)	T (M)
*Bmy1-Sd2Ha*	PI296897	C (P)	C (R)	T	A	C (D)	G	G	C	C (A)	A	T (F)	G	C	C	C (S)	A (T)	T (V)	T	G (A)	C (Q)	G	G (V)	G (G)	T (M)
*Bmy1-Sd3*	AB75	T (L)	C (R)	T	A	G (E)	G	G	T	C (A)	A	T (F)	G	T	T	C (S)	A (T)	T (V)	C	G (A)	A (K)	G	G (V)	G (G)	T (M)
*Bmy1-Sd4*	Stander	C (P)	C (R)	T	A	C (D)	G	G	T	T (V)	A	T (F)	A	C	C	C (S)	A (T)	T (V)	T	G (A)	C (Q)	G	G (V)	G (G)	T (M)
	Legacy	C (P)	C (R)	T	A	C (D)	G	G	T	T (V)	A	T (F)	A	C	C	C (S)	A (T)	T (V)	T	G (A)	C (Q)	G	G (V)	G (G)	T (M)
	Orca	C (P)	C (R)	T	A	C (D)	G	G	T	T (V)	A	T (F)	A	C	C	C (S)	A (T)	T (V)	T	G (A)	C (Q)	G	G (V)	G (G)	T (M)
	Tango	C (P)	C (R)	T	A	C (D)	G	G	T	T (V)	A	T (F)	A	C	C	C (S)	A (T)	T (V)	T	G (A)	C (Q)	G	G (V)	G (G)	T (M)
	UC958	C (P)	C (R)	T	A	C (D)	G	G	T	T (V)	A	T (F)	A	T	T	C (S)	A (T)	T (V)	T	G (A)	C (Q)	G	G (V)	G (G)	T (M)
	UC960	C (P)	C (R)	T	A	C (D)	G	G	T	T (V)	A	T (F)	A	C	C	C (S)	A (T)	T (V)	T	G (A)	C (Q)	G	G (V)	G (G)	T (M)
	Steptoe	C (P)	C (R)	T	A	C (D)	G	G	T	T (V)	A	T (F)	A	C	C	C (S)	A (T)	T (V)	T	G (A)	C (Q)	G	G (V)	G (G)	T (M)
	Ashqelon	C (P)	T (C)	C	A	C (D)	G	G	C	C (A)	A	T (F)	G	C	C	C (S)	A (T)	T (V)	T	G (A)	C (Q)	G	G (V)	G (G)	T (M)
	W127	C (P)	T (C)	T	A	C (D)	G	G	C	C (A)	A	T (F)	A	C	C	C (S)	***G*** * (* ***A*** *)*	T (V)	T	G (A)	C (Q)	G	G (V)	G (G)	T (M)

a The numbers in this row representing the nucleotide acid position in the cDNA sequences of *Bmy1* alleles.

b Capital letters in this row and brackets in the table representing the amino acid as following: P: Proline; L: Leucine; R: Arginine; C: Cysteine; T: Threoline; D: Aspartic; E: Glutamic; V: Valine; S: Serine; Q: Glutamine; K: Lysine; A: Alanine; G: Glycine; F: Phenylalanine; Y: Tyrosine; N: Asparagine; I: Isoleucine; M: Methionine. The numbers in this row are the amino acid positions in the protein sequence. The SNPs caused amino acid substitutions were in bold.

c The “A”, “C”, “T” and “G” represent for the nucleotide composition in the genotypes at each cDNA position of cDNA sequences. The capital letters in the brackets were amino acid referring to “^c^”. The unique substitution was in bold italic.

The Chinese landrace z043 and Tibetan wild barley L47 had the same amino acid compositions as Harrington, HA52 and Franklin. The Chinese landrace barley m279 had the same amino acid composition as Hiproly and the Tibetan wild barley L35 had the same amino acid composition as Haruna Nijo. Although the Israeli wild barley L46 had an amino acid composition similar to Tibetan wild barley L48 and North American barley Strider, its amino acid composition was unique. L46 differed from L48 at amino acid 115 and 165, and the amino acid sequence of L46 differed to Strider at 233. The composition of amino acid substitutions of Tibetan wild barley L48 differed from Strider at 233. The amino acid of Tibetan wild barley L68 differentiated from wild barley PI 296897 at 246. The Chinese landrace W127 had an amino acid composition which differed from Ashqelon at 387.

### *Bmy1* intron III polymorphisms

INDELs of 126 bp, 38 bp, 11 bp, 4 bp, 21 bp and 6 bp in intron III were observed in the present study (Table 2, Table S4). The selected eight barley accessions had six types of INDELs in intron III. The Chinese landraces m279 and z043 had the same deletion pattern as Adorra and Harrington, respectively. A third Chinese landrace W127 and Israeli wild barley L46 had the same INDEL pattern as wild barley Ashqelon. Tibetan wild barleys L35 and L68 had the same intron III INDELs as Haruna Nijo. The last two intron III INDEL types of Tibetan wild barleys L47 and L48 differed from the remaining six barley accessions and references. The intron III of L47 distinguished wild barleys PI296897 and AB75 with the presence of 6 bp INDELs, and the intron III of L48 could be distinguished from Haruna Nijo with the presence of 6 bp INDELs. The intron III of L47 and L48 differed by a 38 bp INDEL and L47 had the deletion (Table 2). It is interesting to note that a 126 bp insertion was observed in Adorra, m279 and other varieties from Europe; whereas a 38 bp deletion was observed in wild barleys like PI296897, L47 and AB75.

**Table 2:** *Bmy1* intro III polymorphisms for eight barley genotypes in this study compared to 18 *Bmy1* genotypes.

aIntron III allele	bBmy1haplotypes	cGenotypes	Deletion (bp)					
			d2634126 bp	283938 bp	320911 bp	33064 bp	333721 bp	36456 bp
*Bmy1.a*	*Bmy1-Sd2L*	Adorra	+	+	−	+	+	+
		m279	+	+	−	+	+	+
	*Bmy1-Sd4*	Legacy	+	+	−	+	+	+
		Orca	+	+	−	+	+	+
		Steptoe	+	+	−	+	+	+
		Tango	+	+	−	+	+	+
		UC958	+	+	−	+	+	+
		UC960	+	+	−	+	+	+
		Stander	+	+	−	+	+	+
*Bmy1.b*	*Bmy1-Sd1a*	Morex	−	+	−	+	−	+
		Harrington	−	+	−	+	−	+
		HA52	−	+	−	+	−	+
		z043	−	+	−	+	−	+
	*Bmy1-Sd1b*	Strider	−	+	−	+	−	+
*Bmy1.c*	***Bmy1-Sd1c***	L46	−	+	+	−	+	−
	*Bmy1-Sd2H*	Haruna Nijo	−	+	+	−	+	−
		L35	−	+	+	−	+	−
		L68	−	+	+	−	+	−
	*Bmy1-Sd2H*	L48	−	+	+	−	+	+
	***Bmy1-Sd5***	Ashqelon	−	+	+	−	+	−
		W127	−	+	+	−	+	−
*Bmy1.d*	*Bmy1-Sd2Ha*	PI296897	−	−	+	−	+	−
	*Bmy1-Sd3*	AB75	−	−	+	−	+	−
	*Bmy1-Sd1a*	L47	−	−	+	−	+	+

a Nomenclautre for β-amylase intron II promoted by reference [7].

b Nomenclature for β-amylase amino acid haplotypes promoted by reference [9], and new haplotypes identified in this study were in bold.

c Genotypes utilized in this study were underlined.

d Numbers in the row were the position of nucleotide base in the full *Bmy1* sequence in Additional file 1, “−” and “+” meant deletion and insertion of the sequence respectively.

### *Bmy1* genomic DNA polymorphisms

The 12 pairs of *Bmy1* primers identified an open reading frame interrupted by seven introns. Among the barley accessions, a full-length sequence is composed of 5200 bp with 43 deletions observed in the full sequence. A 92 bp deletion in the promoter region was identified in Tibetan wild barley L47, and a 126 bp deletion in intron III was identified in all barley accessions except the Chinese landrace m279. Compared with the reference *Bmy1* alleles, the eight barley accessions could be distinguished by the 22 SNPs and 2 INDELs in the *Bmy1* genomic sequence (Tables S5 and S6). One of the two INDELs was 11 bp in length and located at *Bmy1* genomic position 205, and the other was 4 bp in length and located at *Bmy1* genomic position 411. Both INDELs were in the promoter region of the *Bmy1* gene.

**Figure 2:**
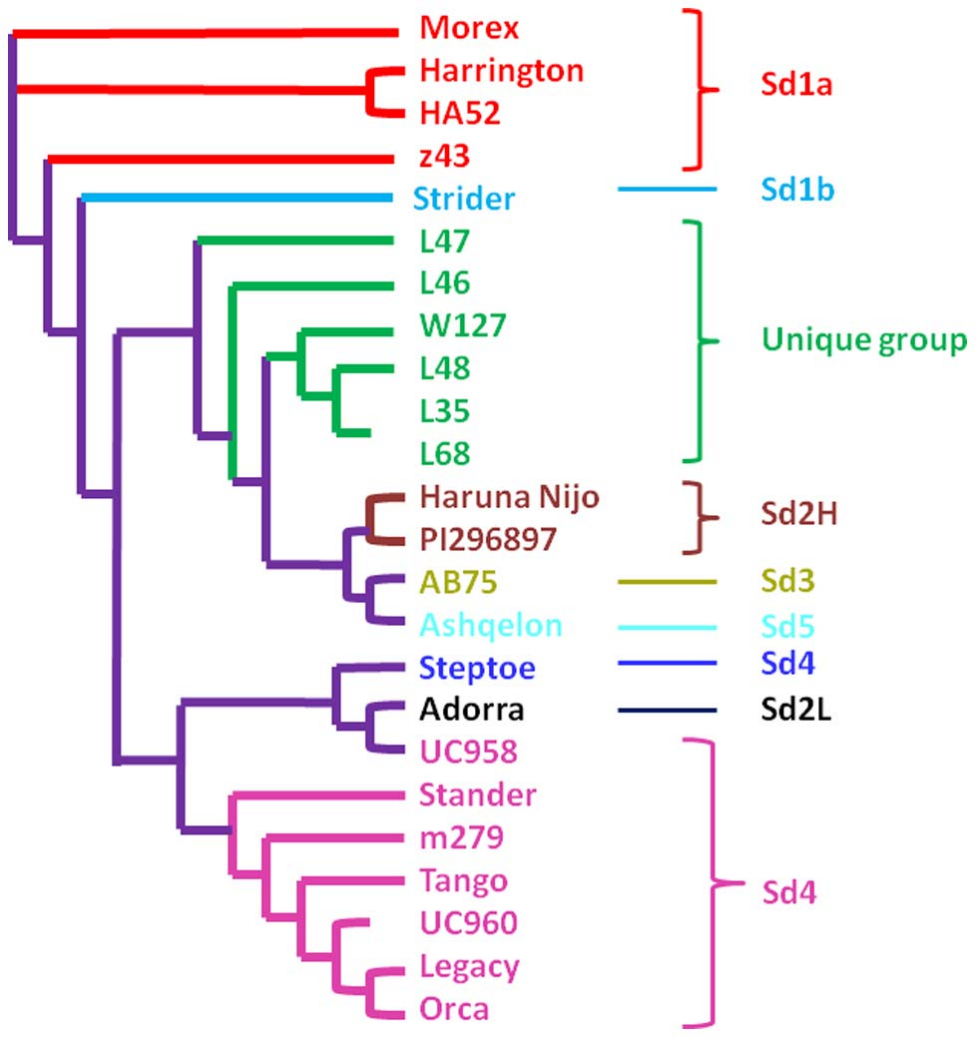
Neighbor-Joining genetic similarity of *Bmy1* alleles based on full length *Bmy1* sequences.

Neighbor-Joining genetic similarity analysis revealed that six of the eight selected accessions clustered into a unique group and showed similarity to Haruna Nijo (a Japanese malting barley variety), and wild barleys PI296897 and Ashqelon (Figure 2). The Chinese landrace z043 was clustered with the North American varieties Morex, Harrington, HA52 and Strider. The other Chinese landrace m279 was clustered with European cultivars Stander, Tango and Legacy.

### Phenotypic variation of β-amylase activity

A wide range of β-amylase activity was observed in the eight barley accessions:β-amylase activity from 8 varieties were classed into 6 ranks (Table 3). The highest and lowest β-amylase activities were observed in the landraces W127 and z043 respectively, with significant difference at P≤0.05. The β-amylase activity of Israeli barley L46 was higher than that of landrace z043 and lower than that of wild barley L68, but these differences were not significant. Wild barley L48 had a significantly higher β-amylase activity than that of remaining wild barley. However, its activity was significantly lower than that of landrace W127. Wild barley L35 and L47 had similar β-amylase activity.

**Table 3:** β-amylase activities of selected accessions to identify new amino acid substitutions in Chinese landraces and wild barley.

Number	Accession Name	Origin	Type	β-amylase activity	Coefficient of	*Duncan's Multiple
				Unit: U g^–1^	variation (%)	Range tests
z043	zaofengyihao	Sichuan	landraces	867.59	6.71	a
W127	W84 −127	Sichuan	landraces	1629.90	5.57	f
m279	maerkangsileng	Sichuan	landraces	1009.64	7.47	c
L35	HUA01	Tibet	wild	1090.64	6.67	d
L46	HUA032	Israel	wild	920.42	3.95	ab
L47	HUA033	Tibet	wild	1088.70	12.91	d
L48	HUA052	Tibet	wild	1278.88	3.59	e
L68	HUA0640	Tibet	wild	942.33	6.56	b

* small letters indicated ranks of differences between β-amylase activity in one variety and that of remaining seven varieties. No significant difference (P>0.05) was defined if varieties fell into the same rank (by sharing the same letter). Significant difference (P≤0.05) was defined if varieties fell into different ranks. Varieties fell into a rank of two letters were the intermediate rank which were not significantly different to either of the single rank.

## Discussion

### *Bmy1* gene structure and β-amylase activity

Sequencing the full-length *Bmy1* gene in eight barley accessions revealed wide diversity, where none of the accessions shared exactly the same *Bmy1* genomic sequence. One nucleotide substitution at cDNA 1159 ^T→A^ resulted in a T387A amino acid change in the Chinese landrace W127, a change not yet reported. The full sequence of *Bmy1* amino acid of W127 was compared with reference sequences, and it was most similar to wild barley Ashqelon. The sole difference in amino acid sequences of W127 and Ashqelon was at the new amino acid substitutions identified in W127 at 387. Except for the amino acid substitution at 387, the full *Bmy1* sequence of W127 also differed from Ashqelon at 14 bp deletion at the *Bmy1* genomic position 2022 (Table S4). Amino acid comparisons of the *Bmy1* gene in W127 and Ashqelon revealed that both accessions had a composition of C115, D165, V233, S347 and V430 which distinguished it from Haruna Nijo at 115. The 115^ R→C^ substitutions might be the reason for high β-amylase activity in W127 and Ashqelon, and for the differences in kinetic properties, isoelectric focusing pattern and the enzyme/inhibitor binding ratio [13], [18]. A mapping population of Ashqelon x W127 or near isogenic lines with the *Bmy1* gene from these two varieties may clarify the association of T387A with β-amylase activity. Measurement of β-amylase activity of both Ashqelon and W127 grown in same conditions can also provide a sound comparison between the *Bmy1* sequence and β-amylase activity. Thus, the amino acid composition of C115, D165, V233, S347 and V430 in W127 and Ashqelon can become useful resources for malting barley varieties.

The signature of 402^T → C^ substitution in cDNA was recognized in Israeli wild barley L46 and the 1425^G→A^ substitution was observed in both L46 and Tibetan wild barley L48. Neither a 402^T→C nor^ 1425^G→A^ substitution caused an amino acid change in the protein sequence. The 402^T→C^ and 1425^G→A^ substitutions were specific to wild barley Ashqelon and cultivar Strider, respectively. The *Bmy1* alleles of Ashqelon and Strider represent rare *Bmy1* haplotypes in the world gene pool of barley germplasm [6], [19], and each case was identified in the eight selected barley accessions, demonstrating the richness of the barley gene pool in our collection. Three other amino acid substitutions were also observed at A453T (1357 ^A→T^), V488I (1462 ^G→A^) and G518R (1552 ^G→A^) in Israeli wild barley L46 and Tibetan wild barley L48. For the amino acid compositions, L46 had C115, E165 and V233, and L48 had R115, D165 and A233. For the β-amylase activity, L46 had lower β-amylase activity than L48, indicating that an amino acid composition of C115, E165 and V233 would have higher β-amylase activity than R115, D165 and A233, which was confirmed by Chiapparino et al. [9]. By comparing the full amino acid sequence of L46, L48 and Strider, it was found that L46 differentiated from Strider only at A233, but at R115, D165 and A233 in L48. L46 had an A233 instead of a V233 in Strider, so the β-amylase activity in L46 was not as low as that in Strider, which had remarkably low DP and β-amylase activity [6]. This result supported the previous finding that a cultivar carrying an A233 would have higher β-amylase activity than a cultivar carrying a V233 [9]. To expand, this finding about an A233 or V233 helped to understand that having either an R or a C in position 115 had no effect on β-amylase activity; when a C115 appears, the combination of C115 and D165 decreased β-amylase activity and thermostability, and an A at position 233 becomes essential for higher β-amylase activity [18], [20]. A high polymorphic intron III was observed in the eight barley accessions, which, were classified into six intron III types, demonstrating the genetic diversity within the germplasm collection. A nomenclature of the *Bmy1* intron III haplotypes was proposed by the presence or absence of 126, 38, 11 and 21 bp INDELs as: *Bmy1.a*, presence of 126 bp, 38 bp, 21 bp and absence of 11bp; *Bmy1.b*, presence of 38 bp and absence of 126 bp, 21 bp, 11 bp; *Bmy1.c*, presence of 11 bp, 38 bp, 21 bp and absence of 126 bp; *Bmy1.d*, presence of 11 bp and 21 bp and absence of 126 bp and 38 bp [7]. By this nomenclature, Adorra, Legacy, Orca, Steptoe, Tango, UC958, UC960, Stander and Chinese landrace m279 possess the *Bmy1.a* allele. Morex, Harrington, HA52, Strider and Chinese landrace z043 contain the *Bmy1.b* allele. Haruna Nijo, wild barley Ashqelon, Tibetan wild barley L35, L48 and L68, as well as Israeli wild barley L46 possess the *Bmy1.c* allele. Wild barley PI296897, AB75 and Tibetan wild barley L47 share the *Bmy1.d* allele (Table 2). *Bmy1.a* intron III haplotype had the most insertions, which produced the longest intron III fragment. The Chinese landrace m279 was the only accession of the eight to carry the *Bmy1.a* intron III haplotype, which is the only intron III haplotypes possessing the 126 bp insertions. The longer fragment in the *Bmy1.a* intron III haplotype may serve as a binding site for a negative transcription factor, which caused low β-amylase activity [24]. *Hordeum spontaneum*, with high β-amylase activity, did not have the 126 bp, and the progenies of Adorra × *Hordeum spontaneum* PI296897 inherited the intron III of PI296897 by 3:1 [4]. Coventry et al. [25] found the presence of 126 bp INDELs was associated with lower DP and lower *β*-amylase activity. It has been speculated that the molecular mechanism by which the 126 bp INDELs sequence might influence qualitative or quantitative expression of *Bmy1* involves elements in the 126 bp sequence binding to a factor(s) that modulates *Bmy1* transcription efficiency or influences post-transcriptional events such as mRNA maturation and stability [4]. The *Bmy1.d* intron III allele seems to be unique to wild barleys. The nomenclature proposed by Vanjie et al. [7] excluded the 6 bp and 11 bp INDELs reported by Sjakste and Roder [26], because Vanjie et al. [7] claimed that the 6 bp insertion was in conjunction with the 11 bp deletion in intron III, and the use of 6 bp INDELs in assigning *Bmy1* intron III alleles was redundant. In this study, the presence of 6 bp indel and absence of 11 bp deletion was not in conjunction with the Tibetan wild barley L47 and L48 (Table 2; Table S4). When considering the six INDELs, the intron III *Bmy1.c* and *Bmy1.d* was not in line with the *Bmy1* amino acid nomenclature. For example, varieties carrying the intron III *Bmy1.c* allele may be the *Bmy1-Sd1c*, or *Bmy1-Sd2H* or *Bmy1-Sd5* amino acid haplotypes. Varieties carrying the intron III *Bmy1.d* allele may be the *Bmy1-Sd1a, or Bmy1-Sd2Ha* or *Bmy1-Sd3* amino acid haplotypes. As a consequence, the intron III haplotypes might not properly reflect the amino acid haplotypes of the varieties.

Two new deletions in the promoter region were discovered in this study. The first one was an 11 bp ‘TGAGAAGTGAA’ deletion in Chinese landraces z043 and m279. The second one was a 4 bp ‘TCTA’ deletion in Chinese landrace W127, Tibetan wild barley L35 and L68. In addition to the 11 bp and 4bp INDELs, there was a third 92 bp INDEL in the promoter region. The landrace m279 was least similar to other landraces and wild barleys (Figure 2). Comprehensive comparative studies of the *Bmy1* genomic sequence found that m279 was most similar to Adorra and was distinguishable at the 11 bp deletion in the promoter region. This 11 bp distinction might explain the moderate β-amylase activity of m279 found in this study, which was higher than Adorra-like cultivars [23]. It was possible that m279 and Adorra clustered with European barleys like Stander carrying the *Bmy1-Sd4* allele at the *Bmy1* genomic DNA level. The m279, Adorra and European barleys have the same intron III deletion pattern (Table 2), and the *Bmy1-Sd2L* together with *Bmy1-Sd4* alleles are the most common *Bmy1* haplotypes appearing in the European barley germplasm [5], [9]. In other words, we discovered the haplotypes representing European barleys in our eight barley accessions and this again illustrates the diversity and richness of the gene pool in Chinese barley germplasm. The 92 bp deletion in the promoter region was found in the Tibetan wild barley L47. From the Multiplex-ready marker screening, the landrace z043 was on the border of two large groups by their INDELs. Barleys z043 and L47 had the same cDNA and amino acid sequences as Harrington and Morex. Harrington is a world standard for high malt quality, and β-amylase activities of approximately 1490 U g^–1^ have been reported [7]. *Bmy1* sequence comparisons of L47 and z043 indicated major differences. z043 had the 11 bp deletions and 92 bp insertions in the promoter region, and 38 bp insertions in intron III regions, while L47 had the 11 bp and 21 bp insertions in intron III region. The 92 bp insertions were the largest fragment among the 92 bp, 38 bp, 11 bp and 21 bp differences observed in z043 and L47; one possible assumption is that the 92 bp insertions in the promoter region associated most with β-amylase activity in z043. The presence of the 92 bp could have a negative impact on β-amylase activity in z043. The 92 bp fragment may partially explain why β-amylase activity in the *Bmy1-Sd2L* and *Bmy1-Sd2H+* haplotypes was higher than *Bmy1-Sd1* and *Bmy1-Sd2H–* haplotypes [15]. That is, *Bmy1-Sd1* and *Bmy1-Sd2H–* haplotypes have 92 bp deletions, while *Bmy1-Sd2L* and *Bmy1-Sd2H+* haplotypes have 92 bp insertions. Hence, when varieties have the same amino acid composition, the 92 bp deletion in the promoter region seemed to have a higher β-amylase activity. Another reason that the β-amylase activity of z043 was lower than L47 might emerge from the full *Bmy1* sequence. As it was shown in the evolutionary tree, the *Bmy1* sequence of z043 was very close to the North American feed barley Strider, which was described as having remarkably low β-amylase activity [6]. The intron III INDELs of L47 differed from PI296897 at one INDEL with 6 bp insertions, and the β-amylase activity of PI296897 was reportedly higher than Harrington [22]; perhaps the 6 bp insertion in the intron III negatively affects β-amylase activity when varieties have the same amino acid compositions. However, for the Tibetan wild barley L48, which had the same amino acid composition as L35 and L68, but differed at intron III due to a 6 bp insertion, had higher β-amylase activity. As a consequence, no conclusion could be drawn on the function of the 6 bp insertions in the intron III region, but the associations between the 6 bp INDEL in intron III and β-amylase activity are worth further investigation, as the novel intron III composition identified in L47 and L48 might provide new sources for malting quality improvement.Without the expression data for *Bmy1* genes in the above mentioned accessions, it was too early to conclude the promoter region and the introns probably had more effect on β-amylase than amino acid substitutions [26], [27].

The observed β-amylase activity in the selected accessions agreed with previous research which suggested a range of β-amylase activity in Chinese landraces from 93 to 2372 U g^–1^ [12]. Compared with β-amylase activity in Adorra-like, Haruna Nijo-like and PI296897-like cultivars [22], the lowest β-amylase activity observed in z043 was higher than Adorra-like cultivars and the β-amylase activity of m279, L35 and L47 was similar to Haruna Nijo. The wild barley PI 296897 reportedly has higher β-amylase enzyme activity than Haruna Nijo [4], and we observed higher β-amylase activity in W127 which was higher than PI 296897. At the genomic DNA level, our landraces and wild accessions were clustered with Haruna Nijo and wild barleys (Figure 2). This result agreed with the findings that *Bmy1* haplotypes were clustered by the geographic origin of the germplasm [6]. The current study demonstrated that the Chinese landraces and Tibetan wild barley were close to wild barleys, in support of Tibet as a centre of diversity for barley germplasm [10]. Meanwhile, the germplasm with higher β-amylase activity than Haruna Nijo provides a new source for selection of high β-amylase activity and thermostability for brewing industries in Asia and North America. Germplasm carrying the *Bmy1-Sd2L* haplotype like m279 provides a source for selection of low β-amylase activity which may be suitable for the European brewing style.

### Nomenclature of *Bmy1* haplotypes

Based on the nomenclature promoted by Chiapparino et al. [9] and clarified by Evans et al. [21], seven *Bmy1* alleles have been classified namely *Bmy1-Sd1a*, *Bmy1-Sd1b*, *Bmy1-Sd2L*, *Bmy1-Sd2H*, *Bmy1-Sd2Ha*, *Bmy1-Sd3* and *Bmy1-Sd4*. *Bmy1-Sd1* and *Bmy1-Sd2* were identified from commercial malting barley (*Hordeum vulgare*) and *Bmy1-Sd3* was discovered from wild barley *Hordeum spontaneum*. Although the *Bmy1* genomic sequence of wild barley Ashqelon differed from all the classified *Bmy1* alleles, the *Bmy1* allele it carries has not yet been defined [7]. *Bmy1-Sd1a* contains C115, E165, V233, S347 and A430; *Bmy1-Sd1b*: C115, E165, V233, S347 and V430; *Bmy1-Sd2L* contains R115, D165, V233, L347 and V430; *Bmy1-Sd2H* contains R115, D165, A233 and S347 and V430; *Bmy1-Sd3* contains R115, E165, A233, S347 and V430; and *Bmy1-Sd4* contains R115, D165, V233, S347 and V430. In this case, Tibetan wild barley L35, L48 and L68 could be the *Bmy1-Sd2H* haplotype by nomenclature. This result is in accordance with a previous study that Asian barley accessions were dominated by the *Bmy1-Sd2H* allele [5], and the amino acid substitution of V233 to A233 is specific to *Bmy1-Sd2H* [21]. The Israeli wild barley L46 contains C115, E165, A233, S347 and V430 which is unlike the *Bmy1-Sd1b* allele only at A233 (Table 1). Meanwhile, L46 has the same intron III deletion pattern as *Bmy1-Sd2H* (Table 2), but a low β-amylase activity which contrasts with the feature of *Bmy1-Sd2H*. Considering the low β-amylase activity of L46 and its similarities to Strider; we could classify the Israeli wild barley L46 as *Bmy1-Sd1c* allele. An L347 was identified in the Chinese landrace m129, and L347 was described to be specific to the *Bmy1-Sd2L* allele [1]. Meanwhile, the amino acid composition of m279 was the same as the *Bmy1-Sd2L* allele. The composition of C115, D165, V233, S347 and V430 was present in both landraces and wild barley, with the wild barley PI 296897 carrying a *Bmy1-Sd2Ha* allele [9]. Besides the amino acid composition, wild barley Ashqelon has higher β-amylase activity than PI296897, Harrington and Legacy [19]; therefore, we would classify Ashqelon and W127 as the *Bmy1-Sd5* of *Bmy1* gene.

Kaneko et al. [16] reported eight β-amylase-less mutants in Tibetan landraces. The four Tibetan wild barleys had large differences in *Bmy1* genomic sequences and enzyme activity (Table S4) indicating that Tibetan wild barley has high *Bmy1* genetic variation. This result extends our previous insight that the evolution process of Tibetan barley to Chinese landraces could be explored from the structure of Tibetan barley. These results contrasted those of Zhang et al. [13] that *Bmy1* genetic variation in Tibetan barley was quite low based on Cleaved Amplified Polymorphic Sequence (CAPS) analyses.

### Molecular marker breeding for desired β-amylase activity

Molecular markers are the fundamental tools to assess germplasm for genes of interest. As a result, the accuracy of molecular marker selection decides the efficiency of marker-assisted selection. Knowing the presence of the *Bmy1* alleles in the parents is the first step towards breeding cultivars with desired traits. By far, we have identified almost all the *Bmy1* haplotypes described in the literature (Tables 1 and 2). By comparing the SNPs in the cDNA sequences and amino acid substitutions in the protein sequence, we could easily design molecular markers to discriminate germplasm carrying different *Bmy1* haplotypes. For example, the SNPs of SNP^1357^, SNP^1462^ and SNP^1552^ in the cDNA have only been reported in Israeli wild barley L46, Tibetan wild barley L48 and North American barley Strider [6]. Considering SNP^1357^, SNP^1462^ and SNP^1552^ appeared together, the usage of any SNP^1357^, SNP^1462^ and SNP^1552^ substitutions could be enough for the first selection of germplasm to identify unique *Bmy1* alleles in breeding programs, especially with the germplasm collected in Asia.

Another example was the germplasm screening for haplotypes similar to one another: either of the SNP^1357^, SNP^1462^ or SNP^1552^ substitutions could distinguish the *Bmy1-Sd1a* from *Bmy1-Sd1b* allele. An SNP^698^ could easily distinguish *Bmy1-Sd1c* from two other *Bmy1* alleles. Meanwhile, the 126 bp INDEL is the most convenient marker to distinguish between the *Bmy1.a* and *Bmy1.b* alleles, which are the only two introns III alleles present in the North American cultivars [7]. Paris et al. [23] used SNPs at positions SNP^495^ and SNP^698^ in the cDNA to discern *Bmy1-Sd1*, *Bmy1-Sd2L* and *Bmy1-Sd2H*. Malysheva and Roder [5] used just SNP^698^ to distinguish *Bmy1-Sd2H* and *Bmy1-Sd3* from the rest. For selection of the *Bmy1-Sd2L* allele, either SNP^1040^ or SNP^1581^ in the cDNA sequence is enough to distinguish the remaining haplotypes. For the selection of the *Bmy1-Sd3* allele, either SNP^185^ or SNP^1414^ is sufficient to differentiate between the remaining haplotypes. In order to distinguish the alleles between *Bmy1-Sd2H*, *Bmy1-Sd2Ha* and *Bmy1-Sd5* in this study, we used the combination of SNP^343^ and SNP^1293^ whereby SNP^343^ discerns the *Bmy1-Sd2H* and *Bmy1-Sd5* alleles, and SNP^1293^ differentiates the *Bmy1-Sd2H* and *Bmy1-Sd2Ha* alleles.

## Conclusions

Novel genetic variation is essential for better genetic gains in breeding programs. This study investigated genetic variation in the most well understood gene *Bmy1* to evaluate genetic potential for improvement of malting quality in Chinese landraces and Tibetan wild barley. Variations were observed in both β-amylase activity and sequences of the introns, exons and promoters. By comparing the known seven types of *Bmy1* alleles, two new *Bmy1* haplotypes, *Bmy1-Sd1c* and *Bmy1-Sd5*, were identified.We revealed one new substitution at position 387 from Chinese landrace W127 in addition to the existing 12 amino acid substitutions. Novel INDELS were also identified in the promoter region and intron III. Five of the eight accessions have the *Bmy1-Sd2H* allele which is known to correspond with high thermostability and β-amylase activity, and is desired in the brewing industry for high DP and fermentability. The new *Bmy1* alleles and SNPs, along with the diversity of β-amylase activity provide us with diverse resources for breeding barley for different brewing styles. Barley germplasm with the amino acid composition of C115, D165, V233, S347 and V430 was found and may be an ideal resource for higher β-amylase activity and thermostability. We conclude that the gene pool of Chinese barley germplasm may provide unique potential to improve malting quality.

## Supporting Information

Table S1 The accession number, Chinese name and geographic origin of barley used in this study (The eight accessions sequenced for *Bmy1* are in bold). * This number is in accordance with Figure 1.
(DOC)

Table S2 Primer sequences of Multiplex-ready assays. ^a^ The start and end positions of the primers referred to Table S4.
(DOC)

Table S3 Primers’ sequence, position and PCR conditions amplifying the whole *Bmy1* gene.
(DOC)

Table S4 Alignment of full length *Bmy1* gene in 8 accessions and that of 5 reference varieties. The *Bmy1* haplotypes and GeneBank accessions numbers are as follows. Sd1: Morex (EU589328), Harrington (FJ161079), HA52 (AJ301645), Strider (EU589327) z043 (KF302668), L46 (L302672) and L47 (KF302673); Sd2H Haruna Nijo (D49999), PI296897 (AF061204), L35 (KF302671), L48 (KF302674) and L68 (KF302675); Sd2L: Adorra (AF061203), Hiproly (X52321) and m279 (KF302670); Sd3: AB75; Sd4: Steptoe (EF175470.1), Orca (EF175468), Legacy (FJ161080), Stander (EF175469), Tango (EF175471), UC958 (EF175472.1), UC960 (EF175473.1), Schooner (AF300799), Franklin (AF300800); Sd5: Ashqelon (FJ161078) and W127 (KF302669). The unique nucleotide polymorphism identified in this study was marked in rectangles. The exons of the *Bmy1* gene were underlined with sold line.
(DOC)

Table S5 INDELs positions and unique SNPs identified in *Bmy1* compared to 7 *Bmy1* haplotypes.
(DOC)

Table S6 The position, size and sequence of *Bmy1* genomic INDELs (insertion/deletion).
(DOC)
