# Supplementary material for: Discovery of Novel Bmy1 Alleles Increasing β-Amylase Activity in Chinese Landraces and Tibetan Wild Barley for Improvement of Malting Quality via MAS
Source: PLoS One. 2013 Sep 3;8(9):e72875. doi: 10.1371/journal.pone.0072875 (PMC3760831; doi:10.1371/journal.pone.0072875)
Supplement: Table S1 — The accession number, Chinese name and geographic origin of barley used in this study (The eight accessions sequenced for Bmy1 are in bold). * This number is in accordance with Figure 1. (DOC) [file pone.0072875.s001.doc]

**Table S1.** The accession number, Chinese name and geographic origin of barley used in this study (The eight accessions sequenced for *Bmy1* are in bold).

| *Number | Chinese Name | Origination | row | Number | Chinese Name | Origination | row |
| --- | --- | --- | --- | --- | --- | --- | --- |
| A38 | heishuizaoshuziqingke | Aba, Sichuan | six | A483 | beijingmidamai | Beijing | six |
| A39 | ababaisileng | Aba, Sichuan | four | A484 | guangtoulang | Sichuan | six |
| A41 | zhanglahuiqingke | Ganzi, Sichuan | six | A488 | heidudamai | Sichuan | six |
| A42 | 7368-2(Kunlun2xzaoshu43) | Sichuan | six | A491 | xideerhao | Germany | two |
| **z043** | **zaofengyihao** | **Ganzi, Sichuan** | **six** | A529 | Chuan 89-145 | Ya’an, Sichuan | six |
| A45 | 83-5039 | Ya'an , Sichuan | six | A582 | wumangzidamai | Ya'an, Sichuan | six |
| A46 | heiqingke | Aba, Sichuan | six | A585 | yuanyukangqingerhao | Ganzi, Sichuan | six |
| A47 | nantameiguihong | Linzhi, Tibet | six | A614 | zhenhaihunzazhong | Zhejiang | two |
| A48 | shuangpei | Linzhi, Tibet | two | A617 | tiantaierlengdamai | Hanglai, Sichuan | two |
| A51 | 82-6009 | Ya'an , Sichuan | two | A619 | yiwuerlengdamai | Yiwu, Zhejiang | two |
| A52 | baxiziqingke | Baxi, Tibet | six | A620 | shouchangwugongmai | Shouchang, Jiangxi | two |
| A53 | qinglanglvqingke | Qianglang, Tibet | six | **L35** | **HUA01** | **Tibet** | **two** |
| A54 | baxihuiqingke | Baxi, Tibet | six | L36 | HUA03 | Tibet | two |
| A55 | rangkuangduanmangheiliuleng | Ganzi, Sichuan | six | L37 | HUA04 | Tibet | six |
| A59 | nantazimanghuake | Linzhi, Tibet | six | L38 | HUA04 | Tibet | two |
| A86 | zilibashitianqingke | Yunnan | six | L39 | HUA09 | Tibet | six |
| A87 | huangqingke | Yunnan | six | L41 | HUA011 | Tibet | six |
| A88 | qinyangqumai | Henan | two | L43 | HUA018 | Tibet | two |
| A90 | MaiteB23 | Sichuan | two | L44 | HUA022 | Tibet | two |
| A91 | zhichengliuleng | Ganzi, Sichuan | six | L45 | HUA029 | Tibet | two |
| **W127** | **W84 - 127** | **Sichuan** | **two** | **L46** | **HUA032** | **Isreal** | **two** |
| A176 | tongnan83 – 7 – 4 | Tongnan, Sichuan | two | **L47** | **HUA033** | **Tibet** | **two** |
| A248 | shisuijinhuang | Japan | two | **L48** | **HUA052** | **Tibet** | **two** |
| A253 | baodingmidamai | Baoding, Hebei | six | L49 | HUA079 | Tibet | two |
| A254 | aiganbaiyang | Mianyang, Sichuan | six | L50 | HUA094 | Tibet | two |
| A256 | abaheiqingke | Aba, Sichuan | six | L51 | HUA0230 | Tibet | two |
| A257 | dulihuang | Aba, Sichuan | six | L52 | HUA0246 | Tibet | six |
| A258 | fengtianbaidamai | Liaoning | six | L53 | HUA0334 | Tibet | two |
| A259 | yeshengerlenghei | Ganzi, Sichuan | two | L54 | HUA0544 | Tibet | two |
| A262 | nanchongguangtoumidamai | Nanyong, Sichuan | six | L55 | HUA545 | Tibet | two |
| A263 | huxianzhuerchong | Luzhou, Sichuan | six | L56 | HUA0546 | Tibet | two |
| A268 | ganzi809 | Ganzi, Sichuan | six | L57 | HUA0547 | Tibet | two |
| A274 | bameiliulenghei | Litang, Sichuan | six | L58 | HUA0548 | Tibet | two |
| **m279** | **maerkangsileng** | **Litang, Sichuan** | **six** | L59 | HUA0551 | Tibet | two |
| A284 | dengfengdamai | Dengfeng, Henan | six | L60 | HUA0551 | Tibet | two |
| A285 | muerzong | Ganzi, Sichuan | six | L62 | HUA0564 | Tibet | two |
| A293 | liulengtuomangmai | Ganzi, Sichuan | six | L63 | HUA0567 | Tibet | two |
| A298 | 231 | Ya'an , Sichuan | six | L64 | HUA0569 | Tibet | two |
| A334 | haiandamai | Mianyang, Sichuan | six | L67 | HUA0609 | Tibet | two |
| A350 | heishuizaoshuziqingke | Aba, Sichuan | six | **L68** | **HUA0640** | **Tibet** | **six** |
| A383 | shengxianwumangliuleng | Chengxian, Zhejiang | six | L69 | HUA0641 | Tibet | two |
| A386 | shengxian209 | Chengxian, Zhejiang | two | L70 | HUA0642 | Tibet | two |
| A471 | erlengziqingkedami | Ya'an , Sichuan | two | L72 | HUA0650 | Tibet | two |
| A475 | qianqiluo | Sichuan | six | L73 | HUA0651 | Tibet | two |
| A477 | magemudamai | Sichuan | six | L74 | HUA0701 | Tibet | two |
| A478 | zhoumaiyihao | Zhoushan, Zhejiang | two |  |  |  |  |

* This number is in accordance with Figure 1.
