# Supplementary material for: Discovery of Novel Bmy1 Alleles Increasing β-Amylase Activity in Chinese Landraces and Tibetan Wild Barley for Improvement of Malting Quality via MAS
Source: PLoS One. 2013 Sep 3;8(9):e72875. doi: 10.1371/journal.pone.0072875 (PMC3760831; doi:10.1371/journal.pone.0072875)
Supplement: Table S2 — Primer sequences of Multiplex-ready assays. a The start and end positions of the primers referred to Table S4. (DOC) [file pone.0072875.s002.doc]

**Table S2**. Primer sequences of Multiplex-ready assays.

| hvSSR | Forward (5’ → 3’)  Reverse (5’ → 3’) | INDEL | Expected  size | Allele Size  range | Sd1 | Sd2L | Sd2H- | Sd2H+ | Sd3 | Unknown  1 | Unknown  1 | Sd2H |
| --- | --- | --- | --- | --- | --- | --- | --- | --- | --- | --- | --- | --- |
| hv1006 | Cagacafttcacccataagct  tgctgctgctttgaagtctgc | 9a | 458 | 437-458 | 437 | 458 | 458 | 458 | 458 | 437 | 458 | 458 |
| hv1010 | Cctcaaaatttgcaggtagcat  tggtgtgtaaaccattgccttc | 3 | 304 | 209-304 | 209 | 298 | 298 | 207 | 298 | 207 | 304 | 299 |
| hv1013 | Gatgagcgcaccagaagaact  Ccctctctccatccagcactc | 6 | 266 | 222-266 | 266 | 264 | 264 | 264 | 255 | 222 | 222 | 264 |
| hv1014 | Ttgttgatttgcaggtgccta  Tcgatatttctggccctgatc | 7 | 147 | 144-147 | 147 | 147 | 147 | 147 | 147 | 144 | 144 | 147 |
| hv1015 | Caattgctgaaaggcatgaaa  gccacccgagcaatgagatac | 1b | 369 | 352-369 | 366 | 369 | 365 | 365 | 365 | 366 | 364 | 352 |
| hv1016 | Accttgactacacttccattgttg  Cgaacctgttgttcacgctca | 4 | 99 | 95-99 | 95 | 99 | 99 | 99 | 99 | 95 | 99 | 99 |
| hv1018 | Tgtggctgtgacagatgtatgc  Catttgggtgtttgtttcctga | 8b | 470 | 344-470 | 344 | 470 | 344 | 470 | 344 | 344 | 470 | 344 |
| hv1019 | Gaagatctgccgtccaggtta  rgctaggttttgttyctttgct | 5b | 185 | 170-185 | 185 | 170 | 170 | 170 | 170 | 185 | 170 | 170 |
| hv1020 | Gggtggcatccaaattttcc  Ttggctcmmgggagaatatgct | 9b | 272 | 244-278 | 244 | 265 | 272 | 265 | 272 | 244 | 265 | 272 |
| hv1021 | Gggtggcatccaaattttcc  Cacatcyaaatgctacctgcaa | 2c | 303 | 277-303 | 293 | 294 | 277 | 296 | 277 | 295 | 301 | 303 |
| hv1022 | Tatgattcattgaccccrcacg  Ccttgcatcaaggtttgtgcta | 9b | 272 | 244-278 | 244 | 265 | 272 | 265 | 272 | 244 | 265 | 272 |
| hv1023 | Cagacagttcacccataagct  Ccttgcatcaaggtttgtgcta | 9c | 130 | 109-130 | 109 | 130 | 130 | 130 | 130 | 109 | 130 | 130 |
