# Supplementary material for: Discovery of Novel Bmy1 Alleles Increasing β-Amylase Activity in Chinese Landraces and Tibetan Wild Barley for Improvement of Malting Quality via MAS
Source: PLoS One. 2013 Sep 3;8(9):e72875. doi: 10.1371/journal.pone.0072875 (PMC3760831; doi:10.1371/journal.pone.0072875)
Supplement: Table S3 — Primers’ sequence, position and PCR conditions amplifying the whole Bmy1 gene. (DOC) [file pone.0072875.s003.doc]

**Table S3**. Primers’ sequence, position and PCR conditions amplifying the whole *Bmy1* gene.

| Primer  name | Forward sequence (5’ → 3’) | Reverse sequence (5’ → 3’) | *Start  bp | *End  bp | PCR conditions  Tm (°C) |
| --- | --- | --- | --- | --- | --- |
| P1 | ACATCATCTTGAGAACGTC | CATCTATGAACCATTGCATGG | 1 | 418 | 62-58 |
| P2 | GTGAGGCACATTCTCATTTG | CCTTCTAGAGGAGTCTAG | 293 | 860 | 60-55 |
| P3 | GATGATTGTATTAGTGTCGCA | GACGCACACATCCTGACT | 449 | 1015 | 62-58 |
| P4 | CTAGACTCCTCTAGAAGGCA | GACGTAGACTTGGACATAG | 843 | 1400 | 62-58 |
| P5 | GCATCATCCATAGCCAGCAT | GACGTTGCCACCACACTG | 1333 | 1865 | 60-55 |
| P6 | GTCATGGTAGACGTCTGGTG | caatagaaggctgcctgcatc | 1716 | 2324 | 60-55 |
| P7 | GCCTAGAGCGGATGGGT | GTCCCACTTCAATGTCGA | 2217 | 2501 | 60-55 |
| P8 | CAGATGTATGCCCGATTACATG | GCGATCACATATTCACATGTTG | 2414 | 2799 | 62-58 |
| P9 | CGATGTAACTCAACATGTGA | CTGTGTCATCTAGCCTTG C | 2768 | 3419 | 60-55 |
| P10 | GACAGTTCACCCATAAGCTC | CCAAGATCCTGTCACCGTG | 3314 | 3905 | 62-58 |
| P11 | GTACAATGACACTCCCGAGA | CTCTGGTTGATTCCATGAGG | 3782 | 4679 | 62-58 |
| P12 | CTCAGGTGTTGAGTGCTGGAT | ATTCATTACATGGTGGCAGGGA | 4553 | 5194 | 62-58 |

* The start and end positions of the primers referred to Table S4.
