# Supplementary material for: Discovery of Novel Bmy1 Alleles Increasing β-Amylase Activity in Chinese Landraces and Tibetan Wild Barley for Improvement of Malting Quality via MAS
Source: PLoS One. 2013 Sep 3;8(9):e72875. doi: 10.1371/journal.pone.0072875 (PMC3760831; doi:10.1371/journal.pone.0072875)
Supplement: Table S4 — Alignment of full length Bmy1 gene in 8 accessions and that of 5 reference varieties. The Bmy1 haplotypes and GeneBank accessions numbers are as follows. Sd1: Morex (EU589328), Harrington (FJ161079), HA52 (AJ301645), Strider (EU589327) z043 (KF302668), L46 (L302672) and L47 (KF302673); Sd2H Haruna Nijo (D49999), PI296897 (AF061204), L35 (KF302671), L48 (KF302674) and L68 (KF302675); Sd2L: Adorra (AF061203), Hiproly (X52321) and m279 (KF302670); Sd3: AB75; Sd4: Steptoe (EF175470.1), Orca (EF175468), Legacy (FJ161080), Stander (EF175469), Tango (EF175471), UC958 (EF175472.1), UC960 (EF175473.1), Schooner (AF300799), Franklin (AF300800); Sd5: Ashqelon (FJ161078) and W127 (KF302669). The unique nucleotide polymorphism identified in this study was marked in rectangles. The exons of the Bmy1 gene were underlined with sold line. (DOC) [file pone.0072875.s004.doc]

**Supplementary table 4** Alignment of full length *Bmy1* gene in 8 accessions and that of 5 reference varieties

**....|....| ....|....| ....|....| ....|....| ....|....| ....|....| ....|....| ....|....| ....|....| ....|....|**

**10 20 30 40 50 60 70 80 90 100**

**Morex**  ---------- ---------- ---------- ---------- ---------- ---------- ---------- ---------- ---------- ----------

**Harrington**  ACATCATCTT GAGAACGTCT CCTCTTACTG AATGCCAATT GCTGAAAGGC ATGGAATACA TTAAGGAAAA ATGCAAACAC AAATGTCAAG TCTGAGACTT

**HA52**  ACATCATCTT GAGAACGTCT TCTCTTACTG AATGCCAATT GCTGAAAGGC ATGGAATACA TTAAGGAAAA ATGCAAACAC AAATGTCAAG TCTGAGACTT

**z043**  ACATCATCTT GAGAACGTCT TCTCTAACTG AATGCCAATT GCTGAAAGGC ATGAAATAAA TTAAGGAAAC TTGCAAACAC AAATGTCAAG TCTGAGACTT

**L47**  ACATCATCTT GAGAACGTCT CCTCTTACTG AATGCCAATT GCTGAAAGGC ATGGAATACA TTAAGGAAAA ATGCAAACAC AAATGTCAAG TCTGAGACTT

**Strider**  ---------- ---------- ---------- ---------- ---------- ---------- ---------- ---------- ---------- ----------

**W127**  ACATCATCTT GAGAACGTCT TCTCTAACTG AATGCCAATT GCTGAAAGGC ATAAAATAAA TTAAGGAAAC TTGCAAACAC AAATGTCAAG TCTGAGACTT

**Adorra**  ACATCATCTT GAGAACGTCT TCTCTAACTG AATGCCAATT GCTGAAAGGC ATGGAATACA TTAAGGAAAA ATGCAAACAC AAATGTCAAG TCTGAGACTT

**Haruna Nijo**  ACATCATCTT GAGAACGTCT TCTCTAACTG AATGCCAATT GCTGAAAGGC ATAAAATAAA TTAAGGAAAC TTGCAAACAC AAATGTCAAG TCTGAGACTT

**L35**  ACATCATCTT GAGAACGTCT TCTCTAACTG AATGCCAATT GCTGAAAGGC ATAAAATAAA TTAAGGAAAC TTGCAAACAC AAATGTCAAG TCTGAGACTT

**L48**  ACATCATCTT GAGAACGTCT TCTCTAACTG AATGCCAATT GCTGAAAGGC ATAAAATAAA TTAAGGAAAC TTGCAAACAC AAATGTCAAG TCTGAGACTT

**L68**  ACATCATCTT GAGAACGTCT TCTCTAACTG AATGCCAATT GCTGAAAGGC ATAAAATAAA TTAAGGAAAC TTGCAAACAC AAATGTCAAG TCTGAGACTT

**PI296897**  ACATCATCTT GAGAACGTCT TCTCTAACTG AATGCCAATT GCTGAAAGGC ATGAAATAAA TTAAGGAAAC TTGCAAACAC AAATGTCAAG TCTGAGACTT

**AB75**  ACATCATCTT GAGAACGTCT TCTCTAACTG AATGCCAATT GCTGAAAGGC ATAAAATAAA TTAAGGAAAC TTGCAAACAC AAATGTCAAG TCTGAGACTT

**m279**  ACATCATCTT GAGAACGTCT TCTCTAACTG AATGCCAATT GCTGAAAGGC ATGAAATAAA TTAAGGAAAC TTGCAAACAC AAATGTCAAG TCTGAGACTT

**Legacy**  ACATCATCTT GAGAACGTCT TCTCTAACTG AATGCCAATT GCTGAAAGGC ATGAAATAAA TTAAGGAAAC TTGCAAACAC AAATGTCAAG TCTGAGACTT

**Orca**  ---------- ---------- ---------- ---------- ---------- ---------- ---------- ---------- ---------- ----------

**Tango**  ---------- ---------- ---------- ---------- ---------- ---------- ---------- ---------- ---------- ----------

**UC958**  ---------- ---------- ---------- ---------- ---------- ---------- ---------- ---------- ---------- ----------

**UC960**  ---------- ---------- ---------- ---------- ---------- ---------- ---------- ---------- ---------- ----------

**Steptoe**  ---------- ---------- ---------- ---------- ---------- ---------- ---------- ---------- ---------- ----------

**Stander**  ---------- ---------- ---------- ---------- ---------- ---------- ---------- ---------- ---------- ----------

**Ashqelon**  ACATCATCTT GAGAACGTCT TCTCTAACTG AATGCCAATT GCTGAAAGGC ATGAAATAAA TTAAGGAAAC TTGCAAACAC AAATGTCAAG TCTGAGACTT

**L46**  ACATCATCTT GAGAACGTCT TCTCTAACTG AATGCCAATT GCTGAAAGGC ATGAAATAAA TTAAGGAAAC TTGCAAACAC AAATGTCAAG TCTGAGACTT

**....|....| ....|....| ....|....| ....|....| ....|....| ....|....| ....|....| ....|....| ....|....| ....|....|**

**110 120 130 140 150 160 170 180 190 200**

**Morex**  ---------- ---------- ---------- ---------- ---------- ---------- ---------- ---------- ---------- ----------

**Harrington**  GAACTCTAGT GGACACGTTT GAACATTAAT GTGCGTTTTT GGTGAAA-GT GAAAAAATAG TTGACAACCT TGTGAAATGA TTCAATTCAA ACCAAATGCA

**HA52**  GAACTCTAGT GGACACGTTT GAACATTAAT GTGCGTTTTT GGTGAAAAGT GAAAAAATAG TTGACAACCT TGTGAA-TGA TTCAATTCAA ACCAAATGCA

**z043**  GAACTCTAGT TGACACGTTT GAACATTAAT GCGCGGTTTT GGTGAAA-GT GAAAAAACAG TTGACATCCT TGTGAA-TGA ATCAATTCAA ACCAAATGCA

**L47**  GAACTCTAGT GGACACGTTT GAACATTAAT GTGCGTTTTT GGTGAAA-GT GAAAAAATAG TTGACAACCT TGTGAAATGA TTCAATTCAA ACCAAATGCA

**Strider**  ---------- ---------- ---------- ---------- ---------- ---------- ---------- ---------- ---------- ----------

**W127**  GAACTCTAGT GGACACGTTT GAACATTAAT GTGCGGTTTT GGTGAAA-GT GAAAAAACAG TTGACATCCT TGTGAA-TGA ATCAATTCAA ACCAAATGCA

**Adorra**  GAACTCTAGG GGACACGTTT GAACATTAAC GTGTGTTTTT GGTGAAA-GT GAAAAAATAG TTGACAACCT TGTGAAATGA TTCAGTTCAA ACCAAATGCA

**Haruna Nijo**  GAACTCTAGT GGACACGTTT GAACATTAAT GTGCGGTTTT GGTGAAA-GT GAAAAAACAG TTGACATCCT TGTGAA-TGA ATCAATTCAA ACCAAATGCA

**L35**  GAACTCTAGT GGACACGTTT GAACATTAAT GTGCGGTTTT GGTGAAA-GT GAAAAAACAG TTGACATCCT TGTGAA-TGA ATCAATTCAA ACCAAATGCA

**L48**  GAACTCTAGT GGACACGTTT GAACATTAAT GTGCGGTTTT GGTGAAA-GT GAAAAAACAG TTGACATCCT TGTGAA-TGA ATCAATTCAA ACCAAATGCA

**L68**  GAACTCTAGT GGACACGTTT GAACATTAAT GTGCGGTTTT GGTGAAA-GT GAAAAAACAG TTGACATCCT TGTGAA-TGA ATCAATTCAA ACCAAATGCA

**PI296897**  GAACTCTAGT GGACACGTTT GAACATTAAT GTGCGGTTTT GGTGAAA-GT GAAAAAACAG TTGACATCCT TGTGAA-TGA ATCAATTCAA ACCAAATGCA

**AB75**  GAACTCTAGT GGACACGTTT GATCATTAAT GTGCGGTTTT GGTGAAA-GT GAAAAAACAG TTGACATCCT TGTGAA-TGA ATCAATTCAA ACCAAATGCA

**m279**  GAACTCTAGT TGACACGTTT GAACATTAAT GCGCGGTTTT GGTGAAA-GT GAAAAAACAG TTGACATCCT TGTGAA-TGA ATCAATTCAA ACCAAATGCA

**Legacy**  GAACTCTAGT GGACACGTTT GAACATTAAT GCGCGGTTTT GGTGAAA-GT GAAAAAACAG TTGACATCCT TGTGAA-TGA ATCAATTCAA ACCAAATGCA

**Orca**  ---------- ---------- ---------- ---------- ---------- ---------- ---------- ---------- ---------- ----------

**Tango**  ---------- ---------- ---------- ---------- ---------- ---------- ---------- ---------- ---------- ----------

**UC958**  ---------- ---------- ---------- ---------- ---------- ---------- ---------- ---------- ---------- ----------

**UC960**  ---------- ---------- ---------- ---------- ---------- ---------- ---------- ---------- ---------- ----------

**Steptoe**  ---------- ---------- ---------- ---------- ---------- ---------- ---------- ---------- ---------- ----------

**Stander**  ---------- ---------- ---------- ---------- ---------- ---------- ---------- ---------- ---------- ----------

**Ashqelon**  GAACTCTAGT GGACACGTTT GATCATTAAT GTGCGGTTTT GGTGAAA-GT GAAAAAACAG TTGACATCCT TGTGAA-TGA ATCAATTCAA ACCAAATGCA

**L46**  GAACTCTAGT TGACACGTTT GAACATTAAT GCGCGGTTTT GGTGAAA-GT GAAAAAACAG TTGACATCCT TGTGAA-TGA ATCAATTCAA ACCAAATGCA

**....|....| ....|....| ....|....| ....|....| ....|....| ....|....| ....|....| ....|....| ....|....| ....|....|**

**210 220 230 240 250 260 270 280 290 300**

**Morex**  ---------- ---------- ---------- ---------- ---------- ---------- ---------- ---------- ---------- ----------

**Harrington**  AAAATAAGAA GTGAAAACCC CATAATCGTT GTTTGTAGAA AACAAATAAC ACTTAAACTA ATGGGGTATT TTCTGAA--- AAACTATAAT TGGTGAGGCA

**HA52**  AAAATAAGAA GTGAAAACCC CATAATCGTT GTTTGTAGAA AACAAATAAC ACTTAAACTA ATGGGGTATT TTCTGAA--- AAACTATAAT TGGTGAGGCA

**z043**  AAAA------ -----AATCC CATAATCGTC GTTTGTAGAA AACAAATAAC ACTGAAACTA ATGGG-TATT TTCTGAG--- AAACTATAAT TGGTGAGGCA

**L47**  AAAATAAGAA GTGAAAACCC CATAATCGTT GTTTGTAGAA AACAAATAAC ACTTAAACTA ATGGGGTATT TTCTGAA--- AAACTATAAT TGGTGAGGCA

**Strider**  ---------- ---------- ---------- ---------- ---------- ---------- ---------- ---------- ---------- ----------

**W127**  AAAATGAGAA GTGAAAACCC CATAATCGTC GTTTGTAGAA AACAAATAAC ACTGAAACTA ATGGGGTATT TTCTGAG--- AAACTATAAT TGGTGAGGCA

**Adorra**  AAAATAAGAA GTGAAAACCC CATAATCGTT GTTTGTAGAA AACAAATAAC ACTTAAACTA ATGGGGTATT TTCTGAAAAA AAACTATAAT TGGTGAGGCA

**Haruna Nijo**  AAAATGAGAA GTGAAAACCC CATAATCGTC GTTTGTAGAA AACAAATAAC ACTGAAACTA ATGGGGTATT TTCTGAG--- AAACTATAAT TGGTGAGGCA

**L35**  AAAATGAGAA GTGAAAACCC CATAATCGTC GTTTGTAGAA AACAAATAAC ACTGAAACTA ATGGGGTATT TTCTGAG--- AAACTATAAT TGGTGAGGCA

**L48**  AAAATGAGAA GTGAAAACCC CATAATCGTC GTTTGTAAAG AACAAATAAC ACTGAAACTA ATGGGGTATT TTCTGAG--- AAACTATAAT TGGTGAGGCA

**L68**  AAAATGAGAA GTGAAAACCC CATAATCGTC GTTTGTAGAA AACAAATAAC ACTGAAACTA ATGGGGTATT TTCTGAG--- AAACTATAAT TGGTGAGGCA

**PI296897**  AAAATGAGAA GTGAAAACCC CATAATCGTC GTTTGTAGAA AACAAATAAC ACTGAAACTA ATGGGGTAAT TTCTGAG--- AAACTATAAT TGGTGAGGCA

**AB75**  AAAATGAGAA GTGAAAACCC CATAATCGTC GTTTGTAGAA AACAAATAAC ACTGAAACTA ATGGGGTATT TTCTGAAGAG AAACTATAAT TGGTGAGGCA

**m279**  AAAA------ -----AATCC CATAATCGTC GTTTGTAGAA AACAAATAAC ACTGAAACTA ATGGG-TATT TTCTGAG--- AAACTATAAT TGGTGAGGCA

**Legacy**  AAAATGAGAA GTGAAAATCC CATAATCGTC GTTTGTACAA AACAAATAAC ACTGAAACTA ATGGG-TATT TTCTGAG--- AAACTATAAT TGGTGAGGCA

**Orca**  ---------- ---------- ---------- ---------- ---------- ---------- ---------- ---------- ---------- ----------

**Tango**  ---------- ---------- ---------- ---------- ---------- ---------- ---------- ---------- ---------- ----------

**UC958**  ---------- ---------- ---------- ---------- ---------- ---------- ---------- ---------- ---------- ----------

**UC960**  ---------- ---------- ---------- ---------- ---------- ---------- ---------- ---------- ---------- ----------

**Steptoe**  ---------- ---------- ---------- ---------- ---------- ---------- ---------- ---------- ---------- ----------

**Stander**  ---------- ---------- ---------- ---------- ---------- ---------- ---------- ---------- ---------- ----------

**Ashqelon**  AAAATGAGAA GTGAAAACCC CATAATCGTC GTTTGTAGAA AACAAATAAC ACTGAAACTA ATGGGGTATT TTCTGAG--- AAACTATAAT TGGTGAGGCA

**L46**  AAAA------ -----AATCC CATAATCGTC GTTTGTAGAA AACAAATAAC ACTGAAACTA ATGGG-TATT TTCTGAG--- AAACTATAAT TGGTGAGGCA

**....|....| ....|....| ....|....| ....|....| ....|....| ....|....| ....|....| ....|....| ....|....| ....|....|**

**310 320 330 340 350 360 370 380 390 400**

**Morex**  ---------- ---------- ---------- ---------- ---------- ---------- ---------- ---------- ---------- ----------

**Harrington**  CATTCTCATT TGATTGGTTA GTTTAACTTC CTTGTCACAT TATTTTTTTC TAGAGTATCT CATTGCTCGG GTGGCATCCA AATTTTCCAT ACAAGAGCCC

**HA52**  CATTCTCATT TGATTGGTTA GTTTAACTTC CTTGTCACAT -A--TTTTTC TAGAGTATCT CATTGCTCGG GTGGCATCCA AATTTTCCAT ACAAGAGCCC

**z043**  CATTCTCATT TGATTGGTTA GTTTAACTTC CTTCTCACAT TATTTTTTCC TAGAGTATCT CATTGCTCGG GTGGCATCCA AATTTTCCAG ACAAGAGCCC

**L47**  CATTCTCATT TGATTGGTTA GTTTAACTTC CTTGTCACAT TATTTTTTTC TAGAGTATCT CATTGCTCGG GTGGCATCCA AATTTTCCAT ACAAGAGCCC

**Strider**  ---------- ---------- ---------- ---------- ---------- ---------- ---------- ---------- ---------- ----------

**W127**  CATTCTCATT TGATTGTTTA GTTTAACTTC CTTCTCACAT TATTTTTTCC TAGAGTATCT CATTGCTCGG GTGGCATCCA AATTTTCCAG ACAAGGGCCC

**Adorra**  CATTCTCATT TGATTGGTTA GTTTAACTTC CTTGTCACAT TATTTTTTTC TAGAGTATCT CATTGCTCGG GTGGCATCCA AATTTTCCAT ACAAGAGCCC

**Haruna Nijo**  CATTCTCATT TGATTGTTTA GTTTAACTTC CTTCTCACAT TATTTTTTCC TAGAGTATCT CATTGCTCGG GTGGCATCCA AATTTTCCAG ACAAGGGCCC

**L35**  CATTCTCATT TGATTGTTTA GTTTAACTTC CTTCTCACAT TATTTTTTCC TAGAGTATCT CATTGCTCGG GTGGCATCCA AATTTTCCAG ACAAGGGCCC

**L48**  CATTCTCATT TGATTGTTTA GTTTAACTTC CTTCTCACAT TATTTTTTCC TAGAGTATCT CATTGCTCGG GTGGCATCCA AATTTTCCAG ACAAGGGCCC

**L68**  CATTCTCATT TGATTGTTTA GTTTAACTTC CTTCTCACAT TATTTTTTCC TAGAGTATCT CATTGCTCGG GTGGCATCCA AATTTTCCAG ACAAGGGCCC

**PI296897**  CATTCTCATT TGATTGTTTA GTTTAACTTC CTTCTCACAT TATTTTTTCC TAGAGTATCT CATTGCTCGG GTGGCATCCA AATTTTCCAG ACAAGGGCC-

**AB75**  CATTCTCATT TGATTGTTTA GTTTAACTTC CTTCTCACAT TATTTTTTCC TAGAGTATCT CATTGCTCGG GTGGCATCCA AATTTTCCAG ACAAGGGCCC

**m279**  CATTCTCATT TGATTGTTTA GTTTAACTTC CTTCTCACAT TATTTTTTCC TAGAGTATCT CATTGCTCGG GTGGCATCCA AATTTTCCAG ACAAGGGCC-

**Legacy**  CATTCTCATT TGATTGTTTA GTTTAACTTC CTTCTCACAT TATTTTTTCC TAGAGTATCT CATTGCTCGG GTGGCATCCA AATTTTCCAG ACAAGGGCCC

**Orca**  ---------- ---------- ---------- ---------- ---------- ---------- ---------- ---------- ---------- ----------

**Tango**  ---------- ---------- ---------- ---------- ---------- ---------- ---------- ---------- ---------- ----------

**UC958**  ---------- ---------- ---------- ---------- ---------- ---------- ---------- ---------- ---------- ----------

**UC960**  ---------- ---------- ---------- ---------- ---------- ---------- ---------- ---------- ---------- ----------

**Steptoe**  ---------- ---------- ---------- ---------- ---------- ---------- ---------- ---------- ---------- ----------

**Stander**  ---------- ---------- ---------- ---------- ---------- ---------- ---------- ---------- ---------- ----------

**Ashqelon**  CATTCTCATT TGATTGTTTA GTTTAACTTC CTTCTCACAT TATTTTTTCC TAGAGTATCT CATTGCTCGG GTGGCATCCA AATTTTCCAG ACAAGGGCCC

**L46**  CATTCTCATT TGATTGTTTA GTTTAACTTC CTTCTCACAT TATTTTTTCC TAGAGTATCT CATTGCTCGG GTGGCATCCA AATTTTCCAG ACAAGGGCC-

**....|....| ....|....| ....|....| ....|....| ....|....| ....|....| ....|....| ....|....| ....|....| ....|....|**

**410 420 430 440 450 460 470 480 490 500**

**Morex**  ---------- ---------- ---------- ---------- ---------- ---------- ---------- ---------- ---------- ----------

**Harrington**  ATGCAATGGT TCTAGATGAT TGAAAAA--- -----ATATA TTTTTGTAGA TGATTGTATT AGTGTCGCAA ACATGCAACA CAATTTTTGT GCGAAAAGGA

**HA52**  ATGCAATGGT TCTAGATGAT TGAAAAA--- -----ATATA TTTTTGTAGA TGATTGTATT AGTGTCGCAA ACATGCAACA CAATTTTTGT GCGAAAAGGA

**z043**  ATGCAATGGT TCTAGATGAT TGAAAAA--- -----ATATA TTTTTGTAGA TGATTGTATT AGTGTCGCAA ACATGCAACA CAATTTTTGT GCGAAAAGGA

**L47**  ATGCAATGGT TCTAGATGAT TGAAAAA--- -----ATATA TTTTTGTAGA TGATTGTATT AGTGTCGCAA ACATGCAACA CAATTTTTGT GCGAAAAGGA

**Strider**  ---------- ---------- ---------- ---------- ---------- ---------- ---------- ---------- ---------- ----------

**W127**  ATGCAATGGT ----GATGAT TGGAAAACAA ATATTAAATA TTTTTGTAGA TGATTGTATT AGTGTCGCAA ACATGCAACA CAATTTTCGT ----------

**Adorra**  ATGCAATGGT TCTAGATGAT TGAAAAA--- -----ATATA TTTTTGTAGA TGATTGTATT AGTGTCGCAA ACATGCAACA CAATTTTCGT GCGAAAAGGA

**Haruna Nijo**  ATGCAATGGT TCTAGATGAT TGGAAAACAA ATATTAAATA TTTTTGTAGA TGATTGTATT AGTGTCGCAA ACATGCAACA CAATTTTCGT ----------

**L35**  ATGCAATGGT ----GATGAT TGGAAAACAA ATATTAAATA TTTTTGTAGA TGATTGTATT AGTGTCGCAA ACATGCAACA CAATTTTCGT ----------

**L48**  ATGCAATGGT TCTAGATGAT TGGAAAACAA ATATTAAATA TTTTTGTAGA TGATTGTATT AGTGTCGCAA ACATGCAACA CAATTTTCGT ----------

**L68**  ATGCAATGGT ----GATGAT TGGAAAACAA ATATTAAATA TTTTTGTAGA TGATTGTATT AGTGTCGCAA ACATGCAACA CAATTTTCGT ----------

**PI296897**  ATGCAATGGT TCTAGATGAT TGGAAAACAA ATATTAAATA TTTTTGTAGA TGATTGTATT AGTGTCGCAA ACATGCAACA CAATTTTCGT GCGAAAAGGA

**AB75**  ATGCAATGGT TCTAGATGAT TGGAAAACAA ATATTAAATA TTTTTGTAGA TGATTGTATT AGTGTCGCAA ACATGCAACA CAATTTTCGT ----------

**m279**  ATGCAATGGT TCTAGATGAT TGGAAAACAA ATATTAAATA TTTTTGTAGA TGATTGTATT AGTGTCGCAA ACATGCAACA CAATTTTCGT GCGAAAAGGA

**Legacy**  ATGCAATGGT TCTAGATGAT TGGAAAACAA ATATTAAATA TTTTTGTAGA TGATTGTATT AGTGTCGCAA ACATGCAACA CAATTTTCGT GCGAAAAGGA

**Orca**  ---------- ---------- ---------- ---------- ---------- ---------- ---------- ---------- ---------- ----------

**Tango**  ---------- ---------- ---------- ---------- ---------- ---------- ---------- ---------- ---------- ----------

**UC958**  ---------- ---------- ---------- ---------- ---------- ---------- ---------- ---------- ---------- ----------

**UC960**  ---------- ---------- ---------- ---------- ---------- ---------- ---------- ---------- ---------- ----------

**Steptoe**  ---------- ---------- ---------- ---------- ---------- ---------- ---------- ---------- ---------- ----------

**Stander**  ---------- ---------- ---------- ---------- ---------- ---------- ---------- ---------- ---------- ----------

**Ashqelon**  ATGCAATGGT TCTAGATGAT TGGAAAACAA ATATTAAATA TTTTTGTAGA TGATTGTATT AGTGTCGCAA ACATGCAACA CAATTTTCGT ----------

**L46**  ATGCAATGGT TCTAGATGAT TGGAAAACAA ATATTAAATA TTTTTGTAGA TGATTGTATT AGTGTCGCAA ACATGCAACA CAATTTTTGT GCGAAAAGGA

**....|....| ....|....| ....|....| ....|....| ....|....| ....|....| ....|....| ....|....| ....|....| ....|....|**

**510 520 530 540 550 560 570 580 590 600**

**Morex**  ---------- ---------- ---------- ---------- ---------- ---------- ---------- ---------- ---------- ----------

**Harrington**  GCAACGGTTC GGC----AAA AAACAAATTT AGGATGACAT TTTGGGGTAA CTTTTGGTGT TTAATTTGTT TTTTTGGCGC AAGCCAAAAT GTTT-AACCT

**HA52**  GCAACGGTTC GGC----AAA AAACAAATTT AGGATGACAT TTTGGGGTAA CTTTTGGTGT TTAATTTGTT TTTTTGGCGC AAGCAAAATT GTTTTAACCT

**z043**  GCAACGGTTC GGC---AAAA AAACAAATTT AGGATGATAT TTTGGGGTAA CTTTTGGTGT TTAATTTGTT TTTTTGGCGC AAGCCAAAAT GTTT-AACCT

**L47**  GCAACGGTTC GGC----AAA AAACAAATTT AGGATGACAT TTTGGGGTAA CTTTTGGTGT TTAATTTGTT TTTTTGGCGC AAGCCAAAAT GTTT-AACCT

**Strider**  ---------- ---------- ---------- ---------- ---------- ---------- ---------- ---------- ---------- ----------

**W127**  -CGGC----- ----AAAAAA AAACAAATTT AGGATGACAT TTTGGGGTAA CTTTTGGCGT TCAATTTGTT TTTTTGGCAC AAGCCAAAAT GTTT-AACCT

**Adorra**  GCGGCGGTTC GGC---AAAA AAACAAATTT AGGATGATAT TTTGGGGTAA CTTTTGGTGT TCAATTTGTT TTTTTGGCAC AAGCCAAAAT GTTT-AACCT

**Haruna Nijo**  -CGGC------ ---AAAAAA AAACAAATTT AGGATGACAT TTTGGGGTAA CTTTTGGCGT TCAATTTGTT TTTTTGGCAC AAGCCAAAAT GTTT-AACCT

**L35**  -CGGC------ ---AAAAAA AAACAAATTT AGGATGACAT TTTGGGGTAA CTTTTGGCGT TCAATTTGTT TTTTTGGCAC AAGCCAAAAT GTTT-AACCT

**L48**  -CGGC------ ---AAAAAA AAACAAATTT AGGATGACAT TTTGGGGTAA CTTTTGGCGT TCAATTTGTT TTTTTGGCAC AAGCCAAAAT GTTT-AACCT

**L68**  -CGGC------ --AAAAAAA AAACAAATTT AGGATGACAT TTTGGGGTAA CTTTTGGCGT TCAATTTGTT TTTTTGGCAC AAGCCAAAAT GTTTTAACCT

**PI296897**  GCGGCGGGTC GGC---AAAA AAAAAAATTT AGGATGACAT TTTGGGGTAA CTTTTGGTGT TCAATTTGTT TTTTTGGCAC AAGCCAAAAT GTTT-AACCT

**AB75**  -CGGC----- -----AAAAA AAACAAATTT AGGATGACAT TTTGGGGTAA CTTTTGGTGT TCAATTTGTT TTTTTGGCAC AAGCCAAAAT GTTT-AACCT

**m279**  GCGGCGGTTC GGC---AAAA AAAAAAATTT AGGATGACAT TTTGGGGTAA CTTTTGGTGT TCAATTTGTT TTTTTGGCAC AAGCCAAAAT GTTT-AACCT

**Legacy**  GCGGCGGTTC GGCAAAAAAA AAACAAATTT GGGATGACAT TTTGGGGTAA CTTTTGGTGT TCAATTTGTT TTTTTGGCAC AAGCCAAAAT GTTT-AACCT

**Orca**  ---------- ---------- ---------- ---------- ---------- ---------- ---------- ---------- ---------- ----------

**Tango**  ---------- ---------- ---------- ---------- ---------- ---------- ---------- ---------- ---------- ----------

**UC958**  ---------- ---------- ---------- ---------- ---------- ---------- ---------- ---------- ---------- ----------

**UC960**  ---------- ---------- ---------- ---------- ---------- ---------- ---------- ---------- ---------- ----------

**Steptoe**  ---------- ---------- ---------- ---------- ---------- ---------- ---------- ---------- ---------- ----------

**Stander**  ---------- ---------- ---------- ---------- ---------- ---------- ---------- ---------- ---------- ----------

**Ashqelon**  -CGG------ ---AAAAAAA AAACAAATTT AGGATGACAT TTTGGGGTAA CTTTTGGTGT TCAATTTGTT TTTTTGGCAC AAGCCAAAAT GTTT-AACCT

**L46**  GCAACGGTTC GGC----AAA AAACAAATTT AGGATGACAT TTTGGGGTAA CTTTTGGTGT TTAATTTGTT TTTTTGGCGC AAGCCAAAAT GTTT-AACCT

**....|....| ....|....| ....|....| ....|....| ....|....| ....|....| ....|....| ....|....| ....|....| ....|....|**

**610 620 630 640 650 660 670 680 690 700**

**Morex**  ---------- ---------- ---------- ---------- ---------- ---------- ---------- ---------- ---------- ----------

**Harrington**  TTTT-GCCTC AAAATTTGCA GGTAGCATTT GGATGTGACT AAGTACACAA ATAATTTTTG TCTTGAATTT TTTT-CATTT TGATTTTTTT TGGTCCCTGG

**HA52**  TTTTTGCCTC AAAATTTGCA GGTAGCATTT GGATGTGACT AAGTACACAA ATAATTTTTG TCTTGAATTT TTTT-CATTT TGATTTTTTT TGGTCCCTGG

**z043**  TTTT-GCCTC AAAATTTGCA GGTAGCATTT AGATATGACT AAGTACACAA ATAATTTTTG TCTTGAATTT TTTT-CATTT TGAATTTTTT TGGTCCCTGG

**L47**  TTTT-GCCTC AAAATTTGCA GGTAGCATTT GGATGTGACT AAGTACACAA ATAATTTTTG TCTTGAATTT TTTT-CATTT TGATTTTTTT TGGTCCCTGG

**Strider**  ---------- ---------- ---------- ---------- ---------- ---------- ---------- ---------- ---------- ----------

**W127**  TTTT-ACCTC AAAATTTGCA GGTAGCATTT AGATATGACT AAGTACACGA ATAAATTTTG TCTTGATTTT TTTT-CATTT TGAATTTTTT TGGCCCCCG-

**Adorra**  TTTT-ACCTC AAAATTTGCA GGTAGCATTT AGATATGACT AAGTACATAA ATAAATTTTG TCTTGATTTT TTTT-CATTT TGAATTTTTT TGGCCCCCG-

**Haruna Nijo**  TTTT-ACCTC AAAATTTGCA GGTAGCATTT AGATATGACT AAGTATATGA ATAAATTTTG TCTTGATTTT TTTT-CATTT TGAATTTTTT TGGCCCCCG-

**L35**  TTTT-ACCTC AAAATTTGCA GGTAGCATTT AGATATGACT AAGTACACGA ATAAATTTTG TCTTGATTTT TTTT-CATTT TGAATTTTTT TGGCCCCCG-

**L48**  TTTT-ACCTC AAAATTTGCA GGTAGCATTT AGATATGACT AAGTACACGA ATAAATTTTG TCTTGATTTT TTTT-CATTT TGAATTTTTT TGGCCCCCG-

**L68**  TTTT-ACCTC AAAATTTGCA GGTAGCATTT AGATATGACT AAGTACACGA ATAAATTTTG TCTTGATTTT TTTT-CATTT TGAATTTTTT TGGCCCCCG-

**PI296897**  TTTT-ACCTC AAAATTTGCA GGTAGCATTT AGATATGACT AAGTATATAA ATAAATTTTG TCTTGATTTT TTTT-CATTT TGAATTTTTT TGGCCCCCG-

**AB75**  TTTT-ACCTC AAAATTTGCA GGTAGCATTT AGATACGACT AAGTACATAA ATAAATTTTG TCTTGATTTT TTTT-CATTT TGAATTTTTT TGGCCCCCG-

**m279**  TTTT-ACCTC AAAATTTGCA GGTAGCATTT AGATATGACT AAGTATATAA ATAAATTTTG TCTTGATTTT TTTT-CATTT TGAATTTTTT TGGCCCCCG-

**Legacy**  TTTT-ACCTC AAAATTTGCA GGTAGCATTT AGATATAACT AAGTACATAA ATAAATTTTG TCTTGATTTT TTTTTCATTT TGAATTTTTT TGGCCCCCG-

**Orca**  ---------- ---------- ---------- ---------- ---------- ---------- ---------- ---------- ---------- ----------

**Tango**  ---------- ---------- ---------- ---------- ---------- ---------- ---------- ---------- ---------- ----------

**UC958**  ---------- ---------- ---------- ---------- ---------- ---------- ---------- ---------- ---------- ----------

**UC960**  ---------- ---------- ---------- ---------- ---------- ---------- ---------- ---------- ---------- ----------

**Steptoe**  ---------- ---------- ---------- ---------- ---------- ---------- ---------- ---------- ---------- ----------

**Stander**  ---------- ---------- ---------- ---------- ---------- ---------- ---------- ---------- ---------- ----------

**Ashqelon**  TTTT-ACCTC AAAATTTGCA GGTAGCATTT AGATACGACT AAGTACATAA ATAAATTTTG TCTTGATTTT TTTT-CATTT TGAATTTTTT TGGCCCCCG-

**L46**  TTTT-GCCTC AAAATTTGCA GGTAGCATTT GGATGTGACT AAGTACACAA ATAATTTTTG TCTTGAATTT TTTT-CATTT TGATTTTTTT TGGTCCCCGG

**....|....| ....|....| ....|....| ....|....| ....|....| ....|....| ....|....| ....|....| ....|....| ....|....|**

**710 720 730 740 750 760 770 780 790 800**

**Morex**  ---------- ---------- ---------- ---------- ---------- ---------- ---------- ---------- ---------- ----------

**Harrington**  AAGCATATTC TCCCTTGAGC CAAATTAACA TTC------- ---------- ---------- ---------- ---------- ---------- ----------

**HA52**  AAGCATATTC TCCCTTGAGC CAAATTAACA TTC------- ---------- ---------- ---------- ---------- ---------- ----------

**z043**  AAGCATATTC TCCCTTGAGC CAAATTAACA TTCCGGTCAT GATGTGGCTT GGATCCCAAG TTAGTTATAC AGATAAGGAT ATATCTTACC TCAACCGAAT

**L47**  AAGCATATTC TCCCTTGAGC CAAATTAACA TTC------- ---------- ---------- ---------- ---------- ---------- ----------

**Strider**  ---------- ---------- ---------- ---------- ---------- ---------- ---------- ---------- ---------- ----------

**W127**  AAGCATATTC TTCGGGGAGC CAAATTGACA TTCCGGTCAT GATGTGGCTT GGATCCCAAG TTAGCTATAC AGATAAGGAT ATATCTTACC TCAACCGAAT

**Adorra**  AAGCATATTC TTCCGGGAGC CAAATTGACA TTCCGGTCAT GATGTGGCTT GGATCCCAAG TTAGTTATAC AGATAAGGAT ATATCTTACC TCAACCGAAT

**Haruna Nijo**  AAGCATATTC TTCCGGGAGC CAAATTGACA TTCCGGTCAT GATGTGGCTT GGATCCCAAG TTAGCTATAC AGATAAGGAT ATATCTTACC TCAACCGAAT

**L35**  AAGCATATTC TTCGGGGAGC CAAATTGACA TTCCGGTCAT GATGTGGCTT GGATCCCAAG TTAGCTATAC AGATAAGGAT ATATCTTACC TCAACCGAAT

**L48**  AAGCATATTC TTCGGGGAGC CAAATTGACA TTCCGGTCAT GATGTGGCTT GGATCCCAAG TTAGCTATAC AGATAAGGAT ATATCTTACC TCAACCGAAT

**L68**  AAGCATATTC TTCGGGGAGC CAAATTGACA TTCCGGTCAT GATGTGGCTT GGATCCCAAG TTAGCTATAC AGATAAGGAT ATATCTTACC TCAACCGAAT

**PI296897**  AAGCATATTC TTCCGGGAGC CAAATTGACA TTCCGGTCAT GATGTGGCTT GGATCCCAAG TTAGTCATAC AGATAAGGAT ATATCTTACC TCAACCGAAT

**AB75**  AAGCATATTC TTCCGGGAGC CAAATTGACA TTTCGGTCAT GATGTGGCTT GGATCCCAAG TTAGTCATAC AGATAAGGAT ATATCTTACC TCAACCGAAT

**m279**  AAGCATATTC TTCCGGGAGC CAAATTGACA TTCCGGTCAT GATGTGGCTT GGATCCCAAG TTAGCTATAC AGATAAGGAT ATATCTTACC TCAACCGAAT

**Legacy**  AAGCATATTC TTCCGGGAGC CAAATTGACA TTCCGGTCAT GATGTGGCTT GGATCCCAAG TTAGTTATAC AGATAAGGAT ATATCTTACC TCAACCGAAT

**Orca**  ---------- ---------- ---------- ---------- ---------- ---------- ---------- ---------- ---------- ----------

**Tango**  ---------- ---------- ---------- ---------- ---------- ---------- ---------- ---------- ---------- ----------

**UC958**  ---------- ---------- ---------- ---------- ---------- ---------- ---------- ---------- ---------- ----------

**UC960**  ---------- ---------- ---------- ---------- ---------- ---------- ---------- ---------- ---------- ----------

**Steptoe**  ---------- ---------- ---------- ---------- ---------- ---------- ---------- ---------- ---------- ----------

**Stander**  ---------- ---------- ---------- ---------- ---------- ---------- ---------- ---------- ---------- ----------

**Ashqelon**  AAGCATATTC TTCCGGGAGC CAAATTGACA TTCCGGTCAT GATGTGGCTT GGATCCCAAG TTAGTCATAC AGATAAGGAT ATATCTTACC TCAACCGAAT

**L46**  AAGCATATTC TCCCTTGAGC CAAATTAACT TTCCGGTCAT GATGTGGCTT GGATCCCAAG TTAGCTATAC AGATAAGGAT ATATCTTACC TCAACCGAAT

**....|....| ....|....| ....|....| ....|....| ....|....| ....|....| ....|....| ....|....| ....|....| ....|....|**

**810 820 830 840 850 860 870 880 890 900**

**Morex**  ---------- ---------- ---------- ---------- ---------- ---------- ---------- ---------- ---------- ----------

**Harrington**  ---------- ---------- -----ATGCA TTAGTGTCCG TCCTAGACTC CTCTAGAAGG CAATGGTTTA CACACCATTG TGGTTTGTAC AAATTCCAAC

**HA52**  ---------- ---------- -----ATGCA TTAGTGTCCG TCCTAGACTC CTCTAGAAGG CAATGGTTTA CACACCATTG TGGTTTGTAC AAATTCCAAC

**z043**  CCAGGTTACA CCAAGCTTAA CACTCATGCA TTAGTGTCCA TCCTAGACTC CTCTAGAAGG CAATGGTTTA CACACCATTG TGGTTTGTAC AAATTCCAAC

**L47**  ---------- ---------- -----ATGCA TTAGTGTCCG TCCTAGACTC CTCTAGAAGG CAATGGTTTA CACACCATTG TGGTTTGTAC AAATTCCAAC

**Strider**  ---------- ---------- ---------- ---------- ---------- ---------- ---------- ---------- ---------- ----------

**W127**  CTAGGTTACA ACAAGCTTAA CACTCATGCA TTAGTGTCCA TCCTAGACTC CTCTAGAAGG CAATGGTTTA CACACCATTG TGGTTTGTAC AAATTCCAAC

**Adorra**  CTAGGTTACA ACAAGCTTAA CACTCATGCA TTAGTGTCCA TCCTAGACTC CTCTAGAAGG CAATGGTTTA CACACCATTG TGGTTTGTAC AAATTCCAAC

**Haruna Nijo**  CTAGGTTACA ACAAGCTTAA CACTCATGCA TTAGTGTCCA TCCTAGACTC CTCTAGAAGG CAATGGTTTA CACACCATTG TGGTTTGTAC AAATTCCAAC

**L35**  CTAGGTTACA ACAAGCTTAA CACTCATGCA TTAGTGTCCA TCCTAGACTC CTCTAGAAGG CAATGGTTTA CACACCATTG TGGTTTGTAC AAATTCCAAC

**L48**  CTAGGTTACA ACAAGCTTAA CACTCATGCA TTAGTGTCCA TCCTAGACTC CTCTAGAAGG CAATGGTTTA CACACCATTG TGGTTTGTAC AAATTCCAAC

**L68**  CTAGGTTACA ACAAGCTTAA CACTCATGCG TTAGTGTCCA TCCTAGACTC CTCTAGAAGG CAATGGTTTA CACACCATTG TGGTTTGTAC AAATTCCAAC

**PI296897**  CTAGGTTACA ACAAGCTTAA CACTCATGCA TTAGTGTCCA TCCTAGACTC CTCTAGAAGG CAATGGTTTA CACACCATTG TGGTTTGTAC AAATTCCAAC

**AB75**  CTAGGTTACA ACAAGCTTAA CACTCATGCA TTAGTGTCCA TCCTAGACTC CTCTAGAAGG CAATGGTTTA CACACCATTG TGGTTTGTAC AAATTCCAAC

**m279**  CTAGGTTACA ACAAGCTTAA CACTCATGCA TTAGTGTCCA TCCTAGACTC CTCTAGAAGG CAATGGTTTA CACACCATTG TGGTTTGTAC AAATTCCAAC

**Legacy**  CTAGGTTACA CCAAGCTTAA CACTCATGCA TTAGTGTCCA TCCTAGACTC CTCTAGAAGG CAATGGTTTA CACACCATTG TGGTTTGTAC AAATTCCAAC

**Orca**  ---------- ---------- ---------- ---------- ---------- ---------- ---------- ---------- ---------- ----------

**Tango**  ---------- ---------- ---------- ---------- ---------- ---------- ---------- ---------- ---------- ----------

**UC958**  ---------- ---------- ---------- ---------- ---------- ---------- ---------- ---------- ---------- ----------

**UC960**  ---------- ---------- ---------- ---------- ---------- ---------- ---------- ---------- ---------- ----------

**Steptoe**  ---------- ---------- ---------- ---------- ---------- ---------- ---------- ---------- ---------- ----------

**Stander**  ---------- ---------- ---------- ---------- ---------- ---------- ---------- ---------- ---------- ----------

**Ashqelon**  CTAGGTTACA ACAAGCTTAA CACTCATGCA TTAGTGTCCA TCCTAGACTC CTCTAGAAGG CAATGGTTTA CACACCATTG TGGTTTGTAC AAATTCCAAC

**L46**  CTAGGTTACA ACAAGCTTAA CATTCATGCA TTAGTGTCCG TCCTAGACTC CTCTAGAAGG CAATGGTTTA CACACCATTG TGGTTTGTAC AAATTCCAAC

**....|....| ....|....| ....|....| ....|....| ....|....| ....|....| ....|....| ....|....| ....|....| ....|....|**

**910 920 930 940 950 960 970 980 990 1000**

**Morex**  ---------- ---------- ---------- ---------- ---------- ---------- ---------- ---------- ---------- ----------

**Harrington**  ACTCTTCCTC AATCATAACT TTATGAAGTC GAGATTGATC TTAAAGTTCT GATGTTATCT GTTAAAGGAA TAGCTTGGGA AACACATGTA CAACTTAAGT

**HA52**  ACTCTTCCTC AATCATAACT TTATGAAGTC GAGATTGATC TTAAAGTTCT GATGTTATCT GTTAAAGGAA TAGCTTGGGA AACACATGTA CAACTTAAGT

**z043**  ACTCTTCCTC AATCATAACT TTATGAAGTC GAGATTGATC TTAAAGTTCT GATGTTATCT GTTAAAGGAA TAGCTTGGGA AACACATGTA CAACTTAAGT

**L47**  ACTCTTCCTC AATCATAACT TTATGAAGTC GAGATTGATC TTAAAGTTCT GATGTTATCT GTTAAAGGAA TAGCTTGGGA AACACATGTA CAACGTAAGT

**Strider**  ---------- ---------- ---------- ---------- ---------- ---------- ---------- ---------- ---------- ----------

**W127**  ACTCTTCCTC AATCATAACT TTATGAAGTC GAGATTGATC TTAAAGTTCT GATGTTATCT GTTAAA-GAA TAGCTTGGGA AACACATGTA CAACGTAAGT

**Adorra**  ACTCTTCCTC AATCATAACT TTATGAAGTC GAGATTGATC TTAAAGTTCT GATGTTATCT GTTAAA-GAA TAGCTTGGGA AACATATGTA CAACTTAAGT

**Haruna Nijo**  ACTCTTCCTC AATCATAACT TTATGAAGTC GAGATTGATC TTAAAGTTCT GATGTTATCT GTTAAA-GAA TAGCTTGGGA AACACATGTA CAACTTAAGT

**L35**  ACTCTTCCTC AATCATAACT TTATGAAGTC GAGATTGATC TTAAAGTTCT GATGTTATCT GTTAAA-GAA TAGCTTGGGA AACACATGTA CAACGTAAGT

**L48**  ACTCTTCCTC AATCATAACT TTATGAAGTC GAGATTGATC TTAAAGTTCT GATGTTATCT GTTAAA-GAA TAGCTTGGGA AACACATGTA CAACGTAAGT

**L68**  ACTCTTCCTC AATCATAACT TTATGAAGTC GAGATTGATC TTAAAGTTCT GATGTTATCT GTTAAA-GAA TAGCTTGGGA AACACATGTA CAACGTAAGT

**PI296897**  ACTCTTCCTC AATCATAACT TTATGAAGTC GAGATTGATC TTAAAGTTCT GATGTTATCT GTTAAA-GAA TAGCTTGGGA AACACATGTA CAACTTAAGT

**AB75**  ACTCTTCCTC AATCATAACT TTATGAAGTC GAGATTGATC TTAAAGTTCT GATGTTATCT GTTAAA-GAA TAGCTTGGGA AACACATGTA CAACTTAAGT

**m279**  ACTCTTCCTC AATCATAACT TTATGAAGTC GAGATTAATC TTAAAGTTCT GATGTTATCT GTTAAA-GAA TAGCTTGGGA AACACATGTA CAACGTAAGT

**Legacy**  ACTCTTCCTC AATCATAACT TTATGAAGTC GAGATTGATC TTAAAGTTCT GATGTTATCT GTTAAAGGAA TAGCTTGGGA AACACATGTA CAACTTAAGT

**Orca**  ---------- ---------- ---------- ---------- ---------- ---------- ---------- ---------- ---------- ----------

**Tango**  ---------- ---------- ---------- ---------- ---------- ---------- ---------- ---------- ---------- ----------

**UC958**  ---------- ---------- ---------- ---------- ---------- ---------- ---------- ---------- ---------- ----------

**UC960**  ---------- ---------- ---------- ---------- ---------- ---------- ---------- ---------- ---------- ----------

**Steptoe**  ---------- ---------- ---------- ---------- ---------- ---------- ---------- ---------- ---------- ----------

**Stander**  ---------- ---------- ---------- ---------- ---------- ---------- ---------- ---------- ---------- ----------

**Ashqelon**  ACTCTTCCTC AATCATAACT TTATGAAGTC GAGATTGATC TTAAAGTTCT GATGTTATCT GTTAAA-GAA TAGCTTGGGA AACACATGTA CAACTTAAGT

**L46**  ACTCTTCCTC AATCATAACT TTATGAAGTC GAGATTGATC TTAAAGTTCT GATGTTATCT GTTAAAAGAA TAGCTTGGGA AACACATGTA CAACTTAAGT

**....|....| ....|....| ....|....| ....|....| ....|....| ....|....| ....|....| ....|....| ....|....| ....|....|**

**1010 1020 1030 1040 1050 1060 1070 1080 1090 1100**

**Morex**  ---------- ---------- ---------- ---------- ---------- ---------- ---------- ---------- ---------- ----------

**Harrington**  CAGGATGTGT GCGTCTTCAC TTCGTATAGG GTGCCGTTTG GTTGAGAGTT GAGACGTGTA TTGGATCCCA CGTTAGCTAT AATATAGATA ATGATCCCAT

**HA52**  CAGGATGTGT GCGTCTTCAC TTCGTATATG GTGCCGTTTG GTTGAGAGTT GAGACGTGTA TTGGATCCCA CGTTAGCTAT AATATAGATA ATGATCCCAT

**z043**  CAGGATGTGT GCGTCTTCAC TTCGTATAGG GTGCCGTTTG GTTGAGAGTT GAGACGTGTA TTGGGTCCCA CGTTAGCTAT AATATAGATA ATGATCCCAT

**L47**  CAGGATGTGT GCGTCTTCAC TTCGTATAGG GTGCCGTTTG GTTGAGAGTT GAGACGTGTA TTGGATCCCA CGTTAGCTAT AATATAGATA ATGATCCCAT

**Strider**  ---------- ---------- ---------- ---------- ---------- ---------- ---------- ---------- ---------- ----------

**W127**  CAGGATGGGT GCGTCTTCAC TTCGTATAGG GTGCCGTTTG GTTGAGAGTT GAGACGTGGA TTGGATCCCA CGTTAGCTAT AATACAGATA ATGATCTCAT

**Adorra**  CAGGATGTGT GCGTCTTCAC TTCGTATAGG GTGCCGTTTG GTTGAGAGTT GAGACGTGGA TTGGATCCCA CGTTAGCTAT AATATAGATA ATGATCCCAT

**Haruna Nijo**  CAGGATGTGT GCGTCTTCAC TTCGTATAGG GTGCCGTTTG GTTGAGAGTT GAGACGTGGA TTGGATCCCA CGTTAGCTAT AATATAGATA ATGATCCCAT

**L35**  CAGGATGGGT GCGTCTTCAC TTCGTATAGG GTGCCGTTTG GTTGAGAGTT GAGACGTGGA TTGGATCCCA CGTTAGCTGT AATACAGATA ATGATCTCAT

**L48**  CAGGATGGGT GCGTCTTCAC TTCGTATAGG GTGCCGTTTG GTTGAGAGTT GAGACGTGGA TTGGATCCCA CGTTAGCTAT AATACAGATA ATGATCTCAT

**L68**  CAGGATGGGT GCGTCTTCAC TTCGTATAGG GTGCCGTTTG GTTGAGAGTT GAGACGTGGA TTGGATCCCA CGTTAGCTAT AATACAGATA ATGATCTCAT

**PI296897**  CAGGATGTGT GCGTCTTCAC TTCGTATAGG GTGCCGTTTG GTTGAGAGTT GAGACGTGGA TTGGATCCCA CGTTAGCTAT AATATAGATA ATGATCCCAT

**AB75**  CAGGATGTGT GCGTCTTCAC TTCGTATAGG GTGCCGTTTG GTTGAGAGTT GAGACGTGGA TTGGATCCCA CGTTAGCTAT AATATAGATA ATGATCCCAT

**m279**  CAGGATGGGT GCGTCTTCAC TTCGTATAGG GTGCCGTTTG GTTGAGAGTT GAGACGTGGA TTGGATCCCA CGTTAGCTAT AATACAGATA ATGATCTCAT

**Legacy**  CAGGATGTGT GCGTCTTCAC TTCGTATAGG GTGCCGTTTG GTTGAGAGTT GAGACGTGTA TTGGGTCCCA CGTTAGCTAT AATATAGATA ATGATCCCAT

**Orca**  ---------- ---------- ---------- ---------- ---------- ---------- ---------- ---------- ---------- ----------

**Tango**  ---------- ---------- ---------- ---------- ---------- ---------- ---------- ---------- ---------- ----------

**UC958**  ---------- ---------- ---------- ---------- ---------- ---------- ---------- ---------- ---------- ----------

**UC960**  ---------- ---------- ---------- ---------- ---------- ---------- ---------- ---------- ---------- ----------

**Steptoe**  ---------- ---------- ---------- ---------- ---------- ---------- ---------- ---------- ---------- ----------

**Stander**  ---------- ---------- ---------- ---------- ---------- ---------- ---------- ---------- ---------- ----------

**Ashqelon**  CAGGATGTGT GCGTCTTCAC TTCGTATAGG GTGCCGTTTG GTTGAGAGTT GAGACGTGGA TTGGATCCCA CGTTAGCTAT AATATAGATA ATGATCCCAT

**L46**  CAGGATGTGT GCGTCTTCAC TTCGTATAGG GTGCCGTTTG GTTGAGAGTT GAGACGTGTA TTGGATCCCA CGTTAGCTAT AATATAGATA ATGATCCCAT

**....|....| ....|....| ....|....| ....|....| ....|....| ....|....| ....|....| ....|....| ....|....| ....|....|**

**1110 1120 1130 1140 1150 1160 1170 1180 1190 1200**

**Morex**  ---------- ---------- ---------- ---------- ---------- ---------- ---------- ---------- ---------- ----------

**Harrington**  CTCTCTCCCA ATCAGACCTC AATCCTTGAA GTTTGGCTGT GTGTGGTAAA GAAAACCAAA ATTAGTTCAC AAAGCGCCCT TTCTGAGTGG ACAAGCCTAT

**HA52**  CTCTCTCCCA ATCAGACCTC AATCCTTGAA GTTTGGCTGT GTGTGGTAAA GAAAACCAAA ATTAGTTCAC AAAGCGCCCT TTCTGAGTGG ACAAGCCTAT

**z043**  CTCTCTCCCA ATCAGACCTC AATCCTTGAA GTTTGGCTGT GTGTGGTAAA GAAAACCAAA ATTAGTTCAC AAAGCGCCCT TTCTGAGTGG ACAAGCCTAT

**L47**  CTCTCTCCCA ATCAGACCTC AATCCTTGAA GTTTGGCTGT GTGTGGTAAA GAAAACCAAA ATTAGTTCAC AAAGCGCCCT TTCTGAGTGG ACAAGCCTAT

**Strider**  ---------- ---------- ---------- ---------- ---------- ---------- ---------- ---------- ---------- ----------

**W127**  CTCTCTCCCA ATCAGACCTC AATCCTTGAA GTTTGGCTGT GTGTGGTAAA GAAAACCAAA ATTAGTTCAC AAAGCGCCCT TTCTGAGTGG ACAATCCTAT

**Adorra**  CTCTCTCCCA ATCAGACCTC AATCCTTGAA GTTTGGCTGT GTGTGGTAAA GAAAACCAAA ATTAGTTCAC AAAGCGCCCT TTCTGAGTGG ACAAGCCTAT

**Haruna Nijo**  CTCTCTCCCA ATCAGACCTC AATCCTTGAA GTTTGGCTGT GTGTGGTAAA GAAAACCAAA ATTAGTTCAC AAAGCGCCCT TTCTGAGTGG ACAATCCTAT

**L35**  CTCTCTCCCA ATCAGACCTC AATCCTTGAA GTTTGGCTGT GTGTGGTAAA GAAAACCAAA ATTAGTTCAC AAAGCGCCCT TTCTGAGTGG ACAATCCTAT

**L48**  CTCTCTCCCA ATCAGACCTC AATCCTTGAA GTTTGGCTGT GTGTGGTAAA GAAAACCAAA ATTAGTTCAC AAAGCGCCCT TTCTGAGTGG ACAATCCTAT

**L68**  CTCTCTCCCA ATCAGACCTC AATCCTTGAA GTTTGGCTGT GTGTGGTAAA GAAAACCAAA ATTAGTTCAC AAAGCGCCCT TTCTGAGTGG ACAATCCTAT

**PI296897**  CTCTCTCCCA ATCAGACCTC AATCCTTGAA GTTTGGCTGT GTGTGGTAAA GAAAACCAAA ATTAGTTCAC AAAGCGCCCT TTCTGAGTGG ACAATCCTAT

**AB75**  CTCTCTCCCA ATCAGACCTC AATCCTTGAA GTTTGGCTGT GTGTGGTAAA GAAAACCAAA ATTAGTTCAC AAAGCGCCCT TTCTGAGTGG ACAATCCTAT

**m279**  CTCTCTCCCA ATCAGACCTC AATCCTTGAA GTTTGGCTGT GTGTGGTAAA GAAAACCAAA ATTAGTTCAC AAAGCGCCCT TTCTGAGTGG ACAATCCTAT

**Legacy**  CTCTCTCCCA ATCAGACCTC AATCCTTGAA GTTTGGCTGT GTGTGGTAAA GAAAACCAAA ATTAGTTCAC AAAGCGCCCT TTCTGAGTGG ACAAGCCTAT

**Orca**  ---------- ---------- ---------- ---------- ---------- ---------- ---------- ---------- ---------- ----------

**Tango**  ---------- ---------- ---------- ---------- ---------- ---------- ---------- ---------- ---------- ----------

**UC958**  ---------- ---------- ---------- ---------- ---------- ---------- ---------- ---------- ---------- ----------

**UC960**  ---------- ---------- ---------- ---------- ---------- ---------- ---------- ---------- ---------- ----------

**Steptoe**  ---------- ---------- ---------- ---------- ---------- ---------- ---------- ---------- ---------- ----------

**Stander**  ---------- ---------- ---------- ---------- ---------- ---------- ---------- ---------- ---------- ----------

**Ashqelon**  CTCTCTCCCA ATCAGACCTC AATCCTTGAA GTTTGGCTGT GTGTGGTAAA GAAAACCAAA ATTAGTTCAC AAAGCGCCCT TTCTGAGTGG ACAATCCTAT

**L46**  CTCTCTCCCA ATCAGACCTC AATCCTTGAA GTTTGGCTGT GTGTGGTAAA GAAAACCAAA ATTAGTTCAC AAAGCGCCCT TTCTGAGTGG ACAAGCCTAT

**....|....| ....|....| ....|....| ....|....| ....|....| ....|....| ....|....| ....|....| ....|....| ....|....|**

**1210 1220 1230 1240 1250 1260 1270 1280 1290 1300**

**Morex**  ---------- ---------- ---------- ---------- ---------- ---------- ---------- ---------- ---------- ----------

**Harrington**  TT-CTGACAT ATCTG-ATTG GAAAGCTAGG TTCGCCGTTG GCCTCACATC TATGGATACA TCTTTTTCAT AGTATAAATA GACCCTTTTA TTAAGCTCCC

**HA52**  TT-CTGACAT ATCTGGATTG GAAAGCTAGG TTCGC-GTTG GCCTCACATC TATGGATACA TCTTTTTCAT AGTATAAATA GACCCTTTTA TTAAGCTCCC

**z043**  TT-CTGACAT ATCTG-ATTG GAAAGCTAGG TTCGCCGTTG GCCTCACATC TATGGATACA TCTTTTTCAT AGTATAAATA GACCCTTTTA TTAAGCTCCC

**L47**  TT-CTGACAT ATCTG-ATTG GAAAGCTAGG TTCGCCGTTG GCCTCACATC TATGGATACA TCTTTTTCAT AGTATAAATA GACCCTTTTA TTAAGCTCCC

**Strider**  ---------- ---------- ---------- ---------- ---------- ---------- ---------- ---------- ---------- ----------

**W127**  TT-CTGACAT ATCTG-ATTG GAAAGCTAGG TTCGCCGTTG GCCTCACATC TATGGATACA TCTTTTTCAT AGTATAAATA GACCCTTTTA TTAAGCTCCC

**Adorra**  TTTCTGACAT ATCTG-ATTG GAAAGCTAGG TTCGCCGTTG GCCTCACATC TATGGATACA TCTTTTTCAT AGTATAAATA GACCCTTTTA TTAAGCTCCC

**Haruna Nijo**  TT-CTGACAT ATCTG-ATTG GAAAGCTAGG TTCGCCGTTG GCCTCACATC TATGGATACA TCTTTTTCAT AGTATAAATA GACCCTTTTA TTAAGCTCCC

**L35**  TT-CTGACAT ATCTG-ATTG GAAAGCTAGG TTCGCCGTTG GCCTCACATC TATGGATACA TCTTTTTCAT AGTATAAATA GACCCTTTTA TTAAGCTCCC

**L48**  TT-CTGACAT ATCTG-ATTG GAAAGCTAGG TTCGCCGTTG GCCTCACATC TATGGATACA TCTTTTTCAT AGTATAAATA GACCCTTTTA TTAAGCTCCC

**L68**  TT-CTGACAT ATCTG-ATTG GAAAGCTAGG TTCGCCGTTG GCCTCACATC TATGGATACA TCTTTTTCAT AGTATAAATA GACCCTTTTA TTAAGCTCCC

**PI296897**  TT-CTGACAT ATCTG-ATTG GAAAGCTAGG TTCGCCGTTG GCCTCACATC TATGGATACA TCTTTTTCAT AGTATAAATA GACCCTTTTA TTAAGCTCCC

**AB75**  TT-CTGACAT ATCTG-ATTG GAAAGCTAGG TTCGCCGTTG GCCTCACATC TATGGATACA TCTTTTTCAT AGTATAAATA GACCCTTTTA TTAAGCTCCC

**m279**  TT-CTGACAT ATCTG-ATTG GAAAGCTAGG TTCGCCGTTG GCCTCACATC TATGGATACA TCTTTTTCAT AGTATAAATA GACCCTTTTA TTAAGCTCCC

**Legacy**  TT-CTGACAT ATCTG-ATTG GAAAGCTAGG TTCGCCGTTG GCCTCACATC TATGGATACA TCTTTTTCAT AGTATAAATA GACCCTTTTA TTAAGCTCCC

**Orca**  ---------- ---------- ---------- ---------- ---------- ---------- ---------- ---------- ---------- ----------

**Tango**  ---------- ---------- ---------- ---------- ---------- ---------- ---------- ---------- ---------- ----------

**UC958**  ---------- ---------- ---------- ---------- ---------- ---------- ---------- ---------- ---------- ----------

**UC960**  ---------- ---------- ---------- ---------- ---------- ---------- ---------- ---------- ---------- ----------

**Steptoe**  ---------- ---------- ---------- ---------- ---------- ---------- ---------- ---------- ---------- ----------

**Stander**  ---------- ---------- ---------- ---------- ---------- ---------- ---------- ---------- ---------- ----------

**Ashqelon**  TT-CTGACAT ATCTG-ATTG GAAAGCTAGG TTCGCCGTTG GCCTCACATC TATGGATACA TCTTTTTCAT AGTATAAATA GACCCTTTTA TTAAGCTCCC

**L46**  TT-TTGACAT ATCTG-ATTG GAAAGCTAGG TTCGCCGTTG GCCTCACATC TATGGATACA TCTTTTTCAT AGTATAAATA GACCCTTTTA TTAAGCTCCC

**....|....| ....|....| ....|....| ....|....| ....|....| ....|....| ....|....| ....|....| ....|....| ....|....|**

**1310 1320 1330 1340 1350 1360 1370 1380 1390 1400**

**Morex**  ---------- ---------- ---------T AGAGCATCAT CCATAGCCAG CATCCACAAT GGAGGTGAAC GTGAAAGGCA ACTATGTCCA AGTCTACGTC

**Harrington**  TGCCATATCC AACAAACCAT TTGAAGTTGT AGAGCATCAT CCATAGCCAG CATCCACAAT GGAGGTGAAC GTGAAAGGCA ACTATGTCCA AGTCTACGTC

**HA52**  TGCCATATCC AACAAACCAT TTGAAGTTGT AGAGCATCAT CCATAGCCAG CATCCACAAT GGAGGTGAAC GTGAAAGGCA ACTATGTCCA AGTCTACGTC

**z043**  AGCCATATCC AACAAACCAT TTGAAGTTGT AGAGCATCAT CCATAGCCAG CATCCACAAT GGAGGTGAAC GTGAAAGGCA ACTATGTCCA AGTCTACGTC

**L47**  TGCCATATCC AACAAACCAT TTGAAGTTGT AGAGCATCAT CCATAGCCAG CATCCACAAT GGAGGTGAAC GTGAAAGGCA ACTATGTCCA AGTCTACGTC

**Strider**  ---------- ---------- ---------T AGAGCATCAT CCATAGCCAG CATCCACAAT GGAGGTGAAC GTGAAAGGCA ACTATGTCCA AGTCTACGTC

**W127**  TGCCATATCC AACAAACCAT TTGAAGTTGT AGAGCATCAT CCATAGCCAG CATCCACAAT GGAGGTGAAC GTGAAAGGCA ACTATGTCCA AGTCTACGTC

**Adorra**  TGCCATATCC AACAAACCAT TTGAAGTTGT AGAGCATCAT CCATAGCCAG CATCCACAAT GGAGGTGAAC GTGAAAGGCA ACTATGTCCA AGTCTACGTC

**Haruna Nijo**  TGCCATATCC AACAAACCAT TTGAAGTTGT AGAGCATCAT CCATAGCCAG CATCCACAAT GGAGGTGAAC GTGAAAGGCA ACTATGTCCA AGTCTACGTC

**L35**  TGCCATATCC AACAAACCAT TTGAAGTTGT AGAGCATCAT CCATAGCCAG CATCCACAAT GGAGGTGAAC GTGAAAGGCA ACTATGTCCA AGTCTACGTC

**L48**  TGCCATATCC AACAAACCAT TTGAAGTTGT AGAGCATCAT CCATAGCCAG CATCCACAAT GGAGGTGAAC GTGAAAGGCA ACTATGTCCA AGTCTACGTC

**L68**  TGCCATATCC AACAAACCAT TTGAAGTTGT AGAGCATCAT CCATAGCCAG CATCCACAAT GGAGGTGAAC GTGAAAGGCA ACTATGTCCA AGTCTACGTC

**PI296897**  TGCCATATCC AACAAACCAT TTGAAGTTGT AGAGCATCAT CCATAGCCAG CATCCACAAT GGAGGTGAAC GTGAAAGGCA ACTATGTCCA AGTCTACGTC

**AB75**  TGCCATATCC AACAAACCAT TTGAAGTTGT AGAGCATCAT CCATAGCCAG CATCCACAAT GGAGGTGAAC GTGAAAGGCA ACTATGTCCA AGTCTACGTC

**m279**  TGCCATATCC AACAAACCAT TTGAAGTTGT AGAGCATCAT CCATAGCCAG CATCCACAAT GGAGGTGAAC GTGAAAGGCA ACTATGTCCA AGTCTACGTC

**Legacy**  TGCCATATCC AACAAACCAT TTGAAGTTGT AGAGCATCAT CCATAGCCAG CATCCACAAT GGAGGTGAAC GTGAAAGGCA ACTATGTCCA AGTCTACGTC

**Orca**  ---------- ---------- ---------- -----ATCAT CCATAGCCAG CATCCACAAT GGAGGTGAAC GTGAAAGGCA ACTATGTCCA AGTCTACGTC

**Tango**  ---------- ---------- ---------- -----ATCAT CCATAGCCAG CATCCACAAT GGAGGTGAAC GTGAAAGGCA ACTATGTCCA AGTCTACGTC

**UC958**  ---------- ---------- ---------- -----ATCAT CCATAGCCAG CATCCACAAT GGAGGTGAAC GTGAAAGGCA ACTATGTCCA AGTCTACGTC

**UC960**  ---------- ---------- ---------- -----ATCAT CCATAGCCAG CATCCACAAT GGAGGTGAAC GTGAAAGGCA ACTATGTCCA AGTCTACGTC

**Steptoe**  ---------- ---------- ---------- -----ATCAT CCATAGCCAG CATCCACAAT GGAGGTGAAC GTGAAAGGCA ACTATGTCCA AGTCTACGTC

**Stander**  ---------- ---------- ---------- -----ATCAT CCATAGCCAG CATCCACAAT GGAGGTGAAC GTGAAAGGCA ACTATGTCCA AGTCTACGTC

**Ashqelon**  TGCCATATCC AACAAACCAT TTGAAGTTGT AGAGCATCAT CCATAGCCAG CATCCACAAT GGAGGTGAAC GTGAAAGGCA ACTATGTCCA AGTCTACGTC

**L46**  TGCCATATCC AACAAACCAT TTGAAGTTGT AGAGCATCAT CCATAGCCAG CATCCACAAT GGAGGTGAAC GTGAAAGGCA ACTATGTCCA AGTCTACGTC

**....|....| ....|....| ....|....| ....|....| ....|....| ....|....| ....|....| ....|....| ....|....| ....|....|**

**1410 1420 1430 1440 1450 1460 1470 1480 1490 1500**

**Morex**  ATGCTCCCTG TAAGCTCCCA TCCATTCAGA CCAATCGCTG AGAACCACAC ACTAAAACTA TTTCAAGGAT CTAGTGCACA CATATACATT ATTGTTGTAC

**Harrington**  ATGCTCCCTG TAAGCTCCCA TCCATTCAGA CCAATCGCTG AGAACCACAC ACTAAAACTA TTTCAAGGAT CTAGTGCACA CATATACATT ATTGTTGTAC

**HA52**  ATGCTCCCTG TAAGCTCCCA TCCATTCAGA CCAATCGCTG AGAACCACAC ACTAAAACTA TTTCAAGGAT CTAGTGCACA CATATACATT ATTGTTGTAC

**z043**  ATGCTCCCTG TAAGCTCCCA TCCATTCAGA CCAATCGCTG AGAACCACAC ACTAAAACTA TTTCAAGGAT CTAGTGCACA CATATACATT ATTGTTGTAC

**L47**  ATGCTCCCTG TAAGCTCCCA TCCATTCAGA CCAATCGCTG AGAACCACAC ACTAAAACTA TTTCAAGGAT CTAGTGCACA CATATACATT ATTGTTGTAC

**Strider**  ATGCTCCCTG TAAGCTCC-A TCCATTCAGA CCAATCGCTG AGAACCACAC ACTAAAACTA TTTCAAGGAT CTAGTGCACA CATATACATT ATTGTTGTAC

**W127**  ATGCTCCCTG TAAGCTCC-A TCCATTCAGA CCAATCGCTG AGAACCACAC ACTAAAACTA TTTCAAGGAT CTAGTGCACA CATATACATT ATTGTTGTAC

**Adorra**  ATGCTCCCTG TAAGCTCC-A TCCATTCAGA CCAATCGCTG AGAACCACAC ACTAAAACTA TTTCAAGGAT CTAGTGCACA CATATACATT ATTGTTGTAC

**Haruna Nijo**  ATGCTCCCTG TAAGCTCC-A TCCATTCAGA CCAATCGCTG AGAACCACAC ACTAAAACTA TTTCAAGGAT CTAGTGCACA CATATACATT ATTGTTGTAC

**L35**  ATGCTCCCTG TAAGCTCC-A TCCATCCAGA CCAATCGCTG AGAACCACAC ACTAAAACTA TTTCAAGGAT CTAGTGCACA CATATACATT ATTGTTGTAC

**L48**  ATGCTCCCTG TAAGCTCC-A TCCATTCAGA CCAATCGCTG AGAACCACAC ACTAAAACTA TTTCAAGGAT CTAGTGCACA CATATACATT ATTGTTGTAC

**L68**  ATGCTCCCTG TAAGCTCC-A TCCATTCAGA CCAATCGCTG AGAACCACAC ACTAAAACTA TTTCAAGGAT CTAGTGCACA CATATACATT ATTGTTGTAC

**PI296897**  ATGCTCCCTG TAAGCTCC-A TCCATTCAGA CCAATCGCTG AGAACCACAC ACTAAAACTA TTTCAAGGAT CTAGTGCACA CATATACATT ATTGTTGTAC

**AB75**  ATGCTCCCTG TAAGCTCC-A TCCATTCAGA CCAATCGCTG AGAACCACAC ACTAAAACTA TTTCAAGGAT CTAGTGCACA CATATACATT ATTGTTGTAC

**m279**  ATGCTCCCTG TAAGCTCC-A TCCATTCAGA CCAATCGCTG AGAACCACAC ACTAAAACTA TTTCAAGGAT CTAGTGCACA CATATACATT ATTGTTGTAC

**Legacy**  ATGCTCCCTG TAAGCTCC-A TCCATTCAGA CCAATCGCTG AGAACCACAC ACTAAAACTA TTTCAAGGAT CTAGTGCACA CATATACATT ATTGTTGTAC

**Orca**  ATGCTCCCTG TAAGCTCC-A TCCATTCAGA CCAATCGCTG AGAACCACAC ACTAAAACTA TTTCAAGGAT CTAGTGCACA CATATACATT ATTGTTGTAC

**Tango**  ATGCTCCCTG TAAGCTCC-A TCCATTCAGA CCAATCGCTG AGAACCACAC ACTAAAACTA TTTCAAGGAT CTAGTGCACA CATATACATT ATTGTTGTAC

**UC958**  ATGCTCCCTG TAAGCTCC-A TCCATTCAGA CCAATCGCTG AGAACCACAC ACTAAAACTA TTTCAAGGAT CTAGTGCACA CATATACATT ATTGTTGTAC

**UC960**  ATGCTCCCTG TAAGCTCC-A TCCATTCAGA CCAATCGCTG AGAACCACAC ACTAAAACTA TTTCAAGGAT CTAGTGCACA CATATACATT ATTGTTGTAC

**Steptoe**  ATGCTCCCTG TAAGCTCC-A TCCATTCAGA CCAATCGCTG AGAACCACAC ACTAAAACTA TTTCAAGGAT CTAGTGCACA CATATACATT ATTGTTGTAC

**Stander**  ATGCTCCCTG TAAGCTCC-A TCCATTCAGA CCAATCGCTG AGAACCACAC ACTAAAACTA TTTCAAGGAT CTAGTGCACA CATATACATT ATTGTTGTAC

**Ashqelon**  ATGCTCCCTG TAAGCTCC-A TCCATTCAGA CCAATCGCTG AGAACCACAC ACTAAAACTA TTTCAAGGAT CTAGTGCACA CATATACATT ATTGTTGTAC

**L46**  ATGCTCCCTG TAAGCTCC-A TCCATTCAGA CCAATCGCTG AGAACCACAC ACTAAAACTA TTTCAAGGAT CTAGTGCACA CATATACATT ATTGTTGTAC

**....|....| ....|....| ....|....| ....|....| ....|....| ....|....| ....|....| ....|....| ....|....| ....|....|**

**1510 1520 1530 1540 1550 1560 1570 1580 1590 1600**

**Morex**  ATATAACATT GATACTTCTT GTAAAACTCT AATTCAAAGG GTGAAGAACA AGATCTGAGG CCTCAAATGA GTATTTTATT TGTACTAACC TTGACTACAC

**Harrington**  ATATAACATT GATACTTCTT GTAAAACTCT AATTCAAAGG GTGAAGAACA AGATCTGAGG CCTCAAATGA GTATTTTATT TGTACTAACC TTGACTACAC

**HA52**  ATATAACATT GATACTTCTT GTAAAACTCT AATTCAAAGG GTGAAGAACA AGATCTGAGG CCTCAAATGA GTATCTTATT TGTACTAACC TTGACTACAC

**z043**  ATATAACATT GATACTTCTT GTAAAACTCT AATTCAAAGG GTGAAGAACA AGATCTGAGG CCTCAAATGA GTATTTTATT TGTACTAACC TTGACTACAC

**L47**  ATATAACATT GATACTTCTT GTAAAACTCT AATTCAAAGG GTGAAGAACA AGATCTGAGG CCTCAAATGA GTATCTTATT TGTACTAACC TTGACTACAC

**Strider**  ATATAACATT GATACTTCTT GTAAAACTCT AATTCAAAGG GTGAAGAACA AGATCTGAGG CCTCAAATGA GTATTTTATT TGTACTAACC TTGACTACAC

**W127**  ATATAACATT GATACTTCTT GTAAAACTCT AATTCAAAGG GTGAAGAACA AGATCTGAGG CCTCAAATGA GTATTTTATT TGTACTAACC TTGACTACAC

**Adorra**  ATATAACATT GATACTTCTT GTAAAACTCT AATTCAAAGG GTGAAGAACA AGATCTGAGG CCTCAAATGA GTATTTTATT TGTACTAACC TTGACTACAC

**Haruna Nijo**  ATATAACATT GATACTTCTT GTAAAACTCT AATTCAAAGG GTGAAGAACA AGATCTGAGG CCTCAAATGA GTATTTTATT TGTACTAACC TTGACTACAC

**L35**  ATATAACATT GATACTTCTT GTAAAACTCT AATTCAAAGG GTGAAGAACA AGATCTGAGG CCTCAAATGA GTATTTTATT TGTACTAACC TTGACTACAC

**L48**  ATATAACATT GATACTTCTT GTAAAACTCT AATTCAAAGG GTGAAGAACA AGATCTGAGG CCTCAAATGA GTATTTTATT TGTACTAACC TTGACTACAC

**L68**  ATATAACATT GATACTTCTT GTAAAACTCT AATTCAAAGG GTGAAGAACA AGATCTGAGG CCTCAAATGA GTATTTTATT TGTACTAACC TTGACTACAC

**PI296897**  ATATAACATT GATACTTCTT GTAAAACTCT AATTCAAAGG GTGAAGAACA AGATCTGAGG CCTCAAATGA GTATTTTATT TGTACTAACC TTGACTACAC

**AB75**  ATATAACATT GATACTTCTT GTAAAACTCT AATTCAAAGG GTGAAGAACA AGATCTGAGG CCTCAAATGA GTATTTTATT TGTACTAACC TTGACTACAC

**m279**  ATATAACATT GATACTTCTT GTAAAACTCT AATTCAAAGG GTGAAGAACA AGATCTGAGG CCTCAAATGA GTATTTTATT TGTACTAACC TTGACTACAC

**Legacy**  ATATAACATT GATACTTCTT GTAAAACTCT AATTCAAAGG GTGAAGAACA AGATCTGAGG CCTCAAATGA GTATTTTATT TGTACTAACC TTGACTACAC

**Orca**  ATATAACATT GATACTTCTT GTAAAACTCT AATTCAAAGG GTGAAGAACA AGATCTGAGG CCTCAAATGA GTATTTTATT TGTACTAACC TTGACTACAC

**Tango**  ATATAACATT GATACTTCTT GTAAAACTCT AATTCAAAGG GTGAAGAACA AGATCTGAGG CCTCAAATGA GTATTTTATT TGTACTAACC TTGACTACAC

**UC958**  ATATAACATT GATACTTCTT GTAAAACTCT AATTCAAAGG GTGAAGAACA AGATCTGAGG CCTCAAATGA GTATTTTATT TGTACTAACC TTGACTACAC

**UC960**  ATATAACATT GATACTTCTT GTAAAACTCT AATTCAAAGG GTGAAGAACA AGATCTGAGG CCTCAAATGA GTATTTTATT TGTACTAACC TTGACTACAC

**Steptoe**  ATATAACATT GATACTTCTT GTAAAACTCT AATTCAAAGG GTGAAGAACA AGATCTGAGG CCTCAAATGA GTATTTTATT TGTACTAACC TTGACTACAC

**Stander**  ATATAACATT GATACTTCTT GTAAAACTCT AATTCAAAGG GTGAAGAACA AGATCTGAGG CCTCAAATGA GTATTTTATT TGTACTAACC TTGACTACAC

**Ashqelon**  ATATAACATT GATACTTCTT GTAAAACTCT AATTCAAAGG GTGAAGAACA AGATCTGAGG CCTCAAATGA GTATTTTATT TGTACTAACC TTGACTACAC

**L46**  ATATAACATT GATACTTCTT GTAAAACTCT AATTCAAAGG GTGAAGAACA AGATCTGAGG CCTCAAATGA GTATTTTATT TGTACTAACC TTGACTACAC

**....|....| ....|....| ....|....| ....|....| ....|....| ....|....| ....|....| ....|....| ....|....| ....|....|**

**1610 1620 1630 1640 1650 1660 1670 1680 1690 1700**

**Morex**  TTCCATTGTT GAAATAAATA ----GCTGGA CGCCGTGAGC GTGAACAACA GGTTCGAGAA GGGCGACGAG CTGAGGGCGC AATTGAGGAA GCTGGTAGAG

**Harrington**  TTCCATTGTT GAAATAAATA ----GCTGGA CGCCGTGAGC GTGAACAACA GGTTCGAGAA GGGCGACGAG CTGAGGGCGC AATTGAGGAA GCTGGTAGAG

**HA52**  TTCCATTGTT GAAATAAATA ----GCTGGA CGCCGTGAGC GTGAACAACA GGTTCGAGAA GGGCGACGAG CTGAGGGCGC AATTGAGGAA GCTGGTAGAG

**z043**  TTCCATTGTT GAAATAAATA ----GCTGGA CGCCGTGAGC GTGAACAACA GGTTCGAGAA GGGCGACGAG CTGAGGGCGC AATTGAGGAA GCTGGTAGAG

**L47**  TTCCATTGTT GAAATAAATA ----GCTGGA CGCCGTGAGC GTGAACAACA GGTTCGAGAA GGGCGACGAG CTGAGGGCGC AATTGAGGAA GCTGGTAGAG

**Strider**  TTCCATTGTT GAAATAAATA AATAGCTGGA CGCCGTGAGC GTGAACAACA GGTTCGAGAA GGGCGACGAG CTGAGGGCGC AATTGAGGAA GCTGGTAGAG

**W127**  TTCCATTGTT GAAATAAATA ----GCTGGA CGCCGTGAGC GTGAACAACA GGTTCGAGAA GGGCGACGAG CTGAGGGCGC AATTGAGGAA GCTGGTAGAG

**Adorra**  TTCCATTGTT GAAATAAATA AATAGCTGGA CGCCGTGAGC GTGAACAACA GGTTCGAGAA GGGCGACGAG CTGAGGGCGC AATTGAGGAA GCTGGTAGAG

**Haruna Nijo**  TTCCATTGTT GAAATAAATA AATAGCTGGA CGCCGTGAGC GTGAACAACA GGTTCGAGAA GGGCGACGAG CTGAGGGCGC AATTGAGGAA GCTGGTAGAG

**L35**  TTCCATTGTT GAAATAAATA AATAGCTGGA CGCCGTGAGC GTGAACAACA GGTTCGAGAA GGGCGACGAG CTGAGGGCGC AATTGAGGAA GCTGGTAGAG

**L48**  TTCCATTGTT GAAATAAATA AATAGCTGGA CGCCGTGAGC GTGAACAACA GGTTCGAGAA GGGCGACGAG CTGAGGGCGC AATTGAGGAA GCTGGTAGAG

**L68**  TTCCATTGTT GAAATAAATA AATAGCTGGA CGCCGTGAGC GTGAACAACA GGTTCGAGAA GGGCGACGAG CTGAGGGCGC AATTGAGGAA GCTGGTAGAG

**PI296897**  TTCCATTGTT GAAATAAATA AATAGCTGGA CGCCGTGAGC GTGAACAACA GGTTCGAGAA GGGCGACGAG CTGAGGGCGC AATTGAGGAA GCTGGTAGAG

**AB75**  TTCCATTGTT GAAATAAATA AATAGCTGGA CGCCGTGAGC GTGAACAACA GGTTCGAGAA GGGCGACGAG CTGAGGGCGC AATTGAGGAA GCTGGTAGAG

**m279**  TTCCATTGTT GAAATAAATA AATAGCTGGA CGCCGTGAGC GTGAACAACA GGTTCGAGAA GGGCGACGAG CTGAGGGCGC AATTGAGGAA GCTGGTAGAG

**Legacy**  TTCCATTGTT GAAATAAATA AATAGCTGGA CGCCGTGAGC GTGAACAACA GGTTCGAGAA GGGCGACGAG CTGAGGGCGC AATTGAGGAA GCTGGTAGAG

**Orca**  TTCCATTGTT GAAATAAATA AATAGCTGGA CGCCGTGAGC GTGAACAACA GGTTCGAGAA GGGCGACGAG CTGAGGGCGC AATTGAGGAA GCTGGTAGAG

**Tango**  TTCCATTGTT GAAATAAATA AATAGCTGGA CGCCGTGAGC GTGAACAACA GGTTCGAGAA GGGCGACGAG CTGAGGGCGC AATTGAGGAA GCTGGTAGAG

**UC958**  TTCCATTGTT GAAATAAATA AATAGCTGGA CGCCGTGAGC GTGAACAACA GGTTCGAGAA GGGCGACGAG CTGAGGGCGC AATTGAGGAA GCTGGTAGAG

**UC960**  TTCCATTGTT GAAATAAATA AATAGCTGGA CGCCGTGAGC GTGAACAACA GGTTCGAGAA GGGCGACGAG CTGAGGGCGC AATTGAGGAA GCTGGTAGAG

**Steptoe**  TTCCATTGTT GAAATAAATA AATAGCTGGA CGCCGTGAGC GTGAACAACA GGTTCGAGAA GGGCGACGAG CTGAGGGCGC AATTGAGGAA GCTGGTAGAG

**Stander**  TTCCATTGTT GAAATAAATA AATAGCTGGA CGCCGTGAGC GTGAACAACA GGTTCGAGAA GGGCGACGAG CTGAGGGCGC AATTGAGGAA GCTGGTAGAG

**Ashqelon**  TTCCATTGTT GAAATAAATA ----GCTGGA CGCCGTGAGC GTGAACAACA GGTTCGAGAA GGGCGACGAG CTGAGGGCGC AATTGAGGAA GCTGGTAGAG

**L46**  TTCCATTGTT GAAATAAATA ----GCTGGA CGCCGTGAGC GTGAACAACA GGTTCGAGAA GGGCGACGAG CTGAGGGCGC AATTGAGGAA GCTGGTAGAG

**....|....| ....|....| ....|....| ....|....| ....|....| ....|....| ....|....| ....|....| ....|....| ....|....|**

**1710 1720 1730 1740 1750 1760 1770 1780 1790 1800**

**Morex**  GCCGGTGTGG ATGGTGTCAT GGTAGACGTC TGGTGGGGCT TGGTGGAGGG CAAGGGCCCC AAGGCGTATG ACTGGTCCGC CTACAAGCAG TTGTTTGAGC

**Harrington**  GCCGGTGTGG ATGGTGTCAT GGTAGACGTC TGGTGGGGCT TGGTGGAGGG CAAGGGCCCC AAGGCGTATG ACTGGTCCGC CTACAAGCAG TTGTTTGAGC

**HA52**  GCCGGTGTGG ATGGTGTCAT GGTAGACGTC TGGTGGGGCT TGGTGGAGGG CAAGGGCCCC AAGGCGTATG ACTGGTCCGC CTACAAGCAG TTGTTTGAGC

**z043**  GCCGGTGTGG ATGGTGTCAT GGTAGACGTC TGGTGGGGCT TGGTGGAGGG CAAGGGCCCC AAGGCGTATG ACTGGTCCGC CTACAAGCAG TTGTTTGAGC

**L47**  GCCGGTGTGG ATGGTGTCAT GGTAGACGTC TGGTGGGGCT TGGTGGAGGG CAAGGGCCCC AAGGCGTATG ACTGGTCCGC CTACAAGCAG TTGTTTGAGC

**Strider**  GCCGGTGTGG ATGGTGTCAT GGTAGACGTC TGGTGGGGCT TGGTGGAGGG CAAGGGCCCC AAGGCGTATG ACTGGTCCGC CTACAAGCAG TTGTTTGAGC

**W127**  GCCGGTGTGG ATGGTGTCAT GGTAGACGTC TGGTGGGGCT TGGTGGAGGG CAAGGGCCCC AAGGCGTATG ACTGGTCCGC CTACAAGCAG TTGTTTGAGC

**Adorra**  GCCGGTGTGG ATGGTGTCAT GGTAGACGTC TGGTGGGGCT TGGTGGAGGG CAAGGGCCCC AAGGCGTATG ACTGGTCCGC CTACAAGCAG TTGTTTGAGC

**Haruna Nijo**  GCCGGTGTGG ATGGTGTCAT GGTAGACGTC TGGTGGGGCT TGGTGGAGGG CAAGGGCCCC AAGGCGTATG ACTGGTCCGC CTACAAGCAG TTGTTTGAGC

**L35**  GCCGGTGTGG ATGGTGTCAT GGTAGACGTC TGGTGGGGCT TGGTGGAGGG CAAGGGCCCC AAGGCGTATG ACTGGTCCGC CTACAAGCAG TTGTTTGAGC

**L48**  GCCGGTGTGG ATGGTGTCAT GGTAGACGTC TGGTGGGGCT TGGTGGAGGG CAAGGGCCCC AAGGCGTATG ACTGGTCCGC CTACAAGCAG TTGTTTGAGC

**L68**  GCCGGTGTGG ATGGTGTCAT GGTAGACGTC TGGTGGGGCT TGGTGGAGGG CAAGGGCCCC AAGGCGTATG ACTGGTCCGC CTACAAGCAG TTGTTTGAGC

**PI296897**  GCCGGTGTGG ATGGTGTCAT GGTAGACGTC TGGTGGGGCT TGGTGGAGGG CAAGGGCCCC AAGGCGTATG ACTGGTCCGC CTACAAGCAG TTGTTTGAGC

**AB75**  GCCGGTGTGG ATGGTGTCAT GGTAGACGTC TGGTGGGGCT TGGTGGAGGG CAAGGGCCTC AAGGCGTATG ACTGGTCCGC CTACAAGCAG TTGTTTGAGC

**m279**  GCCGGTGTGG ATGGTGTCAT GGTAGACGTC TGGTGGGGCT TGGTGGAGGG CAAGGGCCCC AAGGCGTATG ACTGGTCCGC CTACAAGCAG TTGTTTGAGC

**Legacy**  GCCGGTGTGG ATGGTGTCAT GGTAGACGTC TGGTGGGGCT TGGTGGAGGG CAAGGGCCCC AAGGCGTATG ACTGGTCCGC CTACAAGCAG TTGTTTGAGC

**Orca**  GCCGGTGTGG ATGGTGTCAT GGTAGACGTC TGGTGGGGCT TGGTGGAGGG CAAGGGCCCC AAGGCGTATG ACTGGTCCGC CTACAAGCAG TTGTTTGAGC

**Tango**  GCCGGTGTGG ATGGTGTCAT GGTAGACGTC TGGTGGGGCT TGGTGGAGGG CAAGGGCCCC AAGGCGTATG ACTGGTCCGC CTACAAGCAG TTGTTTGAGC

**UC958**  GCCGGTGTGG ATGGTGTCAT GGTAGACGTC TGGTGGGGCT TGGTGGAGGG CAAGGGCCCC AAGGCGTATG ACTGGTCCGC CTACAAGCAG TTGTTTGAGC

**UC960**  GCCGGTGTGG ATGGTGTCAT GGTAGACGTC TGGTGGGGCT TGGTGGAGGG CAAGGGCCCC AAGGCGTATG ACTGGTCCGC CTACAAGCAG TTGTTTGAGC

**Steptoe**  GCCGGTGTGG ATGGTGTCAT GGTAGACGTC TGGTGGGGCT TGGTGGAGGG CAAGGGCCCC AAGGCGTATG ACTGGTCCGC CTACAAGCAG TTGTTTGAGC

**Stander**  GCCGGTGTGG ATGGTGTCAT GGTAGACGTC TGGTGGGGCT TGGTGGAGGG CAAGGGCCCC AAGGCGTATG ACTGGTCCGC CTACAAGCAG TTGTTTGAGC

**Ashqelon**  GCCGGTGTGG ATGGTGTCAT GGTAGACGTC TGGTGGGGCT TGGTGGAGGG CAAGGGCCCC AAGGCGTATG ACTGGTCCGC CTACAAGCAG TTGTTTGAGC

**L46**  GCCGGTGTGG ATGGTGTCAT GGTAGACGTC TGGTGGGGCT TGGTGGAGGG CAAGGGCCCC AAGGCGTATG ACTGGTCCGC CTACAAGCAG TTGTTTGAGC

**....|....| ....|....| ....|....| ....|....| ....|....| ....|....| ....|....| ....|....| ....|....| ....|....|**

**1810 1820 1830 1840 1850 1860 1870 1880 1890 1900**

**Morex**  TGGTGCAGAA GGCTGGGCTG AAGCTACAGG CCATCATGTC GTTCCACCAG TGTGGTGGCA ACGTCGGCGA CGCCGTCAAC ATCCCAATCC CACAGTGGGT

**Harrington**  TGGTGCAGAA GGCTGGGCTG AAGCTACAGG CCATCATGTC GTTCCACCAG TGTGGTGGCA ACGTCGGCGA CGCCGTCAAC ATCCCAATCC CACAGTGGGT

**HA52**  TGGTGCAGAA GGCTGGGCTG AAGCTACAGG CCATCATGTC GTTCCACCAG TGTGGTGGCA ACGTCGGCGA CGCCGTCAAC ATCCCAATCC CACAGTGGGT

**z043**  TGGTGCAGAA GGCTGGGCTG AAGCTACAGG CCATCATGTC GTTCCACCAG TGTGGTGGCA ACGTCGGCGA CGCCGTCAAC ATCCCAATCC CACAGTGGGT

**L47**  TGGTGCAGAA GGCTGGGCTG AAGCTACAGG CCATCATGTC GTTCCACCAG TGTGGTGGCA ACGTCGGCGA CGCCGTCAAC ATCCCAATCC CACAGTGGGT

**Strider**  TGGTGCAGAA GGCTGGGCTG AAGCTACAGG CCATCATGTC GTTCCACCAG TGTGGTGGCA ACGTCGGCGA CGCCGTCAAC ATCCCAATCC CACAGTGGGT

**W127**  TGGTGCAGAA GGCTGGGCTG AAGCTACAGG CCATCATGTC GTTCCACCAG TGTGGTGGCA ACGTCGGCGA CGCCGTCAAC ATCCCAATCC CACAGTGGGT

**Adorra**  TGGTGCAGAA GGCTGGGCTG AAGCTACAGG CCATCATGTC GTTCCACCAG TGTGGTGGCA ACGTCGGCGA CGCCGTCAAC ATCCCAATCC CACAGTGGGT

**Haruna Nijo**  TGGTGCAGAA GGCTGGGCTG AAGCTACAGG CCATCATGTC GTTCCACCAG TGTGGTGGCA ACGTCGGCGA CGCCGTCAAC ATCCCAATCC CACAGTGGGT

**L35**  TGGTGCAGAA GGCTGGGCTG AAGCTACAGG CCATCATGTC GTTCCACCAG TGTGGTGGCA ACGTCGGCGA CGCCGTCAAC ATCCCAATCC CACAGTGGGT

**L48**  TGGTGCAGAA GGCTGGGCTG AAGCTACAGG CCATCATGTC GTTCCACCAG TGTGGTGGCA ACGTCGGCGA CGCCGTCAAC ATCCCAATCC CACAGTGGGT

**L68**  TGGTGCAGAA GGCTGGGCTG AAGCTACAGG CCATCATGTC GTTCCACCAG TGTGGTGGCA ACGTCGGCGA CGCCGTCAAC ATCCCAATCC CACAGTGGGT

**PI296897**  TGGTGCAGAA GGCTGGGCTG AAGCTACAGG CCATCATGTC GTTCCACCAG TGTGGTGGCA ACGTCGGCGA CGCCGTCAAC ATCCCAATCC CACAGTGGGT

**AB75**  TGGTGCAGAA GGCTGGGCTG AAGCTACAGG CCATCATGTC GTTCCACCAG TGTGGTGGCA ACGTCGGCGA CGCCGTCAAC ATCCCAATCC CACAGTGGGT

**m279**  TGGTGCAGAA GGCTGGGCTG AAGCTACAGG CCATCATGTC GTTCCACCAG TGTGGTGGCA ACGTCGGCGA CGCCGTCAAC ATCCCAATCC CACAGTGGGT

**Legacy**  TGGTGCAGAA GGCTGGGCTG AAGCTACAGG CCATCATGTC GTTCCACCAG TGTGGTGGCA ACGTCGGCGA CGCCGTCAAC ATCCCAATCC CACAGTGGGT

**Orca**  TGGTGCAGAA GGCTGGGCTG AAGCTACAGG CCATCATGTC GTTCCACCAG TGTGGTGGCA ACGTCGGCGA CGCCGTCAAC ATCCCAATCC CACAGTGGGT

**Tango**  TGGTGCAGAA GGCTGGGCTG AAGCTACAGG CCATCATGTC GTTCCACCAG TGTGGTGGCA ACGTCGGCGA CGCCGTCAAC ATCCCAATCC CACAGTGGGT

**UC958**  TGGTGCAGAA GGCTGGGCTG AAGCTACAGG CCATCATGTC GTTCCACCAG TGTGGTGGCA ACGTCGGCGA CGCCGTCAAC ATCCCAATCC CACAGTGGGT

**UC960**  TGGTGCAGAA GGCTGGGCTG AAGCTACAGG CCATCATGTC GTTCCACCAG TGTGGTGGCA ACGTCGGCGA CGCCGTCAAC ATCCCAATCC CACAGTGGGT

**Steptoe**  TGGTGCAGAA GGCTGGGCTG AAGCTACAGG CCATCATGTC GTTCCACCAG TGTGGTGGCA ACGTCGGCGA CGCCGTCAAC ATCCCAATCC CACAGTGGGT

**Stander**  TGGTGCAGAA GGCTGGGCTG AAGCTACAGG CCATCATGTC GTTCCACCAG TGTGGTGGCA ACGTCGGCGA CGCCGTCAAC ATCCCAATCC CACAGTGGGT

**Ashqelon**  TGGTGCAGAA GGCTGGGCTG AAGCTACAGG CCATCATGTC GTTCCACCAG TGTGGTGGCA ACGTCGGCGA CGCCGTCAAC ATCCCAATCC CACAGTGGGT

**L46**  TGGTGCAGAA GGCTGGGCTG AAGCTACAGG CCATCATGTC GTTCCACCAG TGTGGTGGCA ACGTCGGCGA CGCCGTCAAC ATCCCAATCC CACAGTGGGT

**....|....| ....|....| ....|....| ....|....| ....|....| ....|....| ....|....| ....|....| ....|....| ....|....|**

**1910 1920 1930 1940 1950 1960 1970 1980 1990 2000**

**Morex**  GCGGGACGTC GGCACGTGTG ATCCCGACAT TTTCTACACC GACGGTCACG GGACTAGGAA CATTGAGTAC CTCACTCTTG GAGTTGATAA CCAGCCTCTC

**Harrington**  GCGGGACGTC GGCACGTGTG ATCCCGACAT TTTCTACACC GACGGTCACG GGACTAGGAA CATTGAGTAC CTCACTCTTG GAGTTGATAA CCAGCCTCTC

**HA52**  GCGGGACGTC GGCACGTGTG ATCCCGACAT TTTCTACACC GACGGTCACG GGACTAGGAA CATTGAGTAC CTCACTCTTG GAGTTGATAA CCAGCCTCTC

**z043**  GCGGGACGTC GGCACGTGTG ATCCCGACAT TTTCTACACC GACGGTCACG GGACTAGGAA CATTGAGTAC CTCACTCTTG GAGTTGATAA CCAGCCTCTC

**L47**  GCGGGACGTC GGCACGTGTG ATCCCGACAT TTTCTACACC GACGGTCACG GGACTAGGAA CATTGAGTAC CTCACTCTTG GAGTTGATAA CCAGCCTCTC

**Strider**  GCGGGACGTC GGCACGTGTG ATCCCGACAT TTTCTACACC GACGGTCACG GGACTAGGAA CATTGAGTAC CTCACTCTTG GAGTTGATAA CCAGCCTCTC

**W127**  GCGGGACGTC GGCACGTGTG ATCCCGACAT TTTCTACACC GACGGTCACG GGACTAGGAA CATTGAGTAC CTCACTCTTG GAGTTGATAA CCAGCCTCTC

**Adorra**  GCGGGACGTC GGCACGCGTG ATCCCGACAT TTTCTACACC GACGGTCACG GGACTAGGAA CATTGAGTAC CTCACTCTTG GAGTTGATAA CCAGCCTCTC

**Haruna Nijo**  GCGGGACGTC GGCACGCGTG ATCCCGACAT TTTCTACACC GACGGTCACG GGACTAGGAA CATTGAGTAC CTCACTCTTG GAGTTGATAA CCAGCCTCTC

**L35**  GCGGGACGTC GGCACGCGTG ATCCCGACAT TTTCTACACC GACGGTCACG GGACTAGGAA CATTGAGTAC CTCACTCTTG GAGTTGATAA CCAGCCTCTC

**L48**  GCGGGACGTC GGCACGCGTG ATCCCGACAT TTTCTACACC GACGGTCACG GGACTAGGAA CATTGAGTAC CTCACTCTTG GAGTTGATAA CCAGCCTCTC

**L68**  GCGGGACGTC GGCACGCGTG ATCCCGACAT TTTCTACACC GACGGTCACG GGACTAGGAA CATTGAGTAC CTCACTCTTG GAGTTGATAA CCAGCCTCTC

**PI296897**  GCGGGACGTC GGCACGCGTG ATCCCGACAT TTTCTACACC GACGGTCACG GGACTAGGAA CATTGAGTAC CTCACTCTTG GAGTTGATAA CCAGCCTCTC

**AB75**  GCGGGACGTC GGCACGCGTG ATCCCGACAT TTTCTACACC GACGGTCACG GGACTAGGAA CATTGAGTAC CTCACCCTTG GAGTTGATAA CCAGCCTCTC

**m279**  GCGGGACGTC GGCACGCGTG ATCCCGACAT TTTCTACACC GACGGTCACG GGACTAGGAA CATTGAGTAC CTCACTCTTG GAGTTGATAA CCAGCCTCTC

**Legacy**  GCGGGACGTC GGCACGCGTG ATCCCGACAT TTTCTACACC GACGGTCACG GGACTAGGAA CATTGAGTAC CTCACTCTTG GAGTTGATAA CCAGCCTCTC

**Orca**  GCGGGACGTC GGCACGCGTG ATCCCGACAT TTTCTACACC GACGGTCACG GGACTAGGAA CATTGAGTAC CTCACTCTTG GAGTTGATAA CCAGCCTCTC

**Tango**  GCGGGACGTC GGCACGCGTG ATCCCGACAT TTTCTACACC GACGGTCACG GGACTAGGAA CATTGAGTAC CTCACTCTTG GAGTTGATAA CCAGCCTCTC

**UC958**  GCGGGACGTC GGCACGCGTG ATCCCGACAT TTTCTACACC GACGGTCACG GGACTAGGAA CATTGAGTAC CTCACTCTTG GAGTTGATAA CCAGCCTCTC

**UC960**  GCGGGACGTC GGCACGCGTG ATCCCGACAT TTTCTACACC GACGGTCACG GGACTAGGAA CATTGAGTAC CTCACTCTTG GAGTTGATAA CCAGCCTCTC

**Steptoe**  GCGGGACGTC GGCACGCGTG ATCCCGACAT TTTCTACACC GACGGTCACG GGACTAGGAA CATTGAGTAC CTCACTCTTG GAGTTGATAA CCAGCCTCTC

**Stander**  GCGGGACGTC GGCACGCGTG ATCCCGACAT TTTCTACACC GACGGTCACG GGACTAGGAA CATTGAGTAC CTCACTCTTG GAGTTGATAA CCAGCCTCTC

**Ashqelon**  GCGGGACGTC GGCACGTGTG ATCCCGACAT TTTCTACACC GACGGTCACG GGACTAGGAA CATTGAGTAC CTCACCCTTG GAGTTGATAA CCAGCCTCTC

**L46**  GCGGGACGTC GGCACGTGTG ATCCCGACAT TTTCTACACC GACGGTCACG GGACTAGGAA CATTGAGTAC CTCACCCTTG GAGTTGATAA CCAGCCTCTC

**....|....| ....|....| ....|....| ....|....| ....|....| ....|....| ....|....| ....|....| ....|....| ....|....|**

**2010 2020 2030 2040 2050 2060 2070 2080 2090 2100**

**Morex**  TTCCATGGAA GATCTGCCGT CCAGGTTAAT TTAAACCACC ACTCTAGTTC TCTGATGCAT ATATAGATAT ACATATTTAG ATAGAAGTTC AAGATGACAC

**Harrington**  TTCCATGGAA GATCTGCCGT CCAGGTTAAT TTAAACCACC ACTCTAGTTC TCTGATGCAT ATATAGATAT ACATATTTAG ATAGAAGTTC AAGATGACAC

**HA52**  TTCCATGGAA GATCTGCCGT CCAGGTTAAT TTAAACCACC ACTCTAGTTC TCTGATGCAT ATATAGATAT ACATATTTAG ATAGAAGTTC AAGATGACAC

**z043**  TTCCATGGAA GATCTGCCGT CCAGGTTAAT TTAAACCACC ACTCTAGTTC TCTGATGCAT ATATAGATAT ACATATTTAG ATAGAAGTTC AAGATGACAC

**L47**  TTCCATGGAA GATCTGCCGT CCAGGTTAAT TTAAACCACC ACTCTAGTTC TCTGATGCAT ATATAGATAT ACATATTTAG ATAGAAGTTC AAGATGACAC

**Strider**  TTCCATGGAA GATCTGCCGT CCAGGTTAAT TTAAACCACC ACTCTAGTTC TCTGATGCAT ATATAGATAT ACATATTTAG ATAGAAGTTC AAGATGACAC

**W127**  TTCCATGGAA GATCTGCCGT CCAGGTTAAT TTAAACCACC ACTCTAGTTC TCTGATGCAT ATATAGATAT ACATATTTAG ATAGAAGTTC AAGATGACAC

**Adorra**  TTCCATGGAA GATCTGCCGT CCAGGTTAAT TTAAACCACC ACTCTAGTTC TCTGATGCAT AT-------- ------TTAT ATAGAAGTTC AAGATGACAC

**Haruna Nijo**  TTCCATGGAA GATCTGCCGT CCAGGTTACT TTAAACCACC ACTCTAGTTC TCTGATGCAT AT-------- ------TTAT ATAGAAGTTC AAGATGACAC

**L35**  TTCCATGGAA GATCTGCCGT CCAGGTTAAT TTAAACCACC ACTCTAGTTC TCTGATGCAT AT-------- ------TTAT ATAGAAGTTC AAGATGACAC

**L48**  TTCCATGGAA GATCTGCCGT CCAGGTTAAT TTAAACCACC ACTCTAGTTC TCTGATGCAT AT-------- ------TTAT ATAGAAGTTC AAGATGACAC

**L68**  TTCCATGGAA GATCTGCCGT CCAGGTTAAT TTAAACCACC ACTCTAGTTC TCTGATGCAT AT-------- ------TTAT ATAGAAGTTC AAGATGACAC

**PI296897**  TTCCATGGAA GATCTGCCGT CCAGGTTAAT TTAAACCACC ACTCTAGTTC TCTGATGCAT AT-------- ------TTAT ATAGAAGTTC AAGATGACAC

**AB75**  TTCCATGGAA GATCTGCCGT CCAGGTTAAT TTAAACCACC ACTCTAGTTC TCTGATGCAT AT-------- ------TTAT ATAGAAGTTC AAGATGACAC

**m279**  TTCCATGGAA GATCTGCCGT CCAGGTTAAT TTAAACCACC ACTCTAGTTC TCTGATGCAT AT-------- ------TTAT ATAGAAGTTC AAGATGACAC

**Legacy**  TTCCATGGAA GATCTGCCGT CCAGGTTAAT TTAAACCACC ACTCTAGTTC TCTGATGCAT AT-------- ------TTAT ATAGAAGTTC AAGATGACAC

**Orca**  TTCCATGGAA GATCTGCCGT CCAGGTTAAT TTAAACCACC ACTCTAGTTC TCTGATGCAT AT-------- ------TTAT ATAGAAGTTC AAGATGACAC

**Tango**  TTCCATGGAA GATCTGCCGT CCAGGTTAAT TTAAACCACC ACTCTAGTTC TCTGATGCAT AT-------- ------TTAT ATAGAAGTTC AAGATGACAC

**UC958**  TTCCATGGAA GATCTGCCGT CCAGGTTAAT TTAAACCACC ACTCTAGTTC TCTGATGCAT AT-------- ------TTAT ATAGAAGTTC AAGATGACAC

**UC960**  TTCCATGGAA GATCTGCCGT CCAGGTTAAT TTAAACCACC ACTCTAGTTC TCTGATGCAT AT-------- ------TTAT ATAGAAGTTC AAGATGACAC

**Steptoe**  TTCCATGGAA GATCTGCCGT CCAGGTTAAT TTAAACCACC ACTCTAGTTC TCTGATGCAT AT-------- ------TTAT ATAGAAGTTC AAGATGACAC

**Stander**  TTCCATGGAA GATCTGCCGT CCAGGTTAAT TTAAACCACC ACTCTAGTTC TCTGATGCAT AT-------- ------TTAT ATAGAAGTTC AAGATGACAC

**Ashqelon**  TTCCATGGAA GATCTGCCGT CCAGGTTAAT TTAAACCACC ACTCTAGTTC TCTGATGCAT AT-------- ------TTAT ATAGAAGTTC AAGATGACAC

**L46**  TTCCATGGAA GATCTGCCGT CCAGGTTAAT TTAAACCACC ACTCTAGTTC TCTGATGCAT AT-------- ------TTAT ATAGAAGTTC AAGATGACAC

**....|....| ....|....| ....|....| ....|....| ....|....| ....|....| ....|....| ....|....| ....|....| ....|....|**

**2110 2120 2130 2140 2150 2160 2170 2180 2190 2200**

**Morex**  CAAATACAAG CAAGACGTTA AAAGGTGCCA AAAACTGACA AGCAAAGGAA CAAAACCTAG CCAATGAAAC AGTGTAGAGC CTATCCAAAA AAAAAAA---

**Harrington**  CAAATACAAG CAAGACGTTA AAAGGTGCCA AAAACTGACA AGCAAAGGAA CAAAACCTAG CCAATGAAAC AGTGTAGAGC CTATCCAAAA AAAAAAAA--

**HA52**  CAAATACAAG CAAGACGTTA AAAGGTGCCA AAAACTGACA AGCAAAGGAA CAAAACCTAG CCAATGAAAC AGTGTAGAGC CTATCCAAAA AAAAAA----

**z043**  CAAATACAAG CAAGACGTTA AAAGGTGCCA AAAACTGACA AGCAAAGGAA CAAAACCTAG CCAATGAAAC AGTGTAGAGC CTATCCAAAA AAAAAA----

**L47**  CAAATACAAG CAAGACGTTA AAAGGTGCCA AAAACTGACA AGCAAAGGAA CAAAACCTAG CCAATGAAAC AGTGTAGAGC CTATCCAAAA AAAAAAA---

**Strider**  CAAATACAAG CAAGACGTTA AAAGGTGCCA AAAACTGACA AGCAAAGGAA CAAAACCTAG CCAATGAAAC AGTGTAGAGC CTATCCAAAA AAAAAAA---

**W127**  CAAATACAAG CAAGACGTTA AAAGGTGCCA AAAACTGACA AGCAAAGGAA CAAAACCTAG CCAATGAAAC AGTGTAGAGC CTATCCAAAA AAAAAAAGAA

**Adorra**  CAAATACAAG CAAAAGGTTA AA-GGTGCCA AAAACAGATA AGCAAAGAAA CAAAACCTAG CTAATGAAAC AGTCTAGAGC CTATCAAAAA AAA-------

**Haruna Nijo**  CAAATACAAG CAAAAGGTTA AA-GGTGCCA AAAACAGATA AGCAAAGAAA CAAAACCTAG CTAATGAAAC AGTCTAGAGC CTATCAAAAA AAAAAAAAA-

**L35**  CAAATACAAG CAAAAGGTTA AA-GGTGCCA AAAACAGATA AGCAAAGAAA CAAAACCTAG CTAATGAAAC AGTCTAGAGC CTATCAAAAA AAAAAAAAA-

**L48**  CAAATACAAG CAAAAGGTTA AA-GGTGCCA AAAACAGATA AGCAAAGAAA CAAAACCTAG CTAATGAAAC AGTCTAGAGC CTATCAAAAA AAAAAAAAAA

**L68**  CAAATACAAG CAAAAGGTTA AA-GGTGCCA AAAACAGATA AGCAAAGAAA CAAAACCTAG CTAATGAAAC AGTCTAGAGC CTATCAAAAA AAAAAAAAAA

**PI296897**  CAAATACAAG CAAAAGGTTA AA-GGTGCCA AAAACAGATA AGCAAAGAAA CAAAACCTAG CTAATGAAAC AGTCTAGAGC CTATCAAAAA AAAAAA----

**AB75**  CAAATACAAG CAAAAGGTTA AA-GGTGCCA AAAACAGATA AGCAAAGAAA CAAAACCTAG CTAATGAAAC AGTCTAGAGC CTATCCAAAA AAAA------

**m279**  CAAATACAAG CAAAAGGTTA AA-GGTGCCA AAAACAGATA AGCAAAGAAA CAAAACCTAG CTAATGAAAC AGTCTAGAGC CTATCAAAAA AAA-------

**Legacy**  CAAATACAAG CAAAAGGTTA AA-GGTGCCA AAAACAGATA AGCAAAGAAA CAAAACCTAG CTAATGAAAC AGTCTAGAGC CTATCAAAAA AAAAA-----

**Orca**  CAAATACAAG CAAAAGGTTA AA-GGTGCCA AAAACAGATA AGCAAAGAAA CAAAACCTAG CTAATGAAAC AGTCTAGAGC CTATCAAAAA AAAAA-----

**Tango**  CAAATACAAG CAAAAGGTTA AA-GGTGCCA AAAACAGATA AGCAAAGAAA CAAAACCTAG CTAATGAAAC AGTCTAGAGC CTATCAAAAA AAAAA-----

**UC958**  CAAATACAAG CAAAAGGTTA AA-GGTGCCA AAAACAGATA AGCAAAGAAA CAAAACCTAG CTAATGAAAC AGTCTAGAGC CTATCAAAAA AAA-------

**UC960**  CAAATACAAG CAAAAGGTTA AA-GGTGCCA AAAACAGATA AGCAAAGAAA CAAAACCTAG CTAATGAAAC AGTCTAGAGC CTATCAAAAA AAAAA-----

**Steptoe**  CAAATACAAG CAAAAGGTTA AA-GGTGCCA AAAACAGATA AGCAAAGAAA CAAAACCTAG CTAATGAAAC AGTCTAGAGC CTATCAAAAA AAAAA-----

**Stander**  CAAATACAAG CAAAAGGTTA AA-GGTGCCA AAAACAGATA AGCAAAGAAA CAAAACCTAG CTAATGAAAC AGTCTAGAGC CTATCAAAAA AAAAA-----

**Ashqelon**  CAAATACAAG CAAAAGGTTA AA-GGTGCCA AAAACAGATA AGCAAAGAAA CAAAACCTAG CTAATGAAAC AGTCTAGAGC CTATCCAAAA AAAAAAAGAA

**L46**  CAAATACAAG CAAAAGGTTA AA-GGTGCCA AAAACAGATA AGCAAAGAAA CAAAACCTAG CTAATGAAAC AGTCTAGAGC CTATCCAAAA AAAAAAAGAA

**....|....| ....|....| ....|....| ....|....| ....|....| ....|....| ....|....| ....|....| ....|....| ....|....|**

**2210 2220 2230 2240 2250 2260 2270 2280 2290 2300**

**Morex**  --ACCATCGA GAAGGTGCCT AGAGCGGATG GGTTTCTACA ACCCTTTAGC TTTCATGCAT CTTTCTGGGA AAGGGTGAAA AACACCGTCC TTTAAGTCGA

**Harrington**  --ACCATCGA GAAGGTGCCT AGAGCGGATG GGTTTCTACA ACCCTTTAGC TTTCATGCAT CTTTCTGGGA AAGGGTGAAA AACACCGTCC TTTAAGTCGA

**HA52**  --ACCATCGA GAAGGTGCCT AGAGCGGATG GGTTTCTACA ACCCTTTAGC TTTCATGCAT CTTTCTGGGA AAGGGTGAAA AACACCGTCC TTTAAGTCGA

**z043**  --ACCATCGA GAAGGTGCCT AGAGCGGATG GGTTTCTACA ACCCTTTAGC TTTCATGCAT CTTTCTGGGA AAGGGTGAAA AACACCGTCC TTTAAGTCGA

**L47**  --ACCATCGA GAAGGTGCCT AGAGCGGATG GGTTTCTACA ACCCTTTAGC TTTCATGCAT CTTTCTGGGA AAGGGTGAAA AACACCGTCC TTTAAGTCGA

**Strider**  --ACCATCGA GAAGGTGCCT AGAGCGGATG GGTTTCTACA ACCCTTTAGC TTTCATGCAT CTTTCTGGGA AAGGGTGAAA AACACCGTCC TTTAAGTCGA

**W127**  AAAACATCGA GAAGGTGCCT AGAGCGGATG GGTTTCGACA ACCCTTTAGC TTTCATGCAT CTTTCTGGGA AAGGGTGAAA AACACCGTCC TTTAAGTCGA

**Adorra**  --AACATGGA GAAGGTGCCT AGAGCGGATG GGTTTCGACA ACCCTTTAGC TTTCATGCAT CTTTTTGGGA AAGGGTGAAA AACACCGTCC TTTAAGTCGA

**Haruna Nijo**  --AACATCGA GAAGGTGCCT AGAGCGGATG GGTTTCGACA ACCCTTTAGC TTTCATGCAT CTTTTTGGGA AAGGGTGAAA AACACCGTCC TTTAAGTCGA

**L35**  --AACACCGA GAAGGTGCCT AGAGCGGATG GGTTTCGACA ACCCTTTAGC TTTCATGCAT CTTTCTGGGA AAGGGTGAAA AACACCGTCC TTTAAGTCGA

**L48**  --AACACCGA GAAGGTGCCT AGAGCGGATG GGTTTCGACA ACCCTTTAGC TTTCATGCAT CTTTCTGGGA AAGGGTGAAA AACACCGTCC TTTAAGTCGA

**L68**  --AACACCGA GAAGGTGCCT AGAGCGGATG GGTTTCGACA ACCCTTTAGC TTTCATGCAT CTTTCTGGGA AAGGGTGAAA AACACCGTCC TTTAAGTCGA

**PI296897**  --AACATCGA GAAGGTGCCT AGAGCGGATG GGTTTCGACA ACCCTTTAGC TTTCATGCAT CTTTTTGGGA AAGGGTGAAA AACACCGTCC TTTAAGTCGA

**AB75**  --AACATCGA GAAGGTGCCT AGAGCGGATG GGTTTCGACA ACCCTTTAGC TTTCATGCAT CTTTTTGGGA GAGGATGAAA AACACCGTCC TTTAAGTCGA

**m279**  --AACATGGA GAAGGTGCCT AGAGCGGATG GGTTTCGACA ACCCTTTAGC TTTCATGCAT CTTTCTGGGA AAGGGTGAAA AACACCGTCC TTTAAGTCGA

**Legacy**  --AACATCGA GAAGGTGCCT AGAGCGGATG GGTTTCGACA ACCCTTTAGC TTTCATGCAT CTTTTTGGGA AAGGGTGAAA AACACCGTCC TTTAAGTCGA

**Orca**  --AACATCGA GAAGGTGCCT AGAGCGGATG GGTTTCGACA ACCCTTTAGC TTTCATGCAT CTTTTTGGGA AAGGGTGAAA AACACCGTCC TTTAAGTCGA

**Tango**  --AACATCGA GAAGGTGCCT AGAGCGGATG GGTTTCGACA ACCCTTTAGC TTTCATGCAT CTTTTTGGGA AAGGGTGAAA AACACCGTCC TTTAAGTCGA

**UC958**  --AACATCGA GAAGGTGCCT AGAGCGGATG GGTTTCGACA ACCCTTTAGC TTTCATGCAT CTTTTTGGGA AAGGGTGAAA AACACCGTCC TTTAAGTCGA

**UC960**  --AACATCGA GAAGGTGCCT AGAGCGGATG GGTTTCGACA ACCCTTTAGC TTTCATGCAT CTTTTTGGGA AAGGGTGAAA AACACCGTCC TTTAAGTCGA

**Steptoe**  --AACATCGA GAAGGTGCCT AGAGCGGATG GGTTTCGACA ACCCTTTAGC TTTCATGCAT CTTTTTGGGA AAGGGTGAAA AACACCGTCC TTTAAGTCGA

**Stander**  --AACATCGA GAAGGTGCCT AGAGCGGATG GGTTTCGACA ACCCTTTAGC TTTCATGCAT CTTTTTGGGA AAGGGTGAAA AACACCGTCC TTTAAGTCGA

**Ashqelon**  AAAACATCGA GAAGGTGCCT AGAGCGGATG GGTTTCGACA ACCCTTTAGC TTTCATGCAT CTTTTTGGGA AAGGGTGAAA AACACCGTCC TTTAAGTCGA

**L46**  AAAACATCGA GAAGGTGCCT AGAGCGGATG GGTTTCGACA ACCCTTTAGC TTTCATGCAT CTTTTTGGGA AAGGGTGAAA AACACCGTCC TTTAAGTCGA

**....|....| ....|....| ....|....| ....|....| ....|....| ....|....| ....|....| ....|....| ....|....| ....|....|**

**2310 2320 2330 2340 2350 2360 2370 2380 2390 2400**

**Morex**  TTGATGCAGG CAG-CCTTCT ATTGTTTGTA AGCTATCAGG AAATACAAAA TTAATAGATA GTT-GTCATT TTAATAGTTG TAGCAAGCTT TGATTCTTCT

**Harrington**  TTGATGCAGG CAG-CCTTCT ATTGTTTGTA AGCTATCAGG AAATACAAAA TTAATAGATA GTT-GTCATT TTAATAGTTG TAGCAAGCTT TGATTCTTCT

**HA52**  TTGATGCAGG CAG-CCTTCT ATTGTTTGTA AGCTATCAGG AAATACAAAA TTAATAGATA GTT-GTCATT TTAATAGTTG TAGCAAGCTT TGATTCTTCT

**z043**  TTGATGCAGG CAG-CCTTCT ATTGTTTGTA AGCTATCAGG AAATACAAAA TTAATAGATA GTT-GTCATT TTAATAGTTG TAGCAAGCTT TGATTCTTCT

**L47**  TTGATGCAGG CAG-CCTTCT ATTGTTTGTA AGCTATCAGG AAATACAAAA TTAATAGCTA GTT-GTCATT TTAATAGTTG TAGCAAGCTT TGATTCTTCT

**Strider**  TTGATGCAGG CAG-CCTTCT ATTGTTTGTA AGCTATCAGG AAATACAAAA TTAATAGATA GTT-GTCATT TTAATAGTTG TAGCAAGCTT TGATTCTTCT

**W127**  TTGATGCAGG CAG-CCTTCT ATTGTTTGTA AGCTATCAGG AAATACAAAA TTAATAGCTA GTT-GTCATT TTAATAGTTG TAGCAACCTT TGATTCTTCT

**Adorra**  TTGATGCAGG CAG-CCTTCT ATTGTTTGTA AGCTATCAGG AAATACAAAA TTAATAGCTA GTT-GTCATT TTAATAGTTG TAGCAAGCTT TGATTCTTCT

**Haruna Nijo**  TTGATGCAGG CAG-CCTTCT ATTGTTTGTA AGCTATCAGG AAATACAAAA TTAATAGCTA GTT-GTCATT TTAATAGTTG TAGCAAGCTT TGATTCTTCT

**L35**  TTGATGCAGG CAG-CCTTCT ATTGTTTGTA AGCTATCAGG AAATACAAAA TTAATAGCTA GTT-GTCATT TTAATAGTTG TAGCAAGCTT TGATTTTTCT

**L48**  TTGATGCAGG CAG-CCTTCT ATTGTTTGTA AGCTATCAGG AAATACAAAA TTAATAGCTA GTT-GTCATT TTAATAGTTG TAGCAAGCTT TGATTTTTCT

**L68**  TTGATGCAGG CAG-CCTTCT ATTGTTTGTA AGCTATCAGG AAATACAAAA TTAATAGCTA GTT-GTCATT TTAATAGTTG TAGCAAGCTT TGATTTTTCT

**PI296897**  TTGATGCAGG CAG-CCTTCT ATTGTTTGTA AGCTATCAGG AAATACAAAA TTAATAGCTA GTT-GTCATT TTAATAGTTG TAGCAAGCTT TGATTTTTCT

**AB75**  TTGATGCAGG CAGTCCTTCT ATTGTTTGTA AGCTATCAGG AAATACAAAA TCATTAGCTA GTTTGTCATT TTAATAGTTG TAGCAACCTT TGATTCTTCT

**m279**  TTGATGCAGG CAG-CCTTCT ATTGTTTGTA AGCTATCAGG AAATACAAAA TTAATAGCTA GTT-GTCATT TTAATAGTTG TAGCAAGCTT TGATTCTTCT

**Legacy**  TTGATGCAGG CAG-CCTTCT ATTGTTTGTA AGCTATCAGG AAATACAAAA TTAATAGCTA GTT-GTCATT TTAATAGTTG TAGCAAGCTT TGATTCTTCT

**Orca**  TTGATGCAGG CAG-CCTTCT ATTGTTTGTA AGCTATCAGG AAATACAAAA TTAATAGCTA GTT-GTCATT TTAATAGTTG TAGCAAGCTT TGATTCTTCT

**Tango**  TTGATGCAGG CAG-CCTTCT ATTGTTTGTA AGCTATCAGG AAATACAAAA TTAATAGCTA GTT-GTCATT TTAATAGTTG TAGCAAGCTT TGATTCTTCT

**UC958**  TTGATGCAGG CAG-CCTTCT ATTGTTTGTA AGCTATCAGG AAATACAAAA TTAATAGCTA GTT-GTCATT TTAATAGTTG TAGCAAGCTT TGATTCTTCT

**UC960**  TTGATGCAGG CAG-CCTTCT ATTGTTTGTA AGCTATCAGG AAATACAAAA TTAATAGCTA GTT-GTCATT TTAATAGTTG TAGCAAGCTT TGATTCTTCT

**Steptoe**  TTGATGCAGG CAG-CCTTCT ATTGTTTGTA AGCTATCAGG AAATACAAAA TTAATAGCTA GTT-GTCATT TTAATAGTTG TAGCAAGCTT TGATTCTTCT

**Stander**  TTGATGCAGG CAG-CCTTCT ATTGTTTGTA AGCTATCAGG AAATACAAAA TTAATAGCTA GTT-GTCATT TTAATAGTTG TAGCAAGCTT TGATTCTTCT

**Ashqelon**  TTGATGCAGG CAG-CCTTCT ATTGTTTGTA AGCTATCAGG AAATACAAAA TTAATAGCTA GTT-GTCATT TTAATAGTTG TAGCAACCTT TGATTCTTCT

**L46**  TTGATGCAGG CAG-CCTTCT ATTGTTTGTA AGCTATCAGG AAATACAAAA TTAATAGCTA GTT-GTCATT TTAATAGTTG TAGCAACCTT TGATTCTTCT

**....|....| ....|....| ....|....| ....|....| ....|....| ....|....| ....|....| ....|....| ....|....| ....|....|**

**2410 2420 2430 2440 2450 2460 2470 2480 2490 2500**

**Morex**  TTTGTGGCTG TGACAGATGT ATGCCGATTA CATGACAAGC TTCAGGGAGA ACATGAAAGA GTTCTTGGAT GCTGGTGTTA TCGTCGACAT TGAAGTAGGA

**Harrington**  TTTGTGGCTG TGACAGATGT ATGCCGATTA CATGACAAGC TTCAGGGAGA ACATGAAAGA GTTCTTGGAT GCTGGTGTTA TCGTCGACAT TGAAGTAGGA

**HA52**  TTTGTGGCTG TGACAGATGT ATGCCGATTA CATGACAAGC TTCAGGGAGA ACATGAAAGA GTTCTTGGAT GCTGGTGTTA TCGTCGACAT TGAAGTAGGA

**z043**  TTTGTGGCTG TGACAGATGT ATGCCGATTA CATGACAAGC TTCAGGGAGA ACATGAAAGA GTTCTTGGAT GCTGGTGTTA TCGTCGACAT TGAAGTAGGA

**L47**  TTTGTGGCTG TGACAGATGT ATGCCGATTA CATGACAAGC TTCAGGGAGA ACATGAAAGA GTTCTTGGAT GCTGGTGTTA TCGTCGACAT TGAAGTGGGA

**Strider**  TTTGTGGCTG TGACAGATGT ATGCCGATTA CATGACAAGC TTCAGGGAGA ACATGAAAGA GTTCTTGGAT GCTGGTGTTA TCGTCGACAT TGAAGTGGGA

**W127**  TTTGTGGCTG TGACAGATGT ATGCCGATTA CATGACAAGC TTCAGGGAGA ACATGAAAGA CTTCTTGGAT GCTGGTGTTA TCGTCGACAT TGAAGTGGGA

**Adorra**  TTTGTGGCTG TGACAGATGT ATGCCGATTA CATGACAAGC TTCAGGGAGA ACATGAAAGA CTTCTTGGAT GCTGGTGTTA TCGTCGACAT TGAAGTGGGA

**Haruna Nijo**  TTTGTGGCTG TGACAGATGT ATGCCGATTA CATGACAAGC TTCAGGGAGA ACATGAAAGA CTTCTTGGAT GCTGGTGTTA TCGTCGACAT TGAAGTGGGA

**L35**  TTTGTGGCTG TGACAGATGT ATGCCGATTA CATGACAAGC TTCAGGGAGA ACATGAAAGA CTTCTTGGAT GCTGGTGTTA TCGTCGACAT TGAAGTGGGA

**L48**  TTTGTGGCTG TGACAGATGT ATGCCGATTA CATGACAAGC TTCAGGGAGA ACATGAAAGA CTTCTTGGAT GCTGGTGTTA TCGTCGACAT TGAAGTGGGA

**L68**  TTTGTGGCTG TGACAGATGT ATGCCGATTA CATGACAAGC TTCAGGGAGA ACATGAAAGA CTTCTTGGAT GCTGGTGTTA TCGTCGACAT TGAAGTGGGA

**PI296897**  TTTGTGGCTG TGACAGATGT ATGCCGATTA CATGACAAGC TTCAGGGAGA ACATGAAAGA CTTCTTGGAT GCTGGTGTTA TCGTCGACAT TGAAGTGGGA

**AB75**  TTTGTGGCTG TGACAGATGT ATGCCGATTA CATGACAAGC TTCAGGGAGA ACATGAAAGA GTTCTTGGAT GCTGGTGTTA TCGTCGACAT TGAAGTGGGA

**m279**  TTTGTGGCTG TGACAGATGT ATGCCGATTA CATGACAAGC TTCAGGGAGA ACATGAAAGA CTTCTTGGAT GCTGGTGTTA TCGTCGACAT TGAAGTGGGA

**Legacy**  TTTGTGGCTG TGACAGATGT ATGCCGATTA CATGACAAGC TTCAGGGAGA ACATGAAAGA CTTCTTGGAT GCTGGTGTTA TCGTCGACAT TGAAGTGGGA

**Orca**  TTTGTGGCTG TGACAGATGT ATGCCGATTA CATGACAAGC TTCAGGGAGA ACATGAAAGA CTTCTTGGAT GCTGGTGTTA TCGTCGACAT TGAAGTGGGA

**Tango**  TTTGTGGCTG TGACAGATGT ATGCCGATTA CATGACAAGC TTCAGGGAGA ACATGAAAGA CTTCTTGGAT GCTGGTGTTA TCGTCGACAT TGAAGTGGGA

**UC958**  TTTGTGGCTG TGACAGATGT ATGCCGATTA CATGACAAGC TTCAGGGAGA ACATGAAAGA CTTCTTGGAT GCTGGTGTTA TCGTCGACAT TGAAGTGGGA

**UC960**  TTTGTGGCTG TGACAGATGT ATGCCGATTA CATGACAAGC TTCAGGGAGA ACATGAAAGA CTTCTTGGAT GCTGGTGTTA TCGTCGACAT TGAAGTGGGA

**Steptoe**  TTTGTGGCTG TGACAGATGT ATGCCGATTA CATGACAAGC TTCAGGGAGA ACATGAAAGA CTTCTTGGAT GCTGGTGTTA TCGTCGACAT TGAAGTGGGA

**Stander**  TTTGTGGCTG TGACAGATGT ATGCCGATTA CATGACAAGC TTCAGGGAGA ACATGAAAGA CTTCTTGGAT GCTGGTGTTA TCGTCGACAT TGAAGTGGGA

**Ashqelon**  TTTGTGGCTG TGACAGATGT ATGCCGATTA CATGACAAGC TTCAGGGAGA ACATGAAAGA CTTCTTGGAT GCTGGTGTTA TCGTCGACAT TGAAGTGGGA

**L46**  TTTGTGGCTG TGACAGATGT ATGCCGATTA CATGACAAGC TTCAGGGAGA ACATGAAAGA GTTCTTGGAT GCTGGTGTTA TCGTCGACAT TGAAGTGGGA

**....|....| ....|....| ....|....| ....|....| ....|....| ....|....| ....|....| ....|....| ....|....| ....|....|**

**2510 2520 2530 2540 2550 2560 2570 2580 2590 2600**

**Morex**  CTTGGCCCAG CTGGAGAGAT GAGGTACCCA TCATATCCTC AGAGCCACGG ATGGTCGTTC CCAGGCATCG GAGAATTCAT CGTGAGTGTT TGTTTCCAAA

**Harrington**  CTTGGCCCAG CTGGAGAGAT GAGGTACCCA TCATATCCTC AGAGCCACGG ATGGTCGTTC CCAGGCATCG GAGAATTCAT CGTGAGTGTT TGTTTCCAAA

**HA52**  CTTGGCCCAG CTGGAGAGAT GAGGTACCCA TCATATCCTC AGAGCCACGG ATGGTCGTTC CCAGGCATCG GAGAATTCAT CGTGAGTGTT TGTTTCCAAA

**z043**  CTTGGCCCAG CTGGAGAGAT GAGGTACCCA TCATATCCTC AGAGCCACGG ATGGTCGTTC CCAGGCATCG GAGAATTCAT CGTGAGTGTT TGTTTCCAAA

**L47**  CTTGGCCCAG CTGGAGAGAT GAGGTACCCA TCATATCCTC AGAGCCACGG ATGGTCGTTC CCAGGCATCG GAGAATTCAT CGTGAGTGTT TGTTTCCAAA

**Strider**  CTTGGCCCAG CTGGAGAGAT GAGGTACCCA TCATATCCTC AGAGCCACGG ATGGTCGTTC CCAGGCATCG GAGAATTCAT CGTGAGTGTT TGTTTCCAAA

**W127**  CTTGGCCCAG CTGGAGAGAT GAGGTACCCA TCATATCCTC AGAGCCACGG ATGGTCGTTC CCAGGCATCG GAGAATTCAT CGTGAGTGTT TGTTTCCAAA

**Adorra**  CTTGGCCCAG CTGGAGAGAT GAGGTACCCA TCATATCCTC AGAGCCACGG ATGGTCGTTC CCAGGCATCG GAGAATTCAT CGTGAGTGTT TGTTTCCAAA

**Haruna Nijo**  CTTGGCCCAG CTGGAGAGAT GAGGTACCCA TCATATCCTC AGAGCCACGG ATGGTCGTTC CCAGGCATCG GAGAATTCAT CGTGAGTGTT TGTTTCCAAA

**L35**  CTTGGCCCAG CTGGAGAGAT GAGGTACCCA TCATATCCTC AGAGCCACGG ATGGTCGTTC CCAGGCATCG GAGAATTCAT CGTGAGTGTT TGTTTCCAAA

**L48**  CTTGGCCCAG CTGGAGAGAT GAGGTACCCA TCATATCCTC AGAGCCACGG ATGGTCGTTC CCAGGCATCG GAGAATTCAT CGTGAGTGTT TGTTTCCAAA

**L68**  CTTGGCCCAG CTGGAGAGAT GAGGTACCCA TCATATCCTC AGAGCCACGG ATGGTCGTTC CCAGGCATCG GAGAATTCAT CGTGAGTGTT TGTTTCCAAA

**PI296897**  CTTGGCCCAG CTGGAGAGAT GAGGTACCCA TCATATCCTC AGAGCCACGG ATGGTCGTTC CCAGGCATCG GAGAATTCAT CGTGAGTGTT TGTTTCCAAA

**AB75**  CTTGGCCCAG CTGGAGAGAT GAGGTACCCA TCATATCCTC AGAGCCACGG ATGGTCGTTC CCAGGCATCG GAGAATTCAT CGTGAGTGTT TGTTTCCAAA

**m279**  CTTGGCCCAG CTGGAGAGAT GAGGTACCCA TCATATCCTC AGAGCCACGG ATGGTCGTTC CCAGGCATCG GAGAATTCAT CGTGAGTGTT TGTTTCCAAA

**Legacy**  CTTGGCCCAG CTGGAGAGAT GAGGTACCCA TCATATCCTC AGAGCCACGG ATGGTCGTTC CCAGGCATCG GAGAATTCAT CGTGAGTGTT TGTTTCCAAA

**Orca**  CTTGGCCCAG CTGGAGAGAT GAGGTACCCA TCATATCCTC AGAGCCACGG ATGGTCGTTC CCAGGCATCG GAGAATTCAT CGTGAGTGTT TGTTTCCAAA

**Tango**  CTTGGCCCAG CTGGAGAGAT GAGGTACCCA TCATATCCTC AGAGCCACGG ATGGTCGTTC CCAGGCATCG GAGAATTCAT CGTGAGTGTT TGTTTCCAAA

**UC958**  CTTGGCCCAG CTGGAGAGAT GAGGTACCCA TCATATCCTC AGAGCCACGG ATGGTCGTTC CCAGGCATCG GAGAATTCAT CGTGAGTGTT TGTTTCCAAA

**UC960**  CTTGGCCCAG CTGGAGAGAT GAGGTACCCA TCATATCCTC AGAGCCACGG ATGGTCGTTC CCAGGCATCG GAGAATTCAT CGTGAGTGTT TGTTTCCAAA

**Steptoe**  CTTGGCCCAG CTGGAGAGAT GAGGTACCCA TCATATCCTC AGAGCCACGG ATGGTCGTTC CCAGGCATCG GAGAATTCAT CGTGAGTGTT TGTTTCCAAA

**Stander**  CTTGGCCCAG CTGGAGAGAT GAGGTACCCA TCATATCCTC AGAGCCACGG ATGGTCGTTC CCAGGCATCG GAGAATTCAT CGTGAGTGTT TGTTTCCAAA

**Ashqelon**  CTTGGCCCAG CTGGAGAGAT GAGGTACCCA TCATATCCTC AGAGCCACGG ATGGTCGTTC CCAGGCATCG GAGAATTCAT CGTGAGTGTT TGTTTCCAAA

**L46**  CTTGGCCCAG CTGGAGAGAT GAGGTACCCA TCATATCCTC AGAGCCACGG ATGGTCGTTC CCAGGCATCG GAGAATTCAT CGTGAGTGTT TGTTTCCAAA

**....|....| ....|....| ....|....| ....|....| ....|....| ....|....| ....|....| ....|....| ....|....| ....|....|**

**2610 2620 2630 2640 2650 2660 2670 2680 2690 2700**

**Morex**  CTAATAATCT TTCCTCTTCT GTTCCGATCA AATA------ ---------- ---------- ---------- ---------- ---------- ----------

**Harrington**  CTAATAATCT TTCCTCTTCT GTTCCGATCA AATA------ ---------- ---------- ---------- ---------- ---------- ----------

**HA52**  CTAATAATCT TTCCTCTTCT GTTCCGATCA AATA------ ---------- ---------- ---------- ---------- ---------- ----------

**z043**  CTAATAATCT TTCCTCTTCT GTTCCGATCA AATA------ ---------- ---------- ---------- ---------- ---------- ----------

**L47**  CTAATAATCT TTCCTCTTCT GTTCCGATCA AATA------ ---------- ---------- ---------- ---------- ---------- ----------

**Strider**  CTAATAATCT TTCCTCTTCT GTTCCGATCA AATA------ ---------- ---------- ---------- ---------- ---------- ----------

**W127**  CTAATAATCT TTCCTCTTCT GTTCCGATCA AATA------ ---------- ---------- ---------- ---------- ---------- ----------

**Adorra**  CTAATAATCT TTCCTCTTCT GTTCCGATCA AATACTCCCC TCGTCCTAAA ATTCTTGTCT TAGCTTTGTC TAGAAATTGA TGTATCTAAA TACTAAAACT

**Haruna Nijo**  CTAATAATCT TTCCTCTTCT GTTCCGATCA AATA------ ---------- ---------- ---------- ---------- ---------- ----------

**L35**  CTAATAATCT TTCCTCTTCT GTTCCGATCA AATA------ ---------- ---------- ---------- ---------- ---------- ----------

**L48**  CTAATAATCT TTCCTCTTCT GTTCCGATCA AATA------ ---------- ---------- ---------- ---------- ---------- ----------

**L68**  CTAATAATCT TTCCTCTTCT GTTCCGATCA AATA------ ---------- ---------- ---------- ---------- ---------- ----------

**PI296897**  CTAATAATCT TTCCTCTTCT GTTCCGATCA AATA------ ---------- ---------- ---------- ---------- ---------- ----------

**AB75**  CTAATAATCT TTCCTCTTCT GTTCCGATCA AATA------ ---------- ---------- ---------- ---------- ---------- ----------

**m279**  CTAATAATCT TTCCTCTTCT GTTCCGATCA AATACTCCCT TCGTCCTAAA ATTCTTGTCT TAGCTTTGTC TAGAAATTGA TGTATCTAAA TACTAAAACT

**Legacy**  CTAATAATCT TTCCTCTTCT GTTCCGATCA AATACTCCCT CCGTCCTAAA ATTCTTGTCT TAGCTTTGTC TAGAAATTGA TGTATCTAAA TACTAAAACT

**Orca**  CTAATAATCT TTCCTCTTCT GTTCCGATCA AATACTCCCT CCGTCCTAAA ATTCTTGTCT TAGCTTTGTC TAGAAATTGA TGTATCTAAA TACTAAAACT

**Tango**  CTAATAATCT TTCCTCTTCT GTTCCGATCA AATACTCCCT CCGTCCTAAA ATTCTTGTCT TAGCTTTGTC TAGAAATTGA TGTATCTAAA TACTAAAACT

**UC958**  CTAATAATCT TTCCTCTTCT GTTCCGATCA AATACTCCCT CCGTCCTAAA ATTCTTGTCT TAGCTTTGTC TAGAAATTGA TGTATCTAAA TACTAAAACT

**UC960**  CTAATAATCT TTCCTCTTCT GTTCCGATCA AATACTCCCT CCGTCCTAAA ATTCTTGTCT TAGCTTTGTC TAGAAATTGA TGTATCTAAA TACTAAAACT

**Steptoe**  CTAATAATCT TTCCTCTTCT GTTCCGATCA AATACTCCCT CCGTCCTAAA ATTCTTGTCT TAGCTTTGTC TAGAAATTGA TGTATCTAAA TACTAAAACT

**Stander**  CTAATAATCT TTCCTCTTCT GTTCCGATCA AATACTCCCT CCGTCCTAAA ATTCTTGTCT TAGCTTTGTC TAGAAATTGA TGTATCTAAA TACTAAAACT

**Ashqelon**  CTAATAATCT TTCCTCTTCT GTTCCGATCA AATA------ ---------- ---------- ---------- ---------- ---------- ----------

**L46**  CTAATAATCT TTCCTCTTCT GTTCCGATCA AATA------ ---------- ---------- ---------- ---------- ---------- ----------

**....|....| ....|....| ....|....| ....|....| ....|....| ....|....| ....|....| ....|....| ....|....| ....|....|**

**2710 2720 2730 2740 2750 2760 2770 2780 2790 2800**

**Morex**  ---------- ---------- ---------- ---------- ---------- ---------- TAATTTTAGA TGTAACTCAA CATGTGAATA TGTGATGGCT

**Harrington**  ---------- ---------- ---------- ---------- ---------- ---------- TAATTTTAGA TGTAACTCAA CATGTGAATA TGTGATGGCT

**HA52**  ---------- ---------- ---------- ---------- ---------- ---------- TAATTTTAGA TGTAACTCAA CATGTGAATA TGTGATGGCT

**z043**  ---------- ---------- ---------- ---------- ---------- ---------- TAATTTTAGA TGTAACTCAA CATGTGAATA TGTGATGGCT

**L47**  ---------- ---------- ---------- ---------- ---------- ---------- TAATTTTAGA TGTAACTCAA CATGTGAATA TGTGATGGCC

**Strider**  ---------- ---------- ---------- ---------- ---------- ---------- TAATTTTAGA TGTAACTCAA CATGTGAATA TGTGATGGCC

**W127**  ---------- ---------- ---------- ---------- ---------- ---------- TAATTTTAGA TGTAACTCAA CATGTGAATA TGTGATGGCC

**Adorra**  TAACTAGATA CATTCATATC TAAACAAATC TAAGACAAGA ATTTTGAAAC GGAGGTAGTA TAATTTTCGA TGTAACTCAA CATGTGAATA TGTGATGGCC

**Haruna Nijo**  ---------- ---------- ---------- ---------- ---------- ---------- TAATTTTAGA TGTAACTCAA CATGTGAATA TGTGATGGCC

**L35**  ---------- ---------- ---------- ---------- ---------- ---------- TAATTTTAGA TGTAACTCAA CATGTGAATA TGTGATCGCC

**L48**  ---------- ---------- ---------- ---------- ---------- ---------- TAATTTTAGA TGTAACTCAA CATGTGAATA TGTGATGGCC

**L68**  ---------- ---------- ---------- ---------- ---------- ---------- TAATTTTAGA TGTAACTCAA CATGTGAATA TGTGATCGCC

**PI296897**  ---------- ---------- ---------- ---------- ---------- ---------- TAATTTTAGA TGTAACTCAA CATGTGAATA TGTGATGGCC

**AB75**  ---------- ---------- ---------- ---------- ---------- ---------- TAATTTTAGA TGTAACTCAA CATGTGAATA TGTGATGGCC

**m279**  TAACTAGATA CATTCATATC TAAACAAATC TAAGACAAGA ATTTTGAAAC GGAGGTAGTA TAATTTTCGA TGTAACTCAA CATGTGAATA TGTGATGGCC

**Legacy**  TAACTAGATA CATTCATATC TAAACAAATC TAAGACAAGA ATTTTGGAAC GGAGGTAGTA TAATTTTCGA TGTAACTCAA CATGTGAATA TGTGATGGCC

**Orca**  TAACTAGATA CATTCATATC TAAACAAATC TAAGACAAGA ATTTTGGAAC GGAGGTAGTA TAATTTTCGA TGTAACTCAA CATGTGAATA TGTGATGGCC

**Tango**  TAACTAGATA CATTCATATC TAAACAAATC TAAGACAAGA ATTTTGGAAC GGAGGTAGTA TAATTTTCGA TGTAACTCAA CATGTGAATA TGTGATGGCC

**UC958**  TAACTAGATA CATTCATATC TAAACAAATC TAAGACAAGA ATTTTGGAAC GGAGGTAGTA TAATTTTCGA TGTAACTCAA CATGTGAATA TGTGATGGCC

**UC960**  TAACTAGATA CATTCATATC TAAACAAATC TAAGACAAGA ATTTTGGAAC GGAGGTAGTA TAATTTTCGA TGTAACTCAA CACGTGAATA TGTGATGGCC

**Steptoe**  TAACTAGATA CATTCATATC TAAACAAATC TAAGACAAGA ATTTTGGAAC GGAGGTAGTA TAATTTTCGA TGTAACTCAA CATGTGAATA TGTGATGGCC

**Stander**  TAACTAGATA CATTCATATC TAAACAAATC TAAGACAAGA ATTTTGGAAC GGAGGTAGTA TAATTTTCGA TGTAACTCAA CATGTGAATA TGTGATGGCC

**Ashqelon**  ---------- ---------- ---------- ---------- ---------- ---------- TAATTTTAGA TGTAACTCAA CATGTGAATA TGTGATGGCC

**L46**  ---------- ---------- ---------- ---------- ---------- ---------- TAATTTTAGA TGTAACTCAA CATGTGAATA TGTGATGGCC

**....|....| ....|....| ....|....| ....|....| ....|....| ....|....| ....|....| ....|....| ....|....| ....|....|**

**2810 2820 2830 2840 2850 2860 2870 2880 2890 2900**

**Morex**  AAGTCACGAT CTACATTTAG AAAAGTTTTT TCAAGAAACA AACACCCAAA TGAAAAGTGA TTCTTAAAGG AAAAAAGTGC ATGGATAAAA TGGCAGTTTC

**Harrington**  AAGTCACGAT CTACATTTAG AAAAGTTTTT TCAAGAAACA AACACCCAAA TGAAAAGTGA TTCTTAAAGG AAAAAAGTGC ATGGATAAAA TGGCAGTTTC

**HA52**  AAGTCACGAT CTACATTTAG AAAAGTTTTT TCAAGAAACA AACACCCAAA TGAAAAGTGA TTCTTAAAGG AAAAAAGTGC ATGGATAAAA TGGCAGTTTC

**z043**  AAGTCACGAT CTACATTTAG AAAAGTTTTT TCAAGAAACA AACACCCAAA TGAAAAGTGA TTCTTAAAGG AAAAAAGTGC ATGGATAAAA TGGCAGTTTC

**L47**  AAGTCACGAT CTACATTTAG AAAAGTTTTT TCAAGAAA-- ---------- ---------- ---------- ------GTGC ATGGATAAAA TGGCAGTTTC

**Strider**  AAGTCACGAT CTACATTTAG AAAAGTTTTT TCAAGAAACA AACACCCAAA TGAAAAGTGA TTCTTAAAGG AAAAAAGTGC ATGGATAAAA TGGCAGTTTC

**W127**  AAGTCACGAT CTACATTTAG AAAAGTTTTT TCAAGAAACA AACACCCAAA TGAAAAGTGA TTCTTAAAGG AAAAAAGTGC ATGGATAAAA TGGCAGTTTC

**Adorra**  AAGTCACGAT CTACATTTAG AAAAGTTTTT TCAAGAAACA AACACCCAAA TGAAAAGTGA TTCTTAAAGG AAAAAAGTGC ATGGATAAAA TGGCAGTTTC

**Haruna Nijo**  AAGTCACGAT CTACATTTAG AAAAGTTTTT TCAGGAAACA AACACCCAAA TGAAAAATGA TTCTTAAAGG AAAAAAGTGC ATGGATAAAA TGGCAGTTTC

**L35**  AAGTCACGAT CTACATTTAG AAAAGTTTTT TCAAGAAACA AACACCCAAA TGAAAAGTGA TTCTTAAAGG AAAAAAGTGC ATGGATAAAA TGGCAGTTTC

**L48**  AAGTCACGAT CTACATTTAG AAAAGTTTTT TCAAGAAACA AACACCCAAA TGAAAAGTGA TTCTTAAAGG AAAAAAGTGC ATGGATAAAA TGGCAGTTTC

**L68**  AAGTCACGAT CTACATTTAG AAAAGTTTTT TCAAGAAACA AACACCCAAA TGAAAAGTGA TTCTTAAAGG AAAAAAGTGC ATGGATAAAA TGGCAGTTTC

**PI296897**  AAGTCACGAT CTACATTTAG AAAAGTTTTT TCAAGAAA-- ---------- ---------- ---------- ------GTGC ATGGATAAAA TGGCAGTTTC

**AB75**  AAGTCACGAT CTGCATTTAG AAAAGTTTTT TCAAGAAACA AACACCCAAA TGAAAAGTGA TTCTTAAAGG AAAAAAGTGC ATGGATAAAA TGGCAGTTTC

**m279**  AAGTCACGAT CTACATTTAG AAAAGTTTTT TCAAGAAACA AACACCCAAA TGAAAAGTGA TTCTTAAAGG AAAAAAGTGC ATGGATAAAA TGGCAGTTTC

**Legacy**  AAGTCACGAT CTACATTTAG AAAAGCTTTT -CAAGAAACA AACACCCAAA TGAAAAGTGA TTCTTAAAGG AAAAAAGTGC ATGGATAAAA TGGCAGTTTC

**Orca**  AAGTCACGAT CTACATTTAG AAAAGCTTTT -CAAGAAACA AACACCCAAA TGAAAAGTGA TTCTTAAAGG AAAAAAGTGC ATGGATAAAA TGGCAGTTTC

**Tango**  AAGTCACGAT CTACATTTAG AAAAGCTTTT -CAAGAAACA AACACCCAAA TGAAAAGTGA TTCTTAAAGG AAAAAAGTGC ATGGATAAAA TGGCAGTTTC

**UC958**  AAGTCACGAT CTACATTTAG AAAAGCTTTT -CAAGAAACA AACACCCAAA TGAAAAGTGA TTCTTAAAGG AAAAAAGTGC ATGGATAAAA TGGCAGTTTC

**UC960**  AAGTCACGAT CTACATTTAG AAAAGCTTTT -CAAGAAACA AACACCCAAA TGAAAAGTGA TTCTTAAAGG AAAAAAGTGC ATGGATAAAA TGGCAGTTTC

**Steptoe**  AAGTCACGAT CTACATTTAG AAAAGCTTTT -CAAGAAACA AACACCCAAA TGAAAAGTGA TTCTTAAAGG AAAAAAGTGC ATGGATAAAA TGGCAGTTTC

**Stander**  AAGTCACGAT CTACATTTAG AAAAGCTTTT -CAAGAAACA AACACCCAAA TGAAAAGTGA TTCTTAAAGG AAAAAAGTGC ATGGATAAAA TGGCAGTTTC

**Ashqelon**  AAGTCACGAT CTACATTTAG AAAAGTTTTT TCAAGAAACA AACACCCAAA TGAAAAGTGA TTCTTAAAGG AAAAAAGTGC ATGGATAAAA TGGCAGTTTC

**L46**  AAGTCACGAT CTACATTTAG AAAAGTTTTT TCAAGAAACA AACACCCAAA TGAAAAGTGA TTCTTAAAGG AAAAAAGTGC ATGGATAAAA TGGCAGTTTC

**....|....| ....|....| ....|....| ....|....| ....|....| ....|....| ....|....| ....|....| ....|....| ....|....|**

**2910 2920 2930 2940 2950 2960 2970 2980 2990 3000**

**Morex**  AGATTAGGAC AAGGCGTGGT AAACCTGACT TGATCATTTC TGTTACCCAA TATAGCCCCG CCCAATTTTT GTATTTTCTT ATTCCTCCCA AATAAGACAT

**Harrington**  AGATTAGGAC AAGGCGTGGT AAACCTGACT TGATCATTTC TGTTACCCAA TATAGCCCCG CCCAATTTTT GTATTTTCTT ATTCCTCCCA AATAAGACAT

**HA52**  AGATTAGGAC AAGGCGTGGT AAACCTGACT TGATCATTTC TGTTACCCAA TATAGCCCCG CCCAATTTTT GTATTTTCTT ATTCCTCCCA AATAAGACAT

**z043**  AGATTAGGAC AAGGCGTGGT AAACCTGACT TGATCATTTC TGTTACCCAA TATAGCCCCG CCCAATTTTT GTATTTTCTT ATTCCTCCCA AATAAGACAT

**L47**  AGATTAGGAC AAGGCGTGGT AAACCTGACT TGATCATTTC TGTTACCCAA TATACCCCCG CCCAATTTTT GTTTTTTCTT ATTCCTCCCA AATAAGACAT

**Strider**  AGATTAGGAC AAGGCGTGGT AAACCTGACT TGATCATTTC TGTTACCCAA TATAGCCCCG CCCAATTTTT GTATTTTCTT ATTCCTCCCA AATAAGACAT

**W127**  AGATTAGGAC AAGGCGTGGT AAACCTGACT TGATCATTTC TGTTACCCAA TATACCCCCG CCCAATTTTT GTTTTTTCTT ATTCCTCCCA AATAAGACAT

**Adorra**  AGATTAGGAC AAGGCGTGGT AAACCTGACT TGATCATTTC TGTTACCCAA TATACCCCCG CCCAATTTTT GTATTTTCTT ATTCCTCCCA AATAAGACAT

**Haruna Nijo**  AGATTAGGAC AAGGCGTGGT AAACCTGACT TGATCATTTC TGTTACCCAA TATACCCCCG CCCAATTTTT GTTTTTTCTT ATTCCTCCCA AATAAGACAT

**L35**  AGATTAGGAC AAGGCGTGGT AAACCTGACT TGATCATTTC TGTTACCCAA TATACCCCCG CCCAATTTTT GTTTTTTCTT ATTCCTCCCA AATAAGACAT

**L48**  AGATTAGGAC AAGGCGTGGT AAACCTGACT TGATCATTCC TGTTACCCAA TATACCCCCG CCCAATTTTT GTTTTTTCTT ATTCCTCCCA AATAAGACAT

**L68**  AGATTAGGAC AAGGCGTGGT AAACCTGACT TGATCATTTC TGTTACCCAA TATACCCCCG CCCAATTTTT GTTTTTTCTT ATTCCTCCCA AATAAGACAT

**PI296897**  AGATTAGGAC AAGGCGTGGT AAACCTGACT TGATCATTTC TGTTACCCAA TATACCCCCG CCCAATTTTT GTTTTTTCTT ATTCCTCCCA AATAAGACAT

**AB75**  AGATTAGGAC AAGGCGTGGT AAACATGACT TGATCATTTC AGTTACCCAA TAC-CCCCAG CCCAATTTTT ATTTTTTCTT ATTCCTCCCA AATAAGACAT

**m279**  AGATTAGGAC AAGGCGTGGT AAACCTGACT TGATCATTTC TGTTACCCAA TATACCCCCG CCCAATTTTT GTATTTTCTT ATTCCTCCCA AATAAGACAT

**Legacy**  AGATTAGGAC AAGGCGTGGT AAACCTGACT TGATCATTTC TGTTACCCAA TATACCCCCG CCCAATTTTT GTATTTTCTT ATTCCTCCCA AATAAGACAT

**Orca**  AGATTAGGAC AAGGCGTGGT AAACCTGACT TGATCATCTC TGTTACCCAA TATACCCCCG CCCAATTTTT GTATTTTCTT ATTCCTCCCA AATAAGACAT

**Tango**  AGATTAGGAC AAGGCGTGGT AAACCTGACT TGATCATTTC TGTTACCCAA TATACCCCCG CCCAATTTTT GTATTTTCTT ATTCCTCCCA AATAAGACAT

**UC958**  AGATTAGGAC AAGGCGTGGT AAACCTGACT TGATCATTTC TGTTACCCAA TATACCCCCG CCCAATTTTT GTATTTTCTT ATTCCTCCCA AATAAGACAT

**UC960**  AGATTAGGAC AAGGCGTGGT AAACCTGACT TGATCATTTC TGTTACCCAA TATACCCCCG CCCAATTTTT GTATTTTCTT ATTCCTCCCA AATAAGACAT

**Steptoe**  AGATTAGGAC AAGGCGTGGT AAACCTGACT TGATCATTTC TGTTACCCAA TATACCCCCG CCCAATTTTT GTATTTTCTT ATTCCTCCCA AATAAGACAT

**Stander**  AGATTAGGAC AAGGCGTGGT AAACCTGACT TGATCATTTC TGTTACCCAA TATACCCCCG CCCAATTTTT GTATTTTCTT ATTCCTCCCA AATAAGACAT

**Ashqelon**  AGATTAGGAC AAGGCGTGGT AAACCTGACT TGATCATTTC TGTTACCCAA TATACCCCCG CCCAATTTTT GTTTTTTCTT ATTCCTCCCA AATAAGACAT

**L46**  AGATTAGGAC AAGGCGTGGT AAACCTGACT TGATCATTTC TGTTACCCAA TATACCCCCG CCCAATTTTT GTTTTTTCTT ATTCCTCCCA AATAAGACAT

**....|....| ....|....| ....|....| ....|....| ....|....| ....|....| ....|....| ....|....| ....|....| ....|....|**

**3010 3020 3030 3040 3050 3060 3070 3080 3090 3100**

**Morex**  CATATAAACA GGAAAATGAT AT-TTAAAAT ATGAGAAATA TATAGGATTC ATCTGTGCAA CTTAAATACT TAAAATGAT- TTTTTTATTG GAAAAGACTA

**Harrington**  CATATAAACA GGAAAATGAT AT-TTAAAAT ATGAGAAATA TATAGGATTC ATCTGTGCAA CTTAAATACT TAAAATGAT- TTTTTTATTG GAAAAGACTA

**HA52**  CATATAAACA GGAAAATGAT AT-TTAAAAT ATGAGAAATA TATAGGATTC ATCTGTGCAA CTTAAATACT TAAAATGAT- TTTTT-ATTG GAAAAGACTA

**z043**  CATATAAACA GGAAAATGAT AT-TTAAAAT ATGAGAAATA TATAGGATTC ATCTGTGCAA CTTAAATACT TAAAATGAT- TTTTTTATTG GAAAAGACTA

**L47**  CATATAAACT TGACACATTC GTATTACAAT ATGTGAAATA TATAGGATTT ATCTGTGCAA CTTAAATACT TAAATTGACA TTTTTTATTG GAAAAGACTA

**Strider**  CATATAAATA GGAAAATGAT AT-TTAAAAT ATGAGAAATA TATAGGATTC ATCTGTGCAA CTTAAATACT TAAAATGAT- TTTTTTATTG GAAAAGACTA

**W127**  CATATAAACT TGACACATTC GTATTACAAT ATGTGAAATA TATAGGATTT ATCTGTGCAA CTTAAATACT TAAATTGACA TTTTTTATTG GAAAAGACTA

**Adorra**  CATATAAACA GGAAAATGAT AT-TTAAAAT ATGAGAAATA TATAGGATTC ATCTGTGCAA CTTAAATACT TAAAATGA-- TTTTTTATTG GAAAAGACTA

**Haruna Nijo**  CATATAAACT TGACACATTC GTATTACAAT ATGTGAAATA TATAGGATTT ATCTTTGCAA CTTAAATACT TAAATTGACC TTTTTTATTG GAAAAGACTA

**L35**  CATATAAACT TGACACATTC GTATTACAAT ATGTGAAATA TATAGGATTT ATCTGTGCAA CTTAAATACT TAAATTGACA TTTTTTATTG GAAAAGACTA

**L48**  CATATAAACT TGACACATTC GTATTACAAT ATGTGAAATA TATAGGATTT ATCTGTGCAA CTTAAATACT TAAATTGACA TTTTTTATTG GAAAAGACTA

**L68**  CATATAAACT TGACACATTC GTATTACAAT ATGTGAAATA TATAGGATTT ATCTGTGCAA CTTAAATACT TAAATTGACA TTTTTTATTG GAAAAGACTA

**PI296897**  CATATAAACT TGACACATTC GTATTACAAT ATGTGAAATA TATAGGATTT ATCTGTGCAA CTTAAATACT TAAATTGACA TTTTTTATTG GAAAAGACTA

**AB75**  CATATAAACT TGGCACATTC GTATTACAAT ATGTGAAATA TATAGGATTT ATCTGTGCAA CTTAAATACT TAAAATGACA TTTTTTATTG GAAAAGACTA

**m279**  CATATAAACA GGAAAATGAT AT-TTAAAAT ATGAGAAATA TATAGGATTC ATCTGTGCAA CTTAAATACT TAAAATGA-- TTTTTTATTG GAAAAGACTA

**Legacy**  CATATAAACA GGAAAATGGT AT-TTAAAAT ATGAGAAATA TATAGGATTC ATCTGTGCAA CTTAAATACT TAAAATGA-- TTTTTTATTG GAAAAGACTA

**Orca**  CATATAAACA GGAAAATGGT AT-TTAAAAT ATGAGAAATA TATAGGATTC ATCTGTGCAA CTTAAATACT TAAAATGA-- TTTTTTATTG GAAAAGACTA

**Tango**  CATATAAACA GGAAAATGGT AT-TTAAAAT ATGAGAAATA TATAGGATTC ATCTGTGCAA CTTAAATACT TAAAATGA-- TTTTTTATTG GAAGAGACTA

**UC958**  CATATAAACA GGAAAATGGT AT-TTAAAAT ATGAGAAATA TATAGGATTC ATCTGTGCAA CTTAAATACT TAAAATGA-- TTTTTTATTG GAAAAGACTA

**UC960**  CATATAAACA GGAAAATGGT AT-TTAAAAT ATGAGAAATA TATAGGATTC ATCTGTGCAA CTTAAATACT TAAAATGA-- TTTTTTATTG GAAAAGACTA

**Steptoe**  CATATAAACA GGAAAATGGT AT-TTAAAAT ATGAGAAATA TATAGGATTC ATCTGTGCAA CTTAAATACT TAAAATGA-- TTTTTTATTG GAAAAGACTA

**Stander**  CATATAAACA GGAAAATGGT AT-TTAAAAT ATGAGAAATA TATAGGATTC ATCTGTGCAA CTTAAATACT TAAAATGA-- TTTTTTATTG GAAAAGACTA

**Ashqelon**  CATATAAACT TGACACATTC GTATTACAAT ATGTGAAATA TATAGGATTT ATCTGTGCAA CTTAAATACT TAAATTGACA TTTTTTATTG GAAAAGACTA

**L46**  CATATAAACT TGACACATTC GTATTACAAT ATGTGAAATA TATAGGATTT ATCTGTGCAA CTTAAATACT TAAATTGACA TTTTTTATTG GAAAAGACTA

**....|....| ....|....| ....|....| ....|....| ....|....| ....|....| ....|....| ....|....| ....|....| ....|....|**

**3110 3120 3130 3140 3150 3160 3170 3180 3190 3200**

**Morex**  ATTTTATATA TTTATGGTAC ACCA-AAAAT CCAAAATGTT TTCGGCACAT TGTAGTCTCT ATGATTCATT GACCCCACAC GTGCGGTTCC CTCACGTCTA

**Harrington**  ATTTTATATA TTTATGGTAC ACCA-AAAAT CCAAAATGTT TTCGGCACAT TGTAGTCTCT ATGATTCATT GACCCCACAC GTGCGGTTCC CTCACGTCTA

**HA52**  ATTTTATATA TTTATGGTAC ACCACAAAAT CCAAAATGTT TT-GGCACAT TGTAGTCTCT ATGATTCATT GACCCCACAC GTGCGGTTCC CTCACGTCTA

**z043**  ATTTTATATA TTTATGGTAC ACCA-AAAAT CCAAAATGTT TTCGGCACAT TGTAGTCTCT ATGATTCATT GACCCCACAC GTGCGGTTCC CTCACGTCTA

**L47**  ATTTTATATA TTTATGGTAC ACCA-AAAAT CCAAAATGTT TTCGGCACAT TGTAGTCTCT ATGATTCATT GACCCCACAC GTGCGGTTCC CTCAGGCCTA

**Strider**  ATTTTATATA TTTATGGTAC ACCA-AAAAT CCAAAATGTT TTCGGCACAT TGTAGTCTCT ATGATTCATT GACCCCACAC GTGCGGTTCC CTCACGTCTA

**W127**  ATTTTATATA TTTATGGTAC ACCA-AAAAT CCAAAATGTT TTCGGCACAT TGTAGTCTCT ATGATTCATT GACCCCACAT GTGCGGTTCC CTCAGGCCTA

**Adorra**  ATTTTATATA TTTATGGTAC ACCA-AAAAT CCAAAATGTT TTCGGCACAT GGTAGTCTCT ATGATTCATT GACCCCACAC GTGCGGTTCC CTCACGTCTA

**Haruna Nijo**  ATTTTATATA TTTATGGTAC ACCA-AAAAT CCAAAATGTT TTCGGCACAT TGTAGTCTCT ATGATTCATT GACCCCACAC GTGCGGTTCC CTCAGGCCTA

**L35**  ATTTTATATA TTTATGGTAC ACCA-AAAAT CCAAAATGTT TTCGGCACAT TGTAGTCTCT ATGATTCATT GACCCCACAC GTGCGGTTCC CTCAGGCCTA

**L48**  ATTTTATATA TTTATGGTAC ACCA-AAAAT CCAAAATGTT TTCGGCACAT TGTAGTCTCT ATGATTCATT GACCCCACAC GTGCGGTTCC CTCAGGCCTA

**L68**  ATTTTATATA TTTATGGTAC ACCG-AAAAT CCAAAATGTT TTCGGCACAT TGTAGTCTCT ATGATTCATT GACCCCACAC GTGCGGTTCC CTCAGGCCTA

**PI296897**  ATTTTATATA TTTATGGTAC ACCA-AAAAT CCAAAATGTT TTCGGCACAT TGTAGTCTCT ATGATTCATT GACCCCACAC GTGCGGTTCC CTCAGGCCTA

**AB75**  ATTTTATATA TTTATGGTAC ACCA-AAAAT CCAAAATGTT TTCGGCACAT TGTAGTCTCT ATGATTCATT GACCCCGCAT GTGCGGTTCC CTCAGACCTA

**m279**  ATTTTATATA TTTATGGTAC ACCA-AAAAT CCAAAATGTT TTCGGCACAT GGTAGTCTCT ATGATTCATT GACCCCACAC GTGCGGTTCC CTCACGTCTA

**Legacy**  ATTTTATATA TTTATGGTAC ACCA-AAAAT CCAAAATGTT TTCGGCACAT TGTAGTCTCT ATGATTCATT GACCCCACAC GTGCGGTTCC CTCACGTCTA

**Orca**  ATTTTATATA TTTATGGTAC ACCA-AAAAT CCAAAATGTT TTCGGCACAT TGTAGTCTCT ATGATTCATT GACCCCACAC GTGCGGTTCC CTCACGTCTA

**Tango**  ATTTTATATA TTTATGGTAC ACCA-AAAAT CCAAAATGTT TTCGGCACAT TGTAGTCTCT ATGATTCATT GACCCCACAC GTGCGGTTCC CTCACGTCTA

**UC958**  ATTTTATATA TTTATGGTAC ACCA-AAAAT CCAAAATGTT TTCGGCACAT TGTAGTCTCT ATGATTCATT GACCCCACAC GTGCGGTTCC CTCACGTCTA

**UC960**  ATTTTATATA TTTATGGTAC ACCA-AAAAT CCAAAATGTT TTCGGCACAT TGTAGTCTCT ATGATTCATT GACCCCACAC GTGCGGTTCC CTCACGTCTA

**Steptoe**  ATTTTATATA TTTATGGTAC ACCA-AAAAT CCAAAATGTT TTCGGCACAT TGTAGTCTCT ATGATTCATT GACCCCACAC GTGCGGTTCC CTCACGTCTA

**Stander**  ATTTTATATA TTTATGGTAC ACCA-AAAAT CCAAAATGTT TTCGGCACAT TGTAGTCTCT ATGATTCATT GACCCCACAC GTGCGGTTCC CTCACGTCTA

**Ashqelon**  ATTTTATATA TTTATGGTAC ACCA-AAAAT CCAAAATGTT TTCGGCACAT TGTAGTCTCT ATGATTCATT GACCCCACAT GTGCGGTTCC CTCAGGCCTA

**L46**  ATTTTATATA TTTATGGTAC ACCA-AAAAT CCAAAATGTT TTCGGCACAT TGTAGTCTCT ATGATTCATT GACCCCACAT GTGCGGTTCC CTCAGGCCTA

**....|....| ....|....| ....|....| ....|....| ....|....| ....|....| ....|....| ....|....| ....|....| ....|....|**

**3210 3220 3230 3240 3250 3260 3270 3280 3290 3300**

**Morex**  AACGTGGA-- ---------C TGTAAATTTA AATTTTTAAA TTGAGTGTCT TGGGTCTTGA ATTTAAGACC TTTTGACTCG GATACCATGT AAAACTGCAC

**Harrington**  AACGTGGA-- ---------C TGTAAATTTA AATTTTTAAA TTGAGTGTCT TGGGTCTTGA ATTTAAGACC TTTTGACTCG GATACCATGT AAAACTGCAC

**HA52**  AACGTGGA-- ---------C TGTAAATTTA AATTTTTAAA TTGAGTGTCT TGGGTCTTGA ATTTAAGACC TTTTGACTCG GATACCATGT AAAACTGCAC

**z043**  AACGTGGA-- ---------C TGTAAATTTA AATTTTTAAA TTGAGTGTCT TGGGTCTTGA ATTTAAGACC TTTTGACTCG GATACCATGT AAAACTGCAC

**L47**  AACGTGGATT GAAAGTAGGC TGCAATTTTA AAATTTTAAA TTTAGTGTGT CAGGTCTTGA ATTTAAGATC TTTTGACTCG GATACCATGT AAAACTGCAC

**Strider**  AACGTGGA-- ---------C TGTAAATTTA AATTTTTAAA TTGAGTGTCT TGGGTCTTGA ATTTAAGACC TTTTGACTCG GATACCATGT AAAACTGCAC

**W127**  AACGTGGATT GAAAGTAGGC TGCAATTTTA AAATTTTAAA TTTAGTGTGT CAGGTCTTGA ATTTAAGATC TTTTGACTCG GATACCATGT AAAACTGCAC

**Adorra**  AACGTGGA-- ---------C TGTAAATTTA AATTTTTAAA TTGAGTGTCT TGGGTCTTGA ATTTAAGACC TTTTGACTCG GATACCATGT AAAACTGCAC

**Haruna Nijo**  AACGTGGATT GAAAGTAGGC TGCAATTTTA AAATTTTAAA TTTAGTGTGT CAGGTCTTGA ATTTAAGATC TTTTGACTCG GATACCATGT AAAACTGCAC

**L35**  AACGTGGATT GAAAGTAGGC TGCAATTTTA AAATTTTAAA TTTAGTGTGT CAGGTCTTGA ATTTAAGATC TTTTGACTCG GATACCATGT AAAACTGCAC

**L48**  AACGTGGATT GAAAGTAGGC TGCAATTTTA AAATTTTAAA TTTAGTGTGT CAGGTCTTGA ATTTAAGATC TTTTGACTCG GATACCATGT AAAACTGCAC

**L68**  AACGTGGATT GAAAGTAGGC TGCAATTTTA AAATTTTAAA TTTAGTGTGT CAGGTCTTGA ATTTAAGATC TTTTGACTCG GATACCATGT AAAACTGCAC

**PI296897**  AACGTGGATT GAAAGTAGGC TGCAATTTTA AAATTTTAAA TTTAGTGTGT CAGGTCTTGA ATTTAAGATC TTTTGACTCG GATACCATGT AAAACTGCAC

**AB75**  AACGTGGATT GAAAGTAGGC TGCAATTTTA AAATTTTAAA TTTAATGTGT CAGGTCTTGA ATTTAAGATC TTTTGACTCG GATACCATGT AAAACTGCAC

**m279**  AACGTGGA-- ---------C TGTAAATTTA AATTTTTAAA TTGAGTGTCT TGGGTCTTGA ATTTAAGACC TTTTGACTCG GATACCATGT AAAACTGCAC

**Legacy**  AACGTGGA-- ---------C TGTAAATTTA AATTTTTAAA TTGAGTGTCT TGGGTCTTGA ATTTAAGACC TTTTGACTCG GATACCATGT AAAACTGCAC

**Orca**  AACGTGGA-- ---------C TGTAAATTTA AATTTTTAAA TTGAGTGTCT TGGGTCTTGA ATTTAAGACC TTTTGACTCG GATACCATGT AAAACTGCAC

**Tango**  AACGTGGA-- ---------C TGTAAATTTA AATTTTTAAA TTGAGTGTCT TGGGTCTTGA ATTTAAGACC TTTTGACTCG GATACCATGT AAAACTGCAC

**UC958**  AACGTGGA-- ---------C TGTAAATTTA AATTTTTAAA TTGAGTGTCT TGGGTCTTGA ATTTAAGACC TTTTGACTCG GATACCATGT AAAACTGCAC

**UC960**  AACGTGGA-- ---------C TGTAAATTTA AATTTTTAAA TTGAGTGTCT TGGGTCTTGA ATTTAAGACC TTTTGACTCG GATACCATGT AAAACTGCAC

**Steptoe**  AACGTGGA-- ---------C TGTAAATTTA AATTTTTAAA TTGAGTGTCT TGGGTCTTGA ATTTAAGACC TTTTGACTCG GATACCATGT AAAACTGCAC

**Stander**  AACGTGGA-- ---------C TGTAAATTTA AATTTTTAAA TTGAGTGTCT TGGGTCTTGA ATTTAAGACC TTTTGACTCG GATACCATGT AAAACTGCAC

**Ashqelon**  AACGTGGATT GAAAGTAGGC TGCAATTTTA AAATTTTAAA TTTAGTGTGT CAGGTCTTGA ATTTAAGATC TTTTGACTCG GATACCATGT AAAACTGCAC

**L46**  AACGTGGATT GAAAGTAGGC TGCAATTTTA AAATTTTAAA TTTAGTGTGT CAGGTCTTGA ATTTAAGATC TTTTGACTCG GATACCATGT AAAACTGCAC

**....|....| ....|....| ....|....| ....|....| ....|....| ....|....| ....|....| ....|....| ....|....| ....|....|**

**3310 3320 3330 3340 3350 3360 3370 3380 3390 3400**

**Morex**  GCACCAGACA GTTCACCCAT AAGCTCTTGC TTATGG---- ---------- -------ATA CTTCAACAAT AAGAATAGTG AGGTAGCACA AACCTTGA-T

**Harrington**  GCACCAGACA GTTCACCCAT AAGCTCTTGC TTATGG---- ---------- -------ATA CTTCAACAAT AAGAATAGTG AGGTAGCACA AACCTTGA-T

**HA52**  GCACCAGACA GTTCACCCAT AAGCTCTTGC TTATGG---- ---------- -------ATA CTTCAACAAT AAGAATAGTG AGGTAGCACA AACCTTGA-T

**z043**  GCACCAGACA GTTCACCCAT AAGCTCTTGC TTATGG---- ---------- -------ATA CTTCAACAAT AAGAATAGTG AGGTAGCACA AACCTTGA-T

**L47**  ----CAGACA GTTCACC-AT AAGCTCTTGC TTATGGGGAA AGGTGGGCTA TGCATTTATA CTTCAACAAT AAAAATAGTG ATTTAGCACA AACCTTGA-T

**Strider**  GCACCAGACA GTTCACCCAT AAGCTCTTGC TTATGG---- ---------- -------ATA CTTCAACAAT AAGAATAGTG AGGTAGCACA AACCTTGA-T

**W127**  ----CAGACA GTTCACCCAT AAGCTCTTGC TTATGGGGAA AGGTGGGCTA TGCATTTATA CTTCAACAAT AAAAATAGTG ATTTAGCACA AACCTTGA-T

**Adorra**  GCACAAGACA GTTCACCCAT AAGCTCTTGC TTATGGAGAA AGGTGGGCTA TGCATTTATA CTTCAACAAT AAGAATAGTG AGGTAGCACA AACCTTGAAT

**Haruna Nijo**  ----CAGACA GTTCACCCAT AAGCTCTTGC TTATGGGGAA AGGTGGGCTA TGCATTTATA CTTCAACAAT AAAAATAGTG ATTTAGCACA AACCTTGA-T

**L35**  ----CAGACA GTTCACCCAT AAGCTCTTGC TTATGGGGAA AGGTGGGCTA TGCATTTATA CTTCAACAAT AAAAATAGTG ATTTAGCACA AACCTTGA-T

**L48**  ----CAGACA GTTCACCCAT AAGCTCTTGC TTATGGGGAA AGGTGGGCTA TGCATTTATA CTTCAACAAT AAAAATAGTG ATTTAGCACA AACCTTGA-T

**L68**  ----CAGACA GTTCACCCAT AAGCTCTTGC TTATGGGGAA AGGTGGGCTA TGCATTTATA CTTCAACAAT AAAAATAGTG ATTTAGCACA AACCTTGA-T

**PI296897**  ----CAGACA GTTCACC-AT AAGCTCTTGC TTATGGGGAA AGGTGGGCTA TGCATTTATA CTTCAACAAT AAAAATAGTG ATTTAGCACA AACCTTGA-T

**AB75**  ----CAGACA GTTCACCCAT AAGCTCTTGC TTATGGGGAA AGGTGGGCTA TGCATTTATA CTTCAACAAT AAAAATAGTG ATTTAGCACA AACCTTGA-T

**m279**  GCACAAGACA GTTCACCCAT AAGCTCTTGC TTATGGAGAA AGGTGGGCTA TGCATTTATA CTTCAACAAT AAGAATAGTG AGGTAGCACA AACCTTGA-T

**Legacy**  GCACCAGACA GTTCACCCAT AAGCTCTTGC TTATGGAGAA AGGTGGGCTA TGCATTTATA CTTCAACAAT AAGAATAGTG AGGTAGCACA AACCTTGA-T

**Orca**  GCACCAGACA GTTCACCCAT AAGCTCTTGC TTATGGAGAA AGGTGGGCTA TGCATTTATA CTTCAACAAT AAGAATAGTG AGGTAGCACA AACCTTGA-T

**Tango**  GCACCAGACA GTTCACCCAT AAGCTCTTGC TTATGGAGAA AGGTGGGCTA TGCATTTATA CTTCAACAAT AAGAATAGTG AGGTAGCACA AACCTTGA-T

**UC958**  GCACCAGACA GTTCACCCAT AAGCTCTTGC TTATGGAGAA AGGTGGGCTA TGCATTTATA CTTCAACAAT AAGAATAGTG AGGTAGCACA AACCTTGA-T

**UC960**  GCACCAGACA GTTCACCCAT AAGCTCTTGC TTATGGAGAA AGGTGGGCTA TGCATTTATA CTTCAACAAT AAGAATAGTG AGGTAGCACA AACCTTGA-T

**Steptoe**  GCACCAGACA GTTCACCCAT AAGCTCTTGC TTATGGAGAA AGGTGGGCTA TGCATTTATA CTTCAACAAT AAGAATAGTG AGGTAGCACA AACCTTGA-T

**Stander**  GCACCAGACA GTTCACCCAT AAGCTCTTGC TTATGGAGAA AGGTGGGCTA TGCATTTATA CTTCAACAAT AAGAATAGTG AGGTAGCACA AACCTTGA-T

**Ashqelon**  ----CAGACA GTTCACCCAT AAGCTCTTGC TTATGGGGAA AGGTGGGCTA TGCATTTATA CTTCAACAAT AAAAATAGTG ATTTAGCACA AACCTTGA-T

**L46**  ----CAGACA GTTCACCCAT AAGCTCTTGC TTATGGGGAA AGGTGGGCTA TGCATTTATA CTTCAACAAT AAAAATAGTG ATTTAGCACA AACCTTGA-T

**....|....| ....|....| ....|....| ....|....| ....|....| ....|....| ....|....| ....|....| ....|....| ....|....|**

**3410 3420 3430 3440 3450 3460 3470 3480 3490 3500**

**Morex**  GCAAGGCTAG ATGACACTGA TGTGTGTGTG TGTT-GGGGG GGGGGGGGGG G-------AT GCACCTGAA- TCCGATTTCA GGAAGTTTGG CACTACTTTT

**Harrington**  GCAAGGCTAG ATGACACTGA TGTGTGTGTG TGTT-GGGGG GGGGGGGGGG GG------AT GCACCTGAA- TCCGATTTCA GGAAGTTTGG CACTACTTTT

**HA52**  GCAAGGCTAG ATGACACTGA TGTGCCATTT TGTT-GGGGG GGGGGGGGGG GG------AT GCACCTGAA- TCCGATTTCA GGAAGTTTGG CACTACTCTT

**z043**  GCAAGGCTAG ATGACACAGA TGTGTG--TG TGTT-GGGGG GGGGGGGGG- --------AT GCACCTGAA- TCCGATTTCA GGAAGTTTGG CACTACTTTT

**L47**  GCAAGGCTAG ATGACACAGA TGTGTGTGTG TGTGTGGGGG GGGGGGGGGG GGGGG-TTA- GCACCTGAA- TCCGAGTTCA GGAAGTTTGG CACTACTTTT

**Strider**  GCAAGGCTAG ATGACACTGA TGTGTGTGTG TGTT--GGGG GGGGGGGGGG GG-------- --ACCTGAA- TCCGATTTCA GGAAGTTTGG CACTACTTTT

**W127**  GCAAGGCTAG ATGACACAGA TGTGTGTGTG TG--GGGGGG GGGGGGGGGG GG----TGAT GCACCTGAA- TCCGAGTTCA GGAAGTTTGG CACTACTTTT

**Adorra**  GCAAGGCTAG ATGACACAGA TGGGTGTGTG TGTTGGGGGG GGGGGGG--- --------AT GCACCTGAAA TCCGATTTCA GGAAGTTTGG CACTACTTTT

**Haruna Nijo**  GCAAGGCTAG ATGACACAGA TGTGTGTGTG TGTGTGTGTG GGGGGGGGGG GGGGGGTGAT GCACCTGAA- TCCGAGCTCA GGAAGTTTGG CACTACTTTT

**L35**  GCAAGGCTAG ATGACACAGA TGTGTGTGTG TGTGTGGGGG GGGGGGGGGG GGGGG-TTA- GCACCTGAA- TCCGAGTTCA GGAAGTTTGG CACTACTTTT

**L48**  GCAAGGCTAG ATGACACAGA TGTGTGTGTG TGTGTGGGGG GGGGGGGG-- --------AT GCACCTGAA- TCCGATTTCA GGAAGTTTGG CACTACTTTT

**L68**  GCAAGGCTAG ATGACACAGA TGTGTGTGTG TGTGTGGGGG GGGGGGGGGG GGGGG-TTA- GCACCTGAA- TCCGAGTTCA GGAAGTTTGG CACTACTTTT

**PI296897**  GCAAGGCTAG ATGACACAGA TGTGTGTGTG TGTGTGGGGG GGGGGGGGGG GGGGG-TTA- GCACCTGAA- TCCGAGTTCA GGAAGTTTGG CACTACTTTT

**AB75**  GCAAGGCTAG ATGACACAGA TGTGTGTGTG TGGGTGGGGG GGGGGGGG-- ---------- -CACCTGAA- TCCGAGTTCA GGAAGTTTGG CACTACTTTT

**m279**  GCAAGGCTAG ATGACACAGA TGGGTGTGTG TGTT-GGGGG GGGGGGG--- --------AT GCACCTGAAA TCCGATTTCA GGAAGTTTGG CACTACTTTT

**Legacy**  GCAAGGCTAG ATGACACAGA TGGGTGTGTG TGTT-GGGGG GGGGGGGG-- --------AT GCACCTGAA- TCCGATTTCA GGAAGTTTGG CACTACTTTT

**Orca**  GCAAGGCTAG ATGACACAGA TGGGTGTGTG TGTT-GGGGG GGGGGGGGG- --------AT GCACCTGAA- TCCGATTTCA GGAAGTTTGG CACTACTTTT

**Tango**  GCAAGGCTAG ATGACACAGA TGGGTGTGTG TGTT-GGGGG GGGGGGGGGG G-------AT GCACCTGAA- TCCGATTTCA GGAAGTTTGG CACTACTTTT

**UC958**  GCAAGGCTAG ATGACACAGA TGGGTGTGTG TGTT-GGGGG GGGGGGGG-- --------AT GCACCTGAA- TCCGATTTCA GGAAGTTTGG CACTACTTTT

**UC960**  GCAAGGCTAG ATGACACAGA TGGGTGTGTG TGTT-GGGGG GGGGGGGGGG --------AT GCACCTGAA- TCCGATTTCA GGAAGTTTGG CACTACTTTT

**Steptoe**  GCAAGGCTAG ATGACACAGA TGGGTGTGTG TGTT-GGGGG GGGGGGGGG- --------AT GCACCTGAA- TCCGATTTCA GGAAGTTTGG CACTACTTTT

**Stander**  GCAAGGCTAG ATGACACAGA TGGGTGTGTG TGTT-GGGGG GGGGGGGG-- --------AT GCACCTGAA- TCCGATTTCA GGAAGTTTGG CACTACTTTT

**Ashqelon**  GCAAGGCTAG ATGACACAGA TGTGTGTGTG TG---GGGGG GGGGGGGGGG GGG---TGAT GCACCTGAA- TCCGAGTTCA GGAAGTTTGG CACTACTTTT

**L46**  GCAAGGCTAG ATGACACAGA TGTGTGTGTG TG---GGGGG GGGGGGGGGG GGG---TGAT GCACCTGAA- TCCGAGTTCA GGAAGTTTGG CACTACTTTT

**....|....| ....|....| ....|....| ....|....| ....|....| ....|....| ....|....| ....|....| ....|....| ....|....|**

**3510 3520 3530 3540 3550 3560 3570 3580 3590 3600**

**Morex**  GCCTTCCGGG AGACCATGTA TGCTATTG-T TTGAGCAAAG TATCATAGCA AATACAAGAC CATCTTAAAA TACATGACAT GAAATAGTTA AAACAAATGC

**Harrington**  GCCTTCCGGG AGACCATGTA TGCTATTG-T TTGAGCAAAG TATCATAGCA AATACAAGAC CATCTTAAAA TACATGACAT GAAATAGTTA AAACAAATGC

**HA52**  GCCTTCCGGG AGACCATGTA TGCTATTG-T TTGAGCAAAG TATCATAGCA AATACAAGAC CATCTTAAAA TACATGACAT GAAATAGTTA AAACAAATGC

**z043**  GCCTTCCGGG AGACCATGTA TGCTATTG-T TTGAGCAAAG TATCATAGCA AATACAAGAC CATCTTAAAA TACATGACAT GAAATAGTTA AAACAAATGC

**L47**  GCCTTCCGGG AAACCATATA TGTTATTGCT TTGAGCAAAG TATCATAGCA AATACAAGAC CTCCTTAAAA TACATGACAT GAAATAGTTA AAACAAATGC

**Strider**  GCCTTCCGGG AGACCATGTA TGCTATTA-T TTGAGCAAAG TATCATAGCA AATACAAGAC CATCTTAAAA TACATGACAT GAAATAGTTA AAACAAATGC

**W127**  GCCTTCCGGG AGACCATATA TGTTATTGCT TTGAGCAAAG TATCATAGCA AATACAAGAC CTTCTTAAAA TACATGACAT GAAATAGTTA AAACAAATGC

**Adorra**  GCCTTCCGGG AGACCATGTA TGCTATTGTT TTGAGCAAAG TATCATAGCA AATACAAGAC CATCTTAAAA TACATGACAT GAAATAGTTA AAACAAATGC

**Haruna Nijo**  CCCTTCCGGG AGACCATATA TGTTATTGCT TTGAGCAAAG TATCATAGCA AATACAAGAC CTTCTTAAAA TACATGACAT GAAATAGTTA AAACAAATGC

**L35**  GCCTTCCGGG AAACCATATA TGTTATTGCT TTGAGCAAAG TATCATAGCA AATACAAGAC CTCCTTAAAA TACATGACAT GAAATAGTTA AAACAAATGC

**L48**  GCCTTCCGGG AGACCATGTA TGCTATTG-T TTGAGCAAAG TATCATAGCA AATACAAGAC CATCTTAAAA TACATGACAT GAAATAGTTA AAACAAATGC

**L68**  GCCTTCCGGG AAACCATATA TGTTATTGCT TTGAGCAAAG TATCATAGCA AATACAAGAC CTCCTTAAAA TACATGACAT GAAATAGTTA AAACAAATGC

**PI296897**  GCCTTCCGGG AAACCATATA TGTTATTGCT TTGAGCAAAG TATCATAGCA AATACAAGAC CTCCTTAAAA TACATGACAT GAAATAGTTA AAACAAATGC

**AB75**  GCCTTCCGGG AGACCATACA TGTTATTGCT TTGAGCAAAG TATCATAGCA AATACAAGAC CTTCTTAAAA TACATGACAT GAAATAGTTA AAACAAATGC

**m279**  GCCTTCCGGG AGACCATGTA TGCTATTGTT TTGAGCAAAG TATCATAGCA AATACAAGAC CATCTTAAAA TACATGACAT GAAATAGTTA AAACAAATGC

**Legacy**  GCCTTCCGGG AGACCATGTA TGCTATTGTT T-GAGCAAAG TATCATAGCA AATACAAGAC CATCTTAAAA TACATGACAT GAAATAGTTA AAACAAATGC

**Orca**  GCCTTCCGGG AGACCATGTA TGCTATTGTT T-GAGCAAAG TATCATAGCA AATACAAGAC CATCTTAAAA TACATGACAT GAAATAGTTA AAACAAATGC

**Tango**  GCCTTCCGGG AGACCATGTA TGCTATTGTT T-GAGCAAAG TATCATAGCA AATACAAGAC CATCTTAAAA TACATGACAT GAAATAGTTA AAACAAATGC

**UC958**  GCCTTCCGGG AGACCATGTA TGCTATTGTT T-GAGCAAAG TATCATAGCA AATACAAGAC CATCTTAAAA TACATGACAT GAAATAGTTA AAACAAATGC

**UC960**  GCCTTCCGGG AGACCATGTA TGCTATTGTT T-GAGCAAAG TATCATAGCA AATACAAGAC CATCTTAAAA TACATGACAT GAAATAGTTA AAACAAATGC

**Steptoe**  GCCTTCCGGG AGACCATGTA TGCTATTGTT T-GAGCAAAG TATCATAGCA AATACAAGAC CATCTTAAAA TACATGACAT GAAATAGTTA AAACAAATGC

**Stander**  GCCTTCCGGG AGACCATGTA TGCTATTGTT T-GAGCAAAG TATCATAGCA AATACAAGAC CATCTTAAAA TACATGACAT GAAATAGTTA AAACAAATGC

**Ashqelon**  GCCTTCCGGG AGACCATATA TGTTATTGCT TTGAGCAAAG TATCATAGCA AATACAAGAC CTTCTTAAAA TACATGACAT GAAATAGTTA AAACAAATGC

**L46**  GCCTTCCGGG AGACCATATA TGTTATTGCT TTGAGCAAAG TATCATAGCA AATACAAGAC CTTCTTAAAA TACATGACAT GAAATAGTTA AAACAAATGC

**....|....| ....|....| ....|....| ....|....| ....|....| ....|....| ....|....| ....|....| ....|....| ....|....|**

**3610 3620 3630 3640 3650 3660 3670 3680 3690 3700**

**Morex**  ACGATAATAT ATACCATTAC CATTACAGAA AAAATGGCTC TACTCGTACT TAACTGTTTG AACTAATAGT ACAAATAAAA ATAAAATTGC AGTGCTATGA

**Harrington**  ACGATAATAT ATACCATTAC CATTACAGAA AAAATGGCTC TACTCGTACT TAACTGTTTG AACTAATAGT ACAAATAAAA ATAAAATTGC AGTGCTATGA

**HA52**  ACGATAATAT ATACCATTAC CATTACAGAA AAAATGGCTC TACTCGTACT TAACTGTTTG AACTAATAGT ACAAATAAAA ATAAAATTGC AGTGCTATGA

**z043**  ACGATAATAT ATACCATTAC CATTACAGAA AAAATGGCTC TACTCGTACT TAACTGTTTG AACTAATAGT ACAAATAAAA ATAAAATTGC AGTGCTATGA

**L47**  ACGATAATAT ATACCATTGC CATTACAGAA AAA-TGGCTC TACTAGTACT TAACTGTGTG AAATAATAGT ACAAATAAAA ATAAAATTGC AGTGCTATGA

**Strider**  ACGATAATAT ATACCATTAC CATTACAGAA AAAATGGCTC TACTCGTACT TAACTGTTTG AACTAATAGT ACAAATAAAA ATAAAATTGC AGTGCTATGA

**W127**  A-GATAATAT ATACCATTGC CATTACAGAA AAA-TGGCTC TACT------ TAACTGTTTG AACTAATAGT ACAAATAAAA ATAAAATTGC AGTGCTATGA

**Adorra**  ACGATAATAT ATACCATTAC CATTACAGAA AAAATGGCTC TACTCGTACT TAACTGTTTG AACTAATAGT ACAAATAAAA ATTAAATTGC AGTGCTATGA

**Haruna Nijo**  ACGATAATAT ATACCATTGC CATTACAGAA AAA-TGGCTC TACT------ TAACTGTTTG AACTAATAGT ACAAATAAAA ATAAAATTGC AGTGCTATGA

**L35**  ACGATAATAT ATACCATTGC CATTACAGAA AAA-TGGCTC TACT------ TAACTGTTTG AACTAATAGT ACAAATAAAA ATAAAATTGC AGTGCTATGA

**L48**  ACGATAATAT ATACCATTAC CATTACAGAA AAAATGGCTC TACTAGTACT TATATGTGTG AAATAATAGT ACAAATAAAA ATAAAATTGC AGTGCTATGA

**L68**  ACGATAATAT ATACCATTGC CATTACAGAA AAA-TGGCTC TACT------ TAACTGTTTG AACTAATAGT ACAAATAAAA ATAAAATTGC AGTGCTATGA

**PI296897**  ACGATAATAT ATACCATTGC CATTACAGAA AAA-TGGCTC TACT------ TAACTGTTTG AACTAATAGT ACAAATAAAA ATAAAATTGC AGTGCTATGA

**AB75**  ACGATAATAT ATACCATTGC CATTACAGAA AAA-TGGCTC TACC------ TAACTGTTTG AACTAATAGT ACAAATAAAA ATAAAATTGC AGTGCTATGA

**m279**  ACGATAATAT ATACCATTAC CATTACAGAA AAAATGGCTC TACTCGTACT TAACTGTTTG AACTAATAGT ACAAATAAAA ATTAAATTGC AGTGCTATGA

**Legacy**  ACGATAATAT ATACCATTAC CATTACAGAA AAAATGGCTC TACTCGTACT TAACTGTTTG AACTAATAGT ACAAATAAAA ATAAAATTGC AGTGCTATGA

**Orca**  ACGATAATAT ATACCATTAC CATTACAGAA AAAATGGCTC TACTCGTACT TAACTGTTTG AACTAATAGT ACAAATAAAA ATAAAATTGC AGTGCTATGA

**Tango**  ACGATAATAT ATACCATTAC CATTACAGAA AAAATGGCTC TACTCGTACT TAACTGTTTG AACTAATAGT ACAAATAAAA ATAAAATTGC AGTGCTATGA

**UC958**  ACGATAATAT ATACCATTAC CATTACAGAA AAAATGGCTC TACTCGTACT TAACTGTTTG AACTAATAGT ACAAATAAAA ATAAAATTGC AGTGCTATGA

**UC960**  ACGATAATAT ATACCATTAC CATTACAGAA AAAATGGCTC TACTCGTACT TAACTGTTTG AACTAATAGT ACAAATAAAA ATAAAATTGC AGTGCTATGA

**Steptoe**  ACGATAATAT ATACCATTAC CATTACAGAA AAAATGGCTC TACTCGTACT TAACTGTTTG AACTAATAGT ACAAATAAAA ATAAAATTGC AGTGCTATGA

**Stander**  ACGATAATAT ATACCATTAC CATTACAGAA AAAATGGCTC TACTCGTACT TAACTGTTTG AACTAATAGT ACAAATAAAA ATAAAATTGC AGTGCTATGA

**Ashqelon**  A-GATAATAT ATACCATTGC CATTACAGAA AAA-TGGCTC TACT------ TAACTGTTTG AACTAATAGT ACAAATAAAA ATAAAATTGC AGTGCTATGA

**L46**  ACGATAATAT ATACCATTGC CATTACAGAA AAA-TGGCTC TACT------ TAACTGTTTG AACTAATAGT ACAAATAAAA ATAAAATTGC AGTGCTATGA

**....|....| ....|....| ....|....| ....|....| ....|....| ....|....| ....|....| ....|....| ....|....| ....|....|**

**3710 3720 3730 3740 3750 3760 3770 3780 3790 3800**

**Morex**  TAAATACCTA CAAGCAGACT TCAAAGCAGC AGCAGCGGCG GTTGGCCATC CTGAGTGGGA ATTTCCTAAC GATGTCGGGC AGTACAATGA CACTCCCGAG

**Harrington**  TAAATACCTA CAAGCAGACT TCAAAGCAGC AGCAGCGGCG GTTGGCCATC CTGAGTGGGA ATTTCCTAAC GATGTCGGGC AGTACAATGA CACTCCCGAG

**HA52**  TAAATACCTA CAAGCAGACT TCAAAGCAGC AGCAGCGGCG GTTGGCCATC CTGAGTGGGA ATTTCCTAAC GATGTCGGGC AGTACAATGA CACTCCCGAG

**z043**  TAAATACCTA CAAGCAGACT TCAAAGCAGC AGCAGCGGCG GTTGGCCATC CTGAGTGGGA ATTTCCTAAC GATGTCGGAC AGTACAATGA CACTCCCGAG

**L47**  TAAATACATA CAAGCAGACT TCAAAGCAGC AGCAGCGGCG GTTGGCCATC CTGAGTGGGA ATTTCCTAAC GATGTCGGAC AGTACAATGA CACTCCCGAG

**Strider**  TAAATACCTA CAAGCAGACT TCAAAGCAGC AGCAGCGGCG GTTGGCCATC CTGAGTGGGA ATTTCCTAAC GATGTCGGAC AGTACAATGA CACTCCCGAG

**W127**  TAAATACCTA CAAGCAGACT TCAAAGCAGC AGCAGCGGCG GTCGGCCATC CTGAGTGGGA ATTTCCTAAC GATGCCGGAC AGTACAATGA CACTCCCGAG

**Adorra**  TAAATACCTA CAAGCAGACT TCAAAGCAGC AGCAGCGGCG GTTGGCCATC CTGAGTGGGA ATTTCCTAAC GATGTCGGAC AGTACAATGA CACTCCCGAG

**Haruna Nijo**  TAAATACCTA CAAGCAGACT TCAAAGCAGC AGCAGCGGCG GTCGGCCATC CTGAGTGGGA ATTTCCTAAC GATGCCGGAC AGTACAATGA CACTCCCGAG

**L35**  TAAATACCTA CAAGCAGACT TCAAAGCAGC AGCAGCGGCG GTCGGCCATC CTGAGTGGGA ATTTCCTAAC GATGCCGGAC AGTACAATGA CACTCCCGAG

**L48**  TAAATACCTA CAAGCAGACT TCAAAGCAGC AGCAGCGGCG GTCGGCCATC CTGAGTGGGA ATTTCCTAAC GATGCCGGAC AGTACAATGA CACTCCCGAG

**L68**  TAAATACCTA CAAGCAGACT TCAAAGCAGC AGCAGCGGCG GTCGGCCATC CTGAGTGGGA ATTTCCTAAC GATGCCGGAC AGTACAATGA CACTCCCCAG

**PI296897**  TAAATACCTA CAAGCAGACT TCAAAGCAGC AGCAGCGGCG GTCGGCCATC CTGAGTGGGA ATTTCCTAAC GATGCCGGAC AGTACAATGA CACTCCCGAG

**AB75**  TAAATACCTA CAAGCAGACT TCAAAGCAGC GGCAGCGGCG GTTGGCCATC CTGAGTGGGA ATTTCCTAAC GATGCCGGAC AGTACAATGA CACTCCCGAG

**m279**  TAAATACCTA CAAGCAGACT TCAAAGCAGC AGCAGCGGCG GTTGGCCATC CTGAGTGGGA ATTTCCTAAC GATGTCGGAC AGTACAATGA CACTCCCGAG

**Legacy**  TAAATACCTA CAAGCAGACT TCAAAGCAGC AGCAGCGGCG GTTGGCCATC CTGAGTGGGA ATTTCCTAAC GATGTCGGAC AGTACAATGA CACTCCCGAG

**Orca**  TAAATACCTA CAAGCAGACT TCAAAGCAGC AGCAGCGGCG GTTGGCCATC CTGAGTGGGA ATTTCCTAAC GATGTCGGAC AGTACAATGA CACTCCCGAG

**Tango**  TAAATACCTA CAAGCAGACT TCAAAGCAGC AGCAGCGGCG GTTGGCCATC CTGAGTGGGA ATTTCCTAAC GATGTCGGAC AGTACAATGA CACTCCCGAG

**UC958**  TAAATACCTA CAAGCAGACT TCAAAGCAGC AGCAGCGGCG GTTGGCCATC CTGAGTGGGA ATTTCCTAAC GATGTCGGAC AGTACAATGA CACTCCCGAG

**UC960**  TAAATACCTA CAAGCAGACT TCAAAGCAGC AGCAGCGGCG GTTGGCCATC CTGAGTGGGA ATTTCCTAAC GATGTCGGAC AGTACAATGA CACTCCCGAG

**Steptoe**  TAAATACCTA CAAGCAGACT TCAAAGCAGC AGCAGCGGCG GTTGGCCATC CTGAGTGGGA ATTTCCTAAC GATGTCGGAC AGTACAATGA CACTCCCGAG

**Stander**  TAAATACCTA CAAGCAGACT TCAAAGCAGC AGCAGCGGCG GTTGGCCATC CTGAGTGGGA ATTTCCTAAC GATGTCGGAC AGTACAATGA CACTCCCGAG

**Ashqelon**  TAAATACCTA CAAGCAGACT TCAAAGCAGC AGCAGCGGCG GTCGGCCATC CTGAGTGGGA ATTTCCTAAC GATGCCGGAC AGTACAATGA CACTCCCGAG

**L46**  TAAATACCTA CAAGCAGACT TCAAAGCAGC AGCAGCGGCG GTCGGCCATC CTGAGTGGGA ATTTCCTAAC GATGCCGGAC AGTACAATGA CACTCCCGAG

**....|....| ....|....| ....|....| ....|....| ....|....| ....|....| ....|....| ....|....| ....|....| ....|....|**

**3810 3820 3830 3840 3850 3860 3870 3880 3890 3900**

**Morex**  AGAACTCAAT TCTTCAGAGA CAACGGGACA TACCTAAGTG AGAAGGGGAG GTTTTTCCTT GCATGGTACT CCAACAATCT GATCAAGCAC GGTGACAGGA

**Harrington**  AGAACTCAAT TCTTCAGAGA CAACGGGACA TACCTAAGTG AGAAGGGGAG GTTTTTCCTT GCATGGTACT CCAACAATCT GATCAAGCAC GGTGACAGGA

**HA52**  AGAACTCAAT TCCTCAGAGA CAACGGGACA TACCTAAGTG AGAAGGGGAG GTTTTTCCTT GCATGGTACT CCAACAATCT GATCAAGCAC GGTGACAGGA

**z043**  AGAACTCAAT TCTTCAGAGA CAACGGGACA TACCTAAGTG AGAAGGGGAG GTTTTTCCTT GCATGGTACT CCAACAATCT GATCAAGCAC GGTGACAGGA

**L47**  AGAACTCAAT TCTTCAGAGA CAACGGGACA TACCTAAGTG AGAAGGGGAG GTTTTTCCTT GCATGGTACT CCAACAATCT GATCAAGCAC GGTGACAGGA

**Strider**  AGAACTCAAT TCTTCAGAGA CAACGGGACA TACCTAAGTG AGAAGGGGAG GTTTTTCCTT GCATGGTACT CCAACAATCT GATCAAGCAC GGTGACAGGA

**W127**  AGAACTCAAT TCTTCAGAGA CAACGGGACA TACCTAAGTG AGAAGGGGAG GTTTTTCCTT GCATGGTACT CCAACAATCT GATCAAGCAC GGTGACAGGA

**Adorra**  AGAACTCAAT TCTTCAGAGA CAACGGGACA TACCTAAGTG AGAAGGGGAG GTTTTTCCTT GCATGGTACT CCAACAATCT GATCAAGCAC GGTGACAGGA

**Haruna Nijo**  AGAACTCAAT TCTTCAGGGA CAACGGGACA TACCTAAGTG AGAAGGGGAG GTTTTTCCTT GCATGGTACT CCAACAATCT GATCAAGCAC GGTGACAGGA

**L35**  AGAACTCAAT TCTTCAGGGA CAACGGGACA TACCTAAGTG AGAAGGGGAG GTTTTTCCTT GCATGGTACT CCAACAATCT GATCAAGCAC GGTGACAGGA

**L48**  AGAACTCAAT TCTTCAGGGA CAACGGGACA TACCTAAGTG AGAAGGGGAG GTTTTTCCTT GCATGGTACT CCAACAATCT GATCAAGCAC GGTGACAGGA

**L68**  AGAACTCAAT TCCTCAGGGA CAACGGGACA TTCCTAAGTG AGAAGGGGAA GTTTTTCCTT GCATGGTACT CCAACAATCT GATCAAGCAC GGTGACAGGA

**PI296897**  AGAACTCAAT TCTTCAGGGA CAACGGGACA TACCTAAGTG AGAAGGGGAG GTTTTTCCTT GCATGGTACT CCAACAATCT GATCAAGCAC GGTGACAGGA

**AB75**  AGAACTCAAT TCTTCAGGGA CAACGGGACA TACCTCACTG AGAAGGGGAG GTTTTTCCTT GCATGGTACT CCAACAATCT GATCAAGCAC GGTGACAGGA

**m279**  AGAACTCAAT TCTTCAGAGA CAACGGGACA TACCTAAGTG AGAAGGGGAG GTTTTTCCTT GCATGGTACT CCAACAATCT GATCAAGCAC GGTGACAGGA

**Legacy**  AGAACTCAAT TCTTCAGAGA CAACGGGACA TACCTAAGTG AGAAGGGGAG GTTTTTCCTT GCATGGTACT CCAACAATCT GATCAAGCAC GGTGACAGGA

**Orca**  AGAACTCAAT TCTTCAGAGA CAACGGGACA TACCTAAGTG AGAAGGGGAG GTTTTTCCTT GCATGGTACT CCAACAATCT GATCAAGCAC GGTGACAGGA

**Tango**  AGAACTCAAT TCTTCAGAGA CAACGGGACA TACCTAAGTG AGAAGGGGAG GTTTTTCCTT GCATGGTACT CCAACAATCT GATCAAGCAC GGTGACAGGA

**UC958**  AGAACTCAAT TCTTCAGAGA CAACGGGACA TACCTAAGTG AGAAGGGGAG GTTTTTCCTT GCATGGTACT CCAACAATCT GATCAAGCAC GGTGACAGGA

**UC960**  AGAACTCAAT TCTTCAGAGA CAACGGGACA TACCTAAGTG AGAAGGGGAG GTTTTTCCTT GCATGGTACT CCAACAATCT GATCAAGCAC GGTGACAGGA

**Steptoe**  AGAACTCAAT TCTTCAGAGA CAACGGGACA TACCTAAGTG AGAAGGGGAG GTTTTTCCTT GCATGGTACT CCAACAATCT GATCAAGCAC GGTGACAGGA

**Stander**  AGAACTCAAT TCTTCAGAGA CAACGGGACA TACCTAAGTG AGAAGGGGAG GTTTTTCCTT GCATGGTACT CCAACAATCT GATCAAGCAC GGTGACAGGA

**Ashqelon**  AGAACTCAAT TCTTCAGGGA CAACGGGACA TACCTAAGTG AGAAGGGGAG GTTTTTCCTT GCATGGTACT CCAACAATCT GATCAAGCAC GGTGACAGGA

**L46**  AGAACTCAAT TCTTCAGGGA CAACGGGACA TACCTAAGTG AGAAGGGGAG GTTTTTCCTT GCATGGTACT CCAACAATCT GATCAAGCAC GGTGACAGGA

**....|....| ....|....| ....|....| ....|....| ....|....| ....|....| ....|....| ....|....| ....|....| ....|....|**

**3910 3920 3930 3940 3950 3960 3970 3980 3990 4000**

**Morex**  TCTTGGATGA AGCAAACAAG GTCTTCTTGG GATACAAGGT GCAACTGGCA ATCAAGGTAT AAACACTTCC ATGCATCCTA AAGATCTCGG CTTATTACTA

**Harrington**  TCTTGGATGA AGCAAACAAG GTCTTCTTGG GATACAAGGT GCAACTGGCA ATCAAGGTAT AAACACTTCC ATGCATCCTA AAGATCTCGG CTTATTACTA

**HA52**  TCTTGGATGA AGCAAACAAG GTCTTCTTGG GATACAAGGT GCAACTGGCA ATCAAGGTAT AAACACTTCC ATGCATCCTA AAGATCTCGG CTTATTACTA

**z043**  TCTTGGATGA AGCAAACAAG GTCTTCTTGG GATACAAGGT GCAACTGGCA ATCAAGGTAT AAACACTTCC ATGCATCCTA AAGATCTCGG CTTATTACTA

**L47**  TCTTGGATGA AGCAAACAAG GTCTTCTTGG GATACAAGGT GCAACTGGCA ATCAAGGTAT AAACACTTCC ATGCATCCTA AAGATCTCGG CTTATTACTA

**Strider**  TCTTGGATGA AGCAAACAAG GTCTTCTTGG GATACAAGGT GCAACTGGCA ATCAAGGTAT AAACACTTCC ATGCATCCTA AAGATCTCGG TTTATTACTA

**W127**  TCTTGGATGA AGCAAACAAG GTCTTCTTGG GATACAAGGT GCAACTGGCA ATCAAGGTAT AAACACTTCC ATGCATCCTA AAGATCTCGG CTTATTACTA

**Adorra**  TCTTGGATGA AGCAAACAAG GTCTTCTTGG GATACAAGGT GCAACTGGCA ATCAAGGTAT AAACACTTCC ATGCATCCTA AAGATCTCGG TTTATTACTA

**Haruna Nijo**  TCTTGGATGA AGCAAACAAG GTCTTCTTGG GATACAAGGT GCAATTGGCA ATCAAGGTAT AAGCACTTTC ATGCCTCCTA AAGATCTCGG TTTATTACTA

**L35**  TCTTGGATGA AGCAAACAAG GTCTTCTTGG GATACAAGGT GCAACTGGCA ATCAAGGTAT AAGCACTTTC ATGCCTCCTA AAGATCTCGG TTTATTACTA

**L48**  TCTTGGATGA AGCAAACAAG GTCTTCTTGG GATACAAGGT GCAACTGGCA ATCAAGGTAT AAACACTTCC ATGCATCCTA AAGATCTCGG TTTATTACTA

**L68**  TCTTGGATGA AGCAAACAAG GTCTTCTTGG GATACAAGGT GCAACTGGCA ATCAAGGTAT AAGCACTTTC ATGCCTCCTA AAGATCTCGG TTTATTACTA

**PI296897**  TCTTGGATGA AGCAAACAAG GTCTTCTTGG GATACAAGGT GCAACTGGCA ATCAAGGTAT AAGCACTTTC ATGCCTCCTA AAGATCTCGG TTTATTACTA

**AB75**  TCTTGGACGA AGCAAACAAG GTCTTCTTGG GATACAAGGT GCAACTGGCA ATCAAGGTAT AAGCACTTTC ATGCCTCCTA AAGATCTCGG TTTATTACTA

**m279**  TCTTGGATGA AGCAAACAAG GTCTTCTTGG GATACAAGGT GCAACTGGCA ATCAAGGTAT AAACACTTCC ATGCATCCTA AAGATCTCGG TTTATTACTA

**Legacy**  TCTTGGATGA AGCAAACAAG GTCTTCTTGG GATACAAGGT GCAACTGGCA ATCAAGGTAT AAACACTTCC ATGCATCCTA AAGATCTCGG TTTATTACTA

**Orca**  TCTTGGATGA AGCAAACAAG GTCTTCTTGG GATACAAGGT GCAACTGGCA ATCAAGGTAT AAACACTTCC ATGCATCCTA AAGATCTCGG TTTATTACTA

**Tango**  TCTTGGATGA AGCAAACAAG GTCTTCTTGG GATACAAGGT GCAACTGGCA ATCAAGGTAT AAACACTTCC ATGCATCCTA AAGATCTCGG TTTATTACTA

**UC958**  TCTTGGATGA AGCAAACAAG GTCTTCTTGG GATACAAGGT GCAACTGGCA ATCAAGGTAT AAACACTTCC ATGCATCCTA AAGATCTCGG TTTATTACTA

**UC960**  TCTTGGATGA AGCAAACAAG GTCTTCTTGG GATACAAGGT GCAACTGGCA ATCAAGGTAT AAACACTTCC ATGCATCCTA AAGATCTCGG TTTATTACTA

**Steptoe**  TCTTGGATGA AGCAAACAAG GTCTTCTTGG GATACAAGGT GCAACTGGCA ATCAAGGTAT AAACACTTCC ATGCATCCTA AAGATCTCGG TTTATTACTA

**Stander**  TCTTGGATGA AGCAAACAAG GTCTTCTTGG GATACAAGGT GCAACTGGCA ATCAAGGTAT AAACACTTCC ATGCATCCTA AAGATCTCGG TTTATTACTA

**Ashqelon**  TCTTGGATGA AGCAAACAAG GTCTTCTTGG GATACAAGGT GCAACTGGCA ATCAAGGTAT AAGCACTTTC ATGCCTCCTA AAGATCTCGG TTTATTACTA

**L46**  TCTTGGATGA AGCAAACAAG GTCTTCTTGG GATACAAGGT GCAACTGGCA ATCAAGGTAT AAACACTTCC ATGCATCCTA AAGATCTCGG TTTATTACTA

**....|....| ....|....| ....|....| ....|....| ....|....| ....|....| ....|....| ....|....| ....|....| ....|....|**

**4010 4020 4030 4040 4050 4060 4070 4080 4090 4100**

**Morex**  CAGTAGTAGA TAGGATTTGA GAAACCATGG TTCAGTTGAG AAGTTGTGTA TGATAAACAA CAACAAAAAT ACACAAACTA TCCAGGCTAA GGGAACTCGC

**Harrington**  CAGTAGTAGA TAGGATTTGA GAAACCATGG TTCAGTTGAG AAGTTGTGTA TGATAAACAA CAACAAAAAT ACACAAACTA TCCAGGCTAA GGGAACTCGC

**HA52**  CAGTAGTAGA TAGGATTTGA GAAACCATGG TTCAGTTGAG AAGTTGTGTA TGATAAACAA CAACAAAAAT ACACAAACTA TCCAGGCTAA GGGAACTCGC

**z043**  CAGTAGTAGA TAGGATTTGA GAAACCATGG TTCAGTTGAG AAGTTGTGTA TGATAAACAA CAACAAAAAT ACACAAACTA TCCAGGCTAA GGGAACTCGC

**L47**  CAGTAGTAGA TAGGATTTGA GAAACCATGG TTCAGTTGAG AAGTTGTGTA TGATAAACAA CAACAAAAAT ACACAAACTA TCCAGGCTAA GGGAACTCGC

**Strider**  CAGTAGTAGA TAGGATTTGA GAAACCATGA TTCAGTTGAG AAGTTGTGTA TGATAAACAA CAACAAAAAT ACACAAACTA TCCAGGCTAA GGGAACTCGC

**W127**  CAGTAGTAGA TAGGATTTGA GAAACCATGG TTCAGTTGAG AAGTTGTGTA TGATAAACAA CAACAAAAAT ACACAAACTA TCCAGGCTAA GGGAACTCGC

**Adorra**  CAGTAGTAGA TAGGATTTGA GAAACCATGA TTCAGTTGAG AAGTTGTGTA TGATAAACAA CAACAAAAAT ACACAAACTA TCCAGGCTAA GGGAACTCGC

**Haruna Nijo**  CAGTAGTAGA TAGGATTTGA GAAACCATGA TTCAGTTGAG AAGTTGTGTA TGATAAACAA CAAAAAAAAT ACACAAACTA TCCAGGCTAA GGGAACTCGC

**L35**  CAGTAGTAGA TAGGATTTGA GAAACCATGA TTCAGTTGAG AAGTTGTGTA TGATAAACAA CAAAAAAAAT ACACAAACTA TCCAGGCTAA GGGAACTCGC

**L48**  CAGTAGTAGA TAGGATTTGA GAAACCATGA TTCAGTTGAG AAGTTGTGTA TGATAAACAA CAACAAAAAT ACACAAACTA TCCAGGCTAA GGGAACTCGC

**L68**  CAGTAGTAGA TAGGATTTGA GAAACCATGA TTCAGTTGAG AAGTTGTGTA TGATAAACAA CAACAAAAAT ACACAAACTA TCCAGGCTAA GGGAACTCGC

**PI296897**  CAGTAGTAGA TAGGATTTGA GAAACCATGA TTCAGTTGAG AAGTTGTGTA TGATAAACAA CAAAAAAAAT ACACAAACTA TCCAGGCTAA GGGAACTCGC

**AB75**  CAGTAGTAGA TAGGATTTGA GAAACCATGA TTCAGTTGAG AAGTTGTGTA TGATAAACAA CAACAAAAAC ACACAAACTA TCCAGGCTAA GGGAACTCGC

**m279**  CAGTAGTAGA TAGGATTTGA GAAACCATGA TTCAGTTGAG AAGTTGTGTA TGATAAACAA CAACAAAAAT ACACAAACTA TCCAGGCTAA GGGAACTCGC

**Legacy**  CAGTAGTAGA TAGGATTTGA GAAACCATGA TTCAGTTGAG AAGTTGTGTA TGATAAACAA CAACAAAAAT ACACAAACTA TCCAGGCTAA GGGAACTCGC

**Orca**  CAGTAGTAGA TAGGATTTGA GAAACCATGA TTCAGTTGAG AAGTTGTGTA TGATAAACAA CAACAAAAAT ACACAAACTA TCCAGGCTAA GGGAACTCGC

**Tango**  CAGTAGTAGA TAGGATTTGA GAAACCATGA TTCAGTTGAG AAGTTGTGTA TGATAAACAA CAACAAAAAT ACACAAACTA TCCAGGCTAA GGGAACTCGC

**UC958**  CAGTAGTAGA TAGGATTTGA GAAACCATGA TTCAGTTGAG AAGTTGTGTA TGATAAACAA CAACAAAAAT ACACAAACTA TCCAGGCTAA GGGAACTCGC

**UC960**  CAGTAGTAGA TAGGATTTGA GAAACCATGA TTCAGTTGAG AAGTTGTGTA TGATAAACAA CAACAAAAAT ACACAAACTA TCCAGGCTAA GGGAACTCGC

**Steptoe**  CAGTAGTAGA TAGGATTTGA GAAACCATGA TTCAGTTGAG AAGTTGTGTA TGATAAACAA CAACAAAAAT ACACAAACTA TCCAGGCTAA GGGAACTCGC

**Stander**  CAGTAGTAGA TAGGATTTGA GAAACCATGA TTCAGTTGAG AAGTTGTGTA TGATAAACAA CAACAAAAAT ACACAAACTA TCCAGGCTAA GGGAACTCGC

**Ashqelon**  CAGTAGTAGA TAGGATTTGA GAAACCATGA TTCAGTTGAG AAGTTGTGTA TGATAAACAA CAAAAAAAAT ACACAAACTA TCCAGGCTAA GGGAACTCGC

**L46**  CAGTAGTAGA TAGGATTTGA GAAACCATGA TTCAGTTGAG AAGTTGTGTA TGATAAACAA CAACAAAAAT ACACAAACTA TCCAGGCTAA GGGAACTCGC

**....|....| ....|....| ....|....| ....|....| ....|....| ....|....| ....|....| ....|....| ....|....| ....|....|**

**4110 4120 4130 4140 4150 4160 4170 4180 4190 4200**

**Morex**  ATT------G CTTAATAGCT AGAATGTAAA TGAG-ACATG GCCGGCCAAA TAATGTTTGG TTGCAGATCT CTGGCATTCA CTGGTGGTAC AAGGTTCCAA

**Harrington**  ATT------G CTTAATAGCT AGAATGTAAA TGAG-ACATG GCCGGCCAAA TAATGTTTGG TTGCAGATCT CTGGCATTCA CTGGTGGTAC AAGGTTCCAA

**HA52**  ATT------G CTTAATAGCT AGAATGTAAA TGAGGACATG GCCGGCCAAA TAATGTTTGG TTGCAGATCT CTGGCATTCA CTGGTGGTAC AAGGTTCCAA

**z043**  ATT------G CTTAATAGCT AGAATGTAAA TGAG-ACATG GCCGGCCAAA TAATGTTTGG TTGCAGATCT CTGGCATTCA CTGGTGGTAC AAGGTTCCAA

**L47**  ATT------G CTTAATAGCT AGAATGTAAA TGAG-ACATG GCCGGCCAAA TAATGTTTGG TTGCAGATCT CTGGCATTCA CTGGTGGTAC AAGGTTCCAA

**Strider**  ATT------G CTTAATAGCT AGAATGTAAA TGAG-ACATG GCCGGCCAAA TAATGTTTGG TTGCAGATCT CTGGCATTCA CTGGTGGTAC AAGGTTCCAA

**W127**  ATT------G CTTAATAGCT AGAATGTAAA TGAG-ACATG GCCGGCCAAA TAATGTTTGG TTGCAGATCT CTGGCATTCA CTGGTGGTAC AAGGTTCCAA

**Adorra**  ATT------G CTTAATAGCT AGAATGTAAA TGAG-ACATG GCCGGCCAAA TAATGCTTGG TTGCAGATCT CTGGCATTCA CTGGTGGTAC AAGGTTCCAA

**Haruna Nijo**  ATT------G CTTAATAGCT AGAATGTAAA TGAG-ACATG GCCGGCCAAA TAATGTTTGG TTGCAGATCT CTGGCATTCA CTGGTGGTAC AAGGTTCCAA

**L35**  ATT------G CTTAATAGCT AGAATGTAAA TGAG-ACATG GCCGGCCAAA TAATGTTTGG TTGTAGATCT CTGGCATTCA CTGGTGGTAC AAGGTTCCAA

**L48**  ATTCGCATTG CTTAATAGCT AGAATGTAAA TGAG-ACATG GCCGGCCAAA TAATGTTTGG TTGCAGATCT CTGGCATTCA CTGGTGGTAC AAGGTTCCAA

**L68**  ATT------G CTTAATAGCT AGAATGTAAA TGAG-ACATG GCCGGCCAAA TAATGTTTGG TTGCAGATCT CTGGCATTCA CTGGTGGTAC AAGGTTCCAA

**PI296897**  ATT------G CTTAATAGCT AGAATGTAAA TGAG-ACATG GCCGGCCAAA TAATGTTTGG TTGCAGATCT CTGGCATTCA CTGGTGGTAC AAGGTTCCAA

**AB75**  ATT------G CTTAATAGCT AGAATGTAAA TGAG-ACATG GCCGGCCAAA TAATGTTTGG TTGCAGATCT CTGGCATTCA CTGGTGGTAC AAGGTTCCAA

**m279**  ATT------G CTTAATAGCT AGAATGTAAA TGAG-ACATG GCCGGCCAAA TAATGTTTGG TTGCAGATCT CTGGCATTCA CTGGTGGTAC AAGGTTCCAA

**Legacy**  ATTCGCATTG CTTAATAGCT AGAATGTAAA TGAG-ACATG GCCGGCCAAA TAATGTTTGG TTGCAGATCT CTGGCATTCA CTGGTGGTAC AAGGTTCCAA

**Orca**  ATTCGCATTG CTTAATAGCT AGAATGTAAA TGAG-ACATG GCCGGCCAAA TAATGTTTGG TTGCAGATCT CTGGCATTCA CTGGTGGTAC AAGGTTCCAA

**Tango**  ATTCGCATTG CTTAATAGCT AGAATGTAAA TGAG-ACATG GCCGGCCAAA TAATGTTTGG TTGCAGATCT CTGGCATTCA CTGGTGGTAC AAGGTTCCAA

**UC958**  ATTCGCATTG CTTAATAGCT AGAATGTAAA TGAG-ACATG GCCGGCCAAA TAATGTTTGG TTGCAGATCT CTGGCATTCA CTGGTGGTAC AAGGTTCCAA

**UC960**  ATTCGCATTG CTTAATAGCT AGAATGTAAA TGAG-ACATG GCCGGCCAAA TAATGTTTGG TTGCAGATCT CTGGCATTCA CTGGTGGTAC AAGGTTCCAA

**Steptoe**  ATTCGCATTG CTTAATAGCT AGAATGTAAA TGAG-ACATG GCCGGCCAAA TAATGTTTGG TTGCAGATCT CTGGCATTCA CTGGTGGTAC AAGGTTCCAA

**Stander**  ATTCGCATTG CTTAATAGCT AGAATGTAAA TGAG-ACATG GCCGGCCAAA TAATGTTTGG TTGCAGATCT CTGGCATTCA CTGGTGGTAC AAGGTTCCAA

**Ashqelon**  ATT------G CTTAATAGCT AGAATGTAAA TGAG-ACATG GCCGGCCAAA TAATGTTTGG TTGCAGATCT CTGGCATTCA CTGGTGGTAC AAGGTTCCAA

**L46**  ATTCGCATTG CTTAATAGCT AGAATGTAAA TGAG-ACATG GCCGGCCAAA TAATGTTTGG TTGCAGATCT CTGGCATTCA CTGGTGGTAC AAGGTTCCAA

**....|....| ....|....| ....|....| ....|....| ....|....| ....|....| ....|....| ....|....| ....|....| ....|....|**

**4210 4220 4230 4240 4250 4260 4270 4280 4290 4300**

**Morex**  GCCATGCAGC CGAGCTCACA GCTGGGTACT ACAACTTACA TGATAGAGAC GGCTACAGAA CCATAGCACG CATGCTCAAA AGGCACCGTG CTAGCATTAA

**Harrington**  GCCATGCAGC CGAGCTCACA GCTGGGTACT ACAACTTACA TGATAGAGAC GGCTACAGAA CCATAGCACG CATGCTCAAA AGGCACCGTG CTAGCATTAA

**HA52**  GCCATGCAGC CGAGCTCACA GCTGGGTACT ACAACTTACA TGATAGAGAC GGCTACAGAA CCATAGCACG CATGCTCAAA AGGCACCGTG CTAGCATTAA

**z043**  GCCATGCAGC CGAGCTCACA GCTGGGTACT ACAACTTACA TGATAGAGAC GGCTACAGAA CCATAGCACG CATGCTCAAA AGGCACCGTG CTAGCATTAA

**L47**  GCCATGCAGC CGAGCTCACA GCTGGGTACT ACAACTTACA TGATAGAGAC GGCTACAGAA CCATAGCACG CATGCTCAAA AGGCACCGTG CTAGCATTAA

**Strider**  GCCATGCAGC CGAGCTCACA GCTGGGTACT ACAACTTACA TGATAGAGAC GGCTACAGAA CCATAGCACG CATGCTCAAA AGGCACCGTG CTAGCATTAA

**W127**  GCCATGCAGC CGAGCTCACA GCTGGGTACT ACAACTTACA TGATAGAGAC GGCTACAGAA CCATAGCACG CATGCTCAAA AGGCACCGTG CTAGCATTAA

**Adorra**  GCCATGCAGC CGAGCTCACA GCTGGGTACT ACAACTTACA TGATAGAGAC GGCTACAGAA CCATAGCACG CATGCTCAAA AGGCACCGTG CTAGCATTAA

**Haruna Nijo**  GCCATGCAGC CGAGCTCACA GCTGGGTACT ATAACTTACA TGATAGAGAC GGCTACAGAA CCATAGCACG CATGCTCAAA AGGCACCGTG CTAGCATTAA

**L35**  GCCATGCAGC CGAGCTCACA GCTGGGTACT ACAACTTACA TGATAGAGAC GGCTACAGAA CCATAGCACG CATGCTCAAA AGGCACCGTG CTAGCATTAA

**L48**  GCCATGCAGC CGAGCTCACA GCTGGGTACT ACAACTTACA TGATAGAGAC GGCTACAGAA CCATAGCACG CATGCTCAAA AGGCACCGTG CTAGCATTAA

**L68**  GCCATGCAGC CGAGCTCACA GCTGGGTACT ACAACTTACA TGATAGAGAC GGCTACAGAA CCATAGCACG CATGCTCAAA AGGCACCGTG CTAGCATTAA

**PI296897**  GCCATGCAGC CGAGCTCACA GCTGGGTACT ACAACTTACA TGATAGAGAC GGCTACAGAA CCATAGCACG CATGCTCAAA AGGCACCGTG CTAGCATTAA

**AB75**  GCCATGCAGC CGAGCTCACA GCTGGGTACT ACAACTTACA TGATAGAGAC GGCTACAGAA CCATAGCACG CATGCTCAAA AGGCACCGTG CTAGCATTAA

**m279**  GCCATGCAGC CGAGCTCACA GCTGGGTACT ACAACTTACA TGATAGAGAC GGCTACAGAA CCATAGCACG CATGCTCAAA AGGCACCGTG CTAGCATTAA

**Legacy**  GCCATGCAGC CGAGCTCACA GCTGGGTACT ACAACTTACA TGATAGAGAC GGCTACAGAA CCATAGCACG CATGCTCAAA AGGCACCGTG CTAGCATTAA

**Orca**  GCCATGCAGC CGAGCTCACA GCTGGGTACT ACAACTTACA TGATAGAGAC GGCTACAGAA CCATAGCACG CATGCTCAAA AGGCACCGTG CTAGCATTAA

**Tango**  GCCATGCAGC CGAGCTCACA GCTGGGTACT ACAACTTACA TGATAGAGAC GGCTACAGAA CCATAGCACG CATGCTCAAA AGGCACCGTG CTAGCATTAA

**UC958**  GCCATGCAGC CGAGCTCACA GCTGGGTACT ACAACTTACA TGATAGAGAC GGCTACAGAA CCATAGCACG CATGCTCAAA AGGCACCGTG CTAGCATTAA

**UC960**  GCCATGCAGC CGAGCTCACA GCTGGGTACT ACAACTTACA TGATAGAGAC GGCTACAGAA CCATAGCACG CATGCTCAAA AGGCACCGTG CTAGCATTAA

**Steptoe**  GCCATGCAGC CGAGCTCACA GCTGGGTACT ACAACTTACA TGATAGAGAC GGCTACAGAA CCATAGCACG CATGCTCAAA AGGCACCGTG CTAGCATTAA

**Stander**  GCCATGCAGC CGAGCTCACA GCTGGGTACT ACAACTTACA TGATAGAGAC GGCTACAGAA CCATAGCACG CATGCTCAAA AGGCACCGTG CTAGCATTAA

**Ashqelon**  GCCATGCAGC CGAGCTCACA GCTGGGTACT ACAACTTACA TGATAGAGAC GGCTACAGAA CCATAGCACG CATGCTCAAA AGGCACCGTG CTAGCATTAA

**L46**  GCCATGCAGC CGAGCTCACA GCTGGGTACT ACAACTTACA TGATAGAGAC GGCTACAGAA CCATAGCACG CATGCTCAAA AGGCACCGTG CTAGCATTAA

**....|....| ....|....| ....|....| ....|....| ....|....| ....|....| ....|....| ....|....| ....|....| ....|....|**

**4310 4320 4330 4340 4350 4360 4370 4380 4390 4400**

**Morex**  CTTCACTTGC GCGGAGATGA GGGATTCGGA GCAAAGCTCG CAGGCGATGA GCGCACCAGA AGAACTAGTC CAACAGGTAG GTAATAACTT ATGCGTTCAG

**Harrington**  CTTCACTTGC GCGGAGATGA GGGATTCGGA GCAAAGCTCG CAGGCGATGA GCGCACCAGA AGAACTAGTC CAACAGGTAG GTAATAACTT ATGCGTTCAG

**HA52**  CTTCACTTGC GCGGAGATGA GGGATTCGGA GCAAAGCTCG CAGGCGATGA GCGCACCAGA AGAACTAGTC CAACAGGTAG GTAATAACTT ATGCGTTCAG

**z043**  CTTCACTTGC GCGGAGATGA GGGATTCGGA GCAAAGCTCG CAGGCGATGA GCGCACCAGA AGAACTAGTC CAACAGGTAG GTAATAACCT ATGCGTTCAG

**L47**  CTTCACTTGC GCGGAGATGA GGGATTCGGA GCAAAGCTCG CAGGCGATGA GCGCACCAGA AGAACTAGTC CAACAGGTAG GTAATAACTT ATGCGTTCAG

**Strider**  CTTCACTTGC GCGGAGATGA GGGATTCGGA GCAAAGCTCG CAGGCGATGA GCGCACCAGA AGAACTAGTC CAACAGGTAG GTAATAACTT ATGCGTTCAG

**W127**  CTTCACTTGC GCGGAGATGA GGGATTCGGA GCAAAGCTCG CAGGCGATGA GCGCACCAGA AGAACTAGTC CAACAGGTAG GTAATAACTT ATGCGTTCAG

**Adorra**  CTTCACTTGC GCGGAGATGA GGGATTTGGA GCAAAGCTCG CAGGCGATGA GCGCACCAGA AGAACTAGTC CAACAGGTAG GTAATAACTT ATGCGTTCAG

**Haruna Nijo**  CTTCACTTGC GCGGAGATGA GGGATTCGGA GCAAAGCTCG CAGGCGATGA GCGCACCAGA AGAACTAGTC CAACAGGTAG GTAATAACTT ATGCGTTCAG

**L35**  CTTCACTTGC GCGGAGATGA GGGATTCGGA GCAAAGCTCG CAGGCGATGA GCGCACCAGA AGAACTAGTC CAACAGGTAG GTAATAACTT ATGCGTTCAG

**L48**  CTTCACTTGC GCGGAGATGA GGGATTCGGA GCAAAGCTCG CAGGCGATGA GCGCACCAGA AGAACTAGTC CAACAGGTAG GTAATAACTT ATGCGTTCAG

**L68**  CTTCACTTGC GCGGAGATGA GGGATTCGGA GCAAAGCTCG CAGGCGATGA GCGCACCAGA AGAACTAGTC CAACAGGTAG GTAATAACTT ATGCGTTCAG

**PI296897**  CTTCACTTGC GCGGAGATGA GGGATTCGGA GCAAAGCTCG CAGGCGATGA GCGCACCAGA AGAACTAGTC CAACAGGTAG GTAATAACTT ATGCGTTCAG

**AB75**  CTTCACTTGC GCGGAGATGA GGGATTCGGA GCAAAGCTCG CAGGCGATGA GCGCACCAGA AGAACTAGTC CAACAGGTAG GTAATAACTT ATGCGTTCAG

**m279**  CTTCACTTGC GCGGAGATGA GGGATTTGGA GCAAAGCTCG CAGGCGATGA GCGCACCAGA AGAACTAGTC CAACAGGTAG GTAATAACTT ATGCGTTCAG

**Legacy**  CTTCACTTGC GCGGAGATGA GGGATTCGGA GCAAAGCTCG CAGGCGATGA GCGCACCAGA AGAACTAGTC CAACAGGTAG GTAATAACTT ATGCGTTCAG

**Orca**  CTTCACTTGC GCGGAGATGA GGGATTCGGA GCAAAGCTCG CAGGCGATGA GCGCACCAGA AGAACTAGTC CAACAGGTAG GTAATAACTT ATGCGTTCAG

**Tango**  CTTCACTTGC GCGGAGATGA GGGATTCGGA GCAAAGCTCG CAGGCGATGA GCGCACCAGA AGAACTAGTC CAACAGGTAG GTAATAACTT ATGCGTTCAG

**UC958**  CTTCACTTGC GCGGAGATGA GGGATTCGGA GCAAAGCTCG CAGGCGATGA GCGCACCAGA AGAACTAGTC CAACAGGTAG GTAATAACTT ATGCGTTCAG

**UC960**  CTTCACTTGC GCGGAGATGA GGGATTCGGA GCAAAGCTCG CAGGCGATGA GCGCACCAGA AGAACTAGTC CAACAGGTAG GTAATAACTT ATGCGTTCAG

**Steptoe**  CTTCACTTGC GCGGAGATGA GGGATTCGGA GCAAAGCTCG CAGGCGATGA GCGCACCAGA AGAACTAGTC CAACAGGTAG GTAATAACTT ATGCGTTCAG

**Stander**  CTTCACTTGC GCGGAGATGA GGGATTCGGA GCAAAGCTCG CAGGCGATGA GCGCACCAGA AGAACTAGTC CAACAGGTAG GTAATAACTT ATGCGTTCAG

**Ashqelon**  CTTCACTTGC GCGGAGATGA GGGATTCGGA GCAAAGCTCG CAGGCGATGA GCGCACCAGA AGAACTAGTC CAACAGGTAG GTAATAACTT ATGCGTTCAG

**L46**  CTTCACTTGC GCGGAGATGA GGGATTCGGA GCAAAGCTCG CAGGCGATGA GCGCACCAGA AGAACTAGTC CAACAGGTAG GTAATAACTT ATGCGTTCAG

**....|....| ....|....| ....|....| ....|....| ....|....| ....|....| ....|....| ....|....| ....|....| ....|....|**

**4410 4420 4430 4440 4450 4460 4470 4480 4490 4500**

**Morex**  ATATATTACG CTTATATATC TACGTA-TAT ACTATGATGG AAACACCTTT TTTTTTT-AG AAAAGGAGGC TTAGCTCCGG CCTCTGCATC GAAAGATGCA

**Harrington**  ATATATTACG CTTATATATC TACGTA-TAT ACTATGATGG AAACACCTTT TTTTTTT-AG AAAAGGAGGC TTAGCTCCGG CCTCTGCATC GAAAGATGCA

**HA52**  ATATATTACG CTTATATATC TACGTAATAT ACTATGATGG AAACACCTTT TTTTTTT-AG AAAAGGAGGC TTAGCTCCGG CCTCTGCATC GAAAGATGCA

**z043**  ATATATTACG CTTATATATC TACGTA-TAT ACTATGATGG AAACACCTTT TTTTTTTTAG AAAAGGAGGC TTAGCTCCGG CCTCTGCATC GAAAGATGCA

**L47**  ATATATTACG CTTATATATC TACGTA-TAT ACTATGATGG AAACACCTTT TTTTTTT-AG AAAAGGAGGC TTAGCTCCGG CCTCTGCATC GAAAGATGCA

**Strider**  ATATATTACG CTTATATATC TACGTA-TAT ACTATGATGG AAACACCTTT TTTTTT--AG AAAAGGAGGC TTAGCCCTGG CCTCTGCATC GAAAGATGCA

**W127**  ATATATTACG CTTATATATC TACGTA-TAT ACTATGATGG AAACACCTTT TTTTTTTTAG AAAAGGAGGC TTAGCTCCGG CCTCTGCATC GAAAGATGCA

**Adorra**  ATATATTACG CTTATATATC TACGTA-TAT ACTATGATGG AAACACCTTT TTTTTT--AG AAAAGGAGGC TTAGCCCCGG CCTCTGCATC GAAAGATGCA

**Haruna Nijo**  ATATATTACG CTTATATATC TACGTA-TAT ACTATGATGG AAACACCTTT TTCTTT--AG AAAAGGAGGC TTAGCCCCGG CCTCTGCATC GAAAGATGCA

**L35**  ATATATTACG CTTATATATC TACGTA-TAT ACTATGATGG AAACACCTTT TTTTTT--AG AAAAGGAGGC TTAGCCCCGG CCTCTGCATC GAAAGATGCA

**L48**  ATATATTACG CTTATATATC TACGTA-TAT ACTATGATGG AAACACCTTT TTTTTTT-AG AAAAGGAGGC TTAGCTCCGG CCTCTGCATC GAAAGATGCA

**L68**  ATATATTACG CTTATATATC TACGTA-TAT ACTATGATGG AAACACCTTT TTTTTT--AG AAAAGGAGGC TTAGCCCCGG CCTCTGCATC GAAAGATGCA

**PI296897**  ATATATTACG CTTATATATC TACGTA-TAT ACTATGATGG AAACACCTTT TTCTTT--AG AAAAGGAGGC TTAGCCCCGG CCTCTGCATC GAAAGATGCA

**AB75**  ATATATTACG CTTATATATC TACGTA-TAT ACTATGATGG GAACACCTTT TTTTTT--AG AAAAGGAGGC TTAGCCCCGG CCTCTGCATC GAAAG-----

**m279**  ATATATTACG CTTATATATC TACGTA-TAT ACTATGATGG AAACACCTTT TTTTTT--AG AAAAGGAGGC TTAGCCCCGG CCTCTGCATC GAAAGATGCA

**Legacy**  ATATATTACG CTTATATATC TACGTA-TAT ACTATGATGG AAACACCTTT TTTTTT--AG AAAAGGAGGC TTAGCCCCGG CCTCTGCATC GAAAGATGCA

**Orca**  ATATATTACG CTTATATATC TACGTA-TAT ACTATGATGG AAACACCTTT TTTTTTT-AG AAAAGGAGGC TTAGCTCCGG CCTCTGCATC GAAAGATGCA

**Tango**  ATATATTACG CTTATATATC TACGTA-TAT ACTATGATGG AAACACCTTT TTTTTTT-AG AAAAGGAGGC TTAGCTCCGG CCTCTGCATC GAAAGATGCA

**UC958**  ATATATTACG CTTATATATC TACGTA-TAT ACTATGATGG AAACACCTTT TTTTTTT-AG AAAAGGAGGC TTAGCTCCGG CCTCTGCATC GAAAGATGCA

**UC960**  ATATATTACG CTTATATATC TACGTA-TAT ACTATGATGG AAACACCTTT TTTTTTT-AG AAAAGGAGGC TTAGCTCCGG CCTCTGCATC GAAAGATGCA

**Steptoe**  ATATATTACG CTTATATATC TACGTA-TAT ACTATGATGG AAACACCTTT TTTTTTT-AG AAAAGGAGGC TTAGCTCCGG CCTCTGCATC GAAAGATGCA

**Stander**  ATATATTACG CTTATATATC TACGTA-TAT ACTATGATGG AAACACCTTT TTTTTTT-AG AAAAGGAGGC TTAGCTCCGG CCTCTGCATC GAAAGATGCA

**Ashqelon**  ATATATTACG CTTATATATC TACGTA-TAT ACTATGATGG AAACACCTTT TTTTTT--AG AAAAGGAGGC TTAGCCCCGG CCTCTGCATC GAAAGATGCA

**L46**  ATATATTACG CTTATATATC TACGTA-TAT ACTATGATGG AAACACCTTT TTTTTTT-AG AAAAGGAGGC TTAGCTCCGG CCTCTGCATC GAAAGATGCA

**....|....| ....|....| ....|....| ....|....| ....|....| ....|....| ....|....| ....|....| ....|....| ....|....|**

**4510 4520 4530 4540 4550 4560 4570 4580 4590 4600**

**Morex**  T-ACGGGCCA TGATGATGAA AACACCTAAA TCACTTGTCG TCAAAATAAT TTCTCAGGTG TTGAGTGCTG GATGGAGAGA GGGCCTAAAT GTGGCATGCG

**Harrington**  T-ACGGGCCA TGATGATGAA AACACCTAAA TCACTTGTCG TCAAAATAAT TTCTCAGGTG TTGAGTGCTG GATGGAGAGA GGGCCTAAAT GTGGCATGCG

**HA52**  TTACGGGCCA TGATGATGAA AACACCTAAA TCACTTGTCG TCAAAATAAT TTCTCAGGTG TTGAGTGCTG GATGGAGAGA GGGCCTAAAT GTGGCATGCG

**z043**  T-ACGGGCCA TGATGATGGA AACACCTAAA TCACTTGTCG TCAAAATAAT TTCTCAGGTG TTGAGTGCTG GATGGAGAGA GGGCCTAAAT GTGGCATGCG

**L47**  TTACGGGCCA TGATGATGAA AACACCTAAA TCACTTGTCG TCAAAATAAT TTCTCAGGTG TTGAGTGCTG GATGGAGAGA GGGCCTAAAT GTGGCATGCG

**Strider**  T-ACGG-CCA TGATGATGGA AACACCTAAA TCACTTGTCG TCAAAATAAT TTCTCAGGTG TTGAGTGCTG GATGGAGAGA GGGCCTAAAT GTGGCATGCG

**W127**  T-ACGGGCCA TGATGATGGA AACACCTAAA TCACTTGTCG TCAAAATAAT TTCTCAGGTG TTGAGTGCTG GATGGAGAGA GGGCCTAAAT GTGGCATGCG

**Adorra**  T-ACGG-CCA TGATGATGGA AACACCTAAA TCACTTGTCG TCAAAATAAT TTCTCAGGTG TTGAGTGCTG GATGGAGAGA GGGCCTAAAT GTGGCATGCG

**Haruna Nijo**  T-ACGG-CCA TGATGATGGA AACACCTAAA TCACTTGTCG TCAAAATAAT TTCTCAGGTG TTGAGTGCTG GATGGAGAGA GGGCCTAAAT GTGGCATGCG

**L35**  T-ACGG-CCA TGATGATGGA AACACCTAAA TCACTTGTCG TCAAAATAAT TTCTCAGGTG TTGAGTGCTG GATGGAGAGA GGGCCTAAAT GTGGCATGCG

**L48**  T-ACGG-CCA TGATGATGGA AACACCTAAA TCACTTGTCG TCAAAATAAT TTCTCAGGTG TTGAGTGCTG GATGGAGAGA GGGCCTAAAT GTGGCATGCG

**L68**  T-ACGG-CCA TGATGATGGA AACACCTAAA TCACTTGTCG TCAAAATAAT TTCTCAGGTG TTGAGTGCTG GATGGAGAGA GGGCCTAAAT GTGGCATGCG

**PI296897**  T-ACGG-CCA TGATGATGGA AACACCTAAA TCACTTGTCG TCAAAATAAT TTCTCAGGTG TTGAGTGCTG GATGGAGAGA GGGCCTAAAT GTGGCATGCG

**AB75**  -------CCA TGATGATGGA AACACCTAAA TCACTTGTCG TCAAAATAAT TTCTCAGGTG TTGAGTGCTG GATGGAGAGA GGGCCTAAAT GTGGCATGCG

**m279**  T-ACGG-CCA TGATGATGGA AACACCTAAA TCACTTGTCG TCAAAATAAT TTCTCAGGTG TTGAGTGCTG GATGGAGAGA GGGCCTAAAT GTGGCATGCG

**Legacy**  T-ACGG-CCA TGATGATGGA AACACCTAAA TCACTTGTCG TCAAAATAAT TTCTCAGGTG TTGAGTGCTG GATGGAGAGA GGGCCTAAAT GTGGCATGCG

**Orca**  T-ACGG-CCA TGATGATGGA AACACCTAAA TCACTTGTCG TCAAAATAAT TTCTCAGGTG TTGAGTGCTG GATGGAGAGA GGGCCTAAAT GTGGCATGCG

**Tango**  T-ACGG-CCA TGATGATGGA AACACCTAAA TCACTTGTCG TCAAAATAAT TTCTCAGGTG TTGAGTGCTG GATGGAGAGA GGGCCTAAAT GTGGCATGCG

**UC958**  T-ACGG-CCA TGATGATGGA AACACCTAAA TCACTTGTCG TCAAAATAAT TTCTCAGGTG TTGAGTGCTG GATGGAGAGA GGGCCTAAAT GTGGCATGCG

**UC960**  T-ACGG-CCA TGATGATGGA AACACCTAAA TCACTTGTCG TCAAAATAAT TTCTCAGGTG TTGAGTGCTG GATGGAGAGA GGGCCTAAAT GTGGCATGCG

**Steptoe**  T-ACGG-CCA TGATGATGGA AACACCTAAA TCACTTGTCG TCAAAATAAT TTCTCAGGTG TTGAGTGCTG GATGGAGAGA GGGCCTAAAT GTGGCATGCG

**Stander**  T-ACGG-CCA TGATGATGGA AACACCTAAA TCACTTGTCG TCAAAATAAT TTCTCAGGTG TTGAGTGCTG GATGGAGAGA GGGCCTAAAT GTGGCATGCG

**Ashqelon**  T-ACGG-CCA TGATGATGGA AACACCTAAA TCACTTGTCG TCAAAATAAT TTCTCAGGTG TTGAGTGCTG GATGGAGAGA GGGCCTAAAT GTGGCATGCG

**L46**  T-ACGG-CCA TGATGATGGA AACACCTAAA TCACTTGTCG TCAAAATAAT TTCTCAGGTG TTGAGTGCTG GATGGAGAGA GGGCCTAAAT GTGGCATGCG

**....|....| ....|....| ....|....| ....|....| ....|....| ....|....| ....|....| ....|....| ....|....| ....|....|**

**4610 4620 4630 4640 4650 4660 4670 4680 4690 4700**

**Morex**  AAAACGCGCT TCCACGATAT GATCCAACTG CTTACAACAC CATACTCAGG AATGCGAGGC CTCATGGAAT CAACCAGAGC GGCCCTCCTG AGCACAAGCT

**Harrington**  AAAACGCGCT TCCACGATAT GATCCAACTG CTTACAACAC CATACTCAGG AATGCGAGGC CTCATGGAAT CAACCAGAGC GGCCCTCCTG AGCACAAGCT

**HA52**  AAAACGCGCT TCCACGATAT GATCCAACTG CTTACAACAC CATACTCAGG AATGCGAGGC CTCATGGAAT CAACCAGAGC GGCCCTCCTG AGCACAAGCT

**z043**  AAAACGCGCT TCCACGATAT GATCCAACTG CTTACAACAC CATACTCAGG AATGCGAGGC CTCATGGAAT CAACCAGAGC GGCCCTCCTG AGCACAAGCT

**L47**  AAAACGCGCT TCCACGATAT GATCCAACTG CTTACAACAC CATACTCAGG AATGCGAGGC CTCATGGAAT CAACCAGAGC GGCCCTCCTG AGCACAGGCT

**Strider**  AAAACGCGCT TCCACGATAT GATCCAACTG CTTACAACAC CATACTCAGG AATGCGAGGC CTCATGGAAT CAACCAGAGC GGCCCTCCTG AGCACAAGCT

**W127**  AAAACGCGCT TCCACGATAT GATCCAGCTG CTTACAACAC CATACTCAGG AATGCGAGGC CTCATGGAAT CAACCAGAGC GGCCCTCCTG AGCACAAGCT

**Adorra**  AAAACGCGCT TCCACGATAT GATCCAACTG CTTACAACAC CATACTCAGG AATGCGAGGC CTCATGGAAT CAACCAGAGC GGCCCTCCTG AGCACAAGCT

**Haruna Nijo**  AAAACGCGCT TCCACGATAT GATCCAACTG CTTACAACAC CATACTCAGG AATGCGAGGC CTCATGGAAT CAACCAGAGC GGCCCTCCTG AGCACAAGCT

**L35**  AAAACGCACT TCCACGATAT GATCCAACTG CTTACAACAC CATACTCAGG AATGCGAGGC CTCATGGAAT CAACCAGAGC GGCCCTCCTG AGCACAAGCT

**L48**  AAAACGCGCT TCCACGATAT GATCCAACTG CTTACAACAC CATACTCAGG AATGCGAGGC CTCATGGAAT CAACCAGAGC GGCCCTCCTG AGCACAAGCT

**L68**  AAAACGCGCT TCCACGATAT GATCCAACTG CTTACAACAC CATACTCAGG AATGCGAGGC CTCATGGAAT CAACCAGAGC GGCCCTCCTG AGCACAAGCT

**PI296897**  AAAACGCGCT TCCACGATAT GATCCAACTG CTTACAACAC CATACTCAGG AATGCGAGGC CTCATGGAAT CAACCAGAGC GGCCCTCCTG AGCACAAGCT

**AB75**  AAAACGCGCT TCCACGATAT GATCCAACTG CTTACAACAC CATACTCAGG AATGCGAGGC CTCATGGAAT CAACCAGAGC GGCCCTCCTG AGCACAAGCT

**m279**  AAAACGCGCT TCCACGATAT GATCCAACTG CTTACAACAC CATACTCAGG AATGCGAGGC CTCATGGAAT CAACCAGAGC GGCCCTCCTG AGCACAAGCT

**Legacy**  AAAACGCGCT TCCACGATAT GATCCAACTG CTTACAACAC CATACTCAGG AATGCGAGGC CTCATGGAAT CAACCAGAGC GGCCCTCCTG AGCACAAGCT

**Orca**  AAAACGCGCT TCCACGATAT GATCCAACTG CTTACAACAC CATACTCAGG AATGCGAGGC CTCATGGAAT CAACCAGAGC GGCCCTCCTG AGCACAAGCT

**Tango**  AAAACGCGCT TCCACGATAT GATCCAACTG CTTACAACAC CATACTCAGG AATGCGAGGC CTCATGGAAT CAACCAGAGC GGCCCTCCTG AGCACAAGCT

**UC958**  AAAACGCGCT TCCACGATAT GATCCAACTG CTTACAACAC CATACTCAGG AATGCGAGGC CTCATGGAAT CAACCAGAGC GGCCCTCCTG AGCACAAGCT

**UC960**  AAAACGCGCT TCCACGATAT GATCCAACTG CTTACAACAC CATACTCAGG AATGCGAGGC CTCATGGAAT CAACCAGAGC GGCCCTCCTG AGCACAAGCT

**Steptoe**  AAAACGCGCT TCCACGATAT GATCCAACTG CTTACAACAC CATACTCAGG AATGCGAGGC CTCATGGAAT CAACCAGAGC GGCCCTCCTG AGCACAAGCT

**Stander**  AAAACGCGCT TCCACGATAT GATCCAACTG CTTACAACAC CATACTCAGG AATGCGAGGC CTCATGGAAT CAACCAGAGC GGCCCTCCTG AGCACAAGCT

**Ashqelon**  AAAACGCGCT TCCACGATAT GATCCAACTG CTTACAACAC CATACTCAGG AATGCGAGGC CTCATGGAAT CAACCAGAGC GGCCCTCCTG AGCACAAGCT

**L46**  AAAACGCGCT TCCACGATAT GATCCAACTG CTTACAACAC CATACTCAGG AATGCGAGGC CTCATGGAAT CAACCAGAGC GGCCCTCCTG AGCACAAGCT

**....|....| ....|....| ....|....| ....|....| ....|....| ....|....| ....|....| ....|....| ....|....| ....|....|**

**4710 4720 4730 4740 4750 4760 4770 4780 4790 4800**

**Morex**  GTTTGGATTC ACCTACCTTC GGCTGTCGAA TCAGCTGGTG GAGGGACAAA ACTATGCCAA TTTCAAGACC TTTGTCGACA GAATGCATGC CAACCTGGTT

**Harrington**  GTTTGGATTC ACCTACCTTC GGCTGTCGAA TCAGCTGGTG GAGGGACAAA ACTATGCCAA TTTCAAGACC TTTGTCGACA GAATGCATGC CAACCTGGTT

**HA52**  GTTTGGATTC ACCTACCTTC GGCTGTCGAA TCAGCTGGTG GAGGGACAAA ACTATGCCAA TTTCAAGACC TTTGTCGACA GAATGCATGC CAACCTGGTT

**z043**  GTTTGGATTC ACCTACCTTC GGCTGTCGAA TCAGCTGGTG GAGGGACAAA ACTATGCCAA TTTCAAGACC TTTGTCGACA GAATGCATGC CAACCTGGTT

**L47**  GTTTGGATTC ACCTACCTTC GGCTGTCGAA TCAGCTGGTG GAGGGACAAA ACTATGCCAA TTTCAAGACC TTTGTCGACA GAATGCATGC CAACCTGGTT

**Strider**  GTTTGGATTC ACCTACCTTC GGCTGTCGAA TCAGCTGGTG GAGGGACAAA ACTATGTCAA TTTCAAGACC TTTGTCGACA GAATGCATGC CAACCTGGTT

**W127**  GTTTGGATTC ACCTACCTTC GGCTGTCGAA TCAGCTGGTG GAGGGACAAA ACTATGTCAA TTTCAAGACC TTTGTCGACA GAATGCATGC CAACCTGGTT

**Adorra**  GTTTGGATTC ACCTACCTTC GGCTGTCGAA TCAGCTGGTG GAGGGACAAA ACTATGTCAA TTTCAAGACC TTTGTCGACA GAATGCATGC CAACCTGGTT

**Haruna Nijo**  GTTTGGATTC ACCTACCTTC GGCTGTCGAA TCAGCTGGTG GAGGGACAAA ACTATGTCAA CTTCAAGACC TTTGTCGACA GAATGCATGC CAACCTGGTT

**L35**  GTTTGGATTC ACCTACCTTC GGCTGTCGAA TCAGCTGGTG GAGGGACAAA ACTATGTCAA TTTCAAGACC TTTGTCGACA GAATGCATGC CAACCTGGTT

**L48**  GTTTGGATTC ACCTACCTTC GGCTGTCGAA TCAGCTGGTG GAGGGACAAA ACTATGTCAA TTTCAAGACC TTTGTCGACA GAATGCATGC CAACCTGGTT

**L68**  GTTTGGATTC ACCTACCTTC GGCTGTCGAA TCAGCTGGTG GAGGGACAAA ACTATGTCAA TTTCAAGACC TTTGTCGACA GAATGCATGC CAACCTGGTT

**PI296897**  GTTTGGATTC ACCTACCTTC GGCTGTCGAA TCAGCTGGTG GAGGGACAAA ACTATGTCAA TTTCAAGACC TTTGTCGACA GAATGCATGC CAACCTGGTT

**AB75**  GTTTGGATTC ACCTACCTTC GGCTGTCGAA TCAGCTGGTG GAGGGACAAA ACTATGTCAA TTTCAAGACC TTTGTCGACA GAATGCATGC CAACCTGGTT

**m279**  GTTTGGATTC ACCTACCTTC GGCTGTCGAA TCAGCTGGTG GAGGGACAAA ACTATGTCAA TTTCAAGACC TTTGTCGACA GAATGCATGC CAACCTGGTT

**Legacy**  GTTTGGATTC ACCTACCTTC GGCTGTCGAA TCAGCTGGTG GAGGGACAAA ACTATGTCAA TTTCAAGACC TTTGTCGACA GAATGCATGC CAACCTGGTT

**Orca**  GTTTGGATTC ACCTACCTTC GGCTGTCGAA TCAGCTGGTG GAGGGACAAA ACTATGTCAA TTTCAAGACC TTTGTCGACA GAATGCATGC CAACCTGGTT

**Tango**  GTTTGGATTC ACCTACCTTC GGCTGTCGAA TCAGCTGGTG GAGGGACAAA ACTATGTCAA TTTCAAGACC TTTGTCGACA GAATGCATGC CAACCTGGTT

**UC958**  GTTTGGATTC ACCTACCTTC GGCTGTCGAA TCAGCTGGTG GAGGGACAAA ACTATGTCAA TTTCAAGACC TTTGTCGACA GAATGCATGC CAACCTGGTT

**UC960**  GTTTGGATTC ACCTACCTTC GGCTGTCGAA TCAGCTGGTG GAGGGACAAA ACTATGTCAA TTTCAAGACC TTTGTCGACA GAATGCATGC CAACCTGGTT

**Steptoe**  GTTTGGATTC ACCTACCTTC GGCTGTCGAA TCAGCTGGTG GAGGGACAAA ACTATGTCAA TTTCAAGACC TTTGTCGACA GAATGCATGC CAACCTGGTT

**Stander**  GTTTGGATTC ACCTACCTTC GGCTGTCGAA TCAGCTGGTG GAGGGACAAA ACTATGTCAA TTTCAAGACC TTTGTCGACA GAATGCATGC CAACCTGGTT

**Ashqelon**  GTTTGGATTC ACCTACCTTC GGCTGTCGAA TCAGCTGGTG GAGGGACAAA ACTATGTCAA TTTCAAGACC TTTGTCGACA GAATGCATGC CAACCTGGTT

**L46**  GTTTGGATTC ACCTACCTTC GGCTGTCGAA TCAGCTGGTG GAGGGACAAA ACTATGTCAA TTTCAAGACC TTTGTCGACA GAATGCATGC CAACCTGGTT

**....|....| ....|....| ....|....| ....|....| ....|....| ....|....| ....|....| ....|....| ....|....| ....|....|**

**4810 4820 4830 4840 4850 4860 4870 4880 4890 4900**

**Morex**  AGTGCCACAA CCACTTACTA ACGCATGTCA AAAATTAAAC ATATACAAGA ACCATTTGTT GATTTGCAGG TGCCTATTAT ATACTAATAA TTTAATTTTA

**Harrington**  AGTGCCACAA CCACTTACTA ACGCATGTCA AAAATTAAAC ATATACAAGA ACCATTTGTT GATTTGCAGG TGCCTATTAT ATACTAATAA TTTAATTTTA

**HA52**  AGTGCCACAA CCACTTACTA ACGCATGTCA AAAATTAAAC ATATACAAGA ACCATTTGTT GATTTGCAGG TGCCTATTAT ATACTAATAA TTTAATTTTA

**z043**  AGTGCCACAA CCACTTACTA ACGCATGTCA AAAATTAAAC ATATACAAGA ACCATTTGTT GATTTGCAGG TGCCTATTAT ATACTAATAA TTTAATTTTA

**L47**  AGTGCCACAA CCACTTACTA ACGCATGTCA AAAATTAAAC ATATACAAGA ACCATTTGTT GATTTGCAGG TGCCTATTAT ATACTAATAA TTTAATTTTA

**Strider**  AGTGCCACAA CCACTTACTA ACGCATGTCA AAAATTAAAC ATATACAAGA ACCATTTGTT GATTTGCAGG TGCCTATTAT ATACTAATAA TTTAATTTTA

**W127**  AGTGCCACAA CCACTTACTA ACGCATGTCA AAAATTAAAC ATATACAAGA ACCATTTGTT GATTTGCAGG TGCCCATTAT ATACTAATAA TTTAATTTTA

**Adorra**  AGTGCCACAA CCACTTACTA ACGCATGTCA AAAATTAAAC ATATACAAGA ACCATTTGTT GATTTGCAGG TGCCTATTAT ATACTAATAA TTTAATTTTA

**Haruna Nijo**  AGTGCCACAA CCACTTACTA ACGCATGTCA AAAATTAAAC ATATACAAGA ACCATTTGTT GATTTGCAGG TGCCTATTAT ATACTAATAA TTTAATTTTA

**L35**  AGTGCCACAA CCACTTACTA ACGCATGTCA AAAATTAAAC ATATACAAGA ACCATTTGTT GATTTGCAGG TGCCTATTAT ATACTAATAA TTTAATTTTA

**L48**  AGTGCCACAA CCACTTACTA ACGCATGTCA AAAATTAAAC ATATACAAGA ACCATTTGTT GATTTGCAGG TGCCTATTAT ATACTAATAA TTTAATTTTA

**L68**  AGTGCCACAA CCACTTACTA ACGCATGTCA AAAATTAAAC ATATACAAGA ACCATTTGTT GATTTGCAGG TGCCTATTAT ATACTAATAA TTTAATTTTA

**PI296897**  AGTGCCACAA CCACTTACTA ACGCATGTCA AAAATTAAAC ATATACAAGA ACCATTTGTT GATTTGCAGG TGCCTATTAT ATACTAATAA TTTAATTTTA

**AB75**  AGTGCCACAA CCACTTACTA ACGCATGTCA AAAATTAAAC ATATACAAGA ACCATTTGTT GATTTGCAGG TGCCCATTAT ATACTAATAA TTTAATTTTA

**m279**  AGTGCCACAA CCACTTACTA ACGCATGTCA AAAATTAAAC ATATACAAGA ACCATTTGTT GATTTGCAGG TGCCTATTAT ATACTAATAA TTTAATTTTA

**Legacy**  AGTGCCACAA CCACTTACTA ACGCATGTCA AAAATTAAAC ATATACAAGA ACCATTTGTT GATTTGCAGG TGCCTATTAT ATACTAATAA TTTAATTTTA

**Orca**  AGTGCCACAA CCACTTACTA ACGCATGTCA AAAATTAAAC ATATACAAGA ACCATTTGTT GATTTGCAGG TGCCTATTAT ATACTAATAA TTTAATTTTA

**Tango**  AGTGCCACAA CCACTTACTA ACGCATGTCA AAAATTAAAC ATATACAAGA ACCATTTGTT GATTTGCAGG TGCCTATTAT ATACTAATAA TTTAATTTTA

**UC958**  AGTGCCACAA CCACTTACTA ACGCATGTCA AAAATTAAAC ATATACAAGA ACCATTTGTT GATTTGCAGG TGCCTATTAT ATACTAATAA TTTAATTTTA

**UC960**  AGTGCCACAA CCACTTACTA ACGCATGTCA AAAATTAAAC ATATACAAGA ACCATTTGTT GATTTGCAGG TGCCTATTAT ATACTAATAA TTTAATTTTA

**Steptoe**  AGTGCCACAA CCACTTACTA ACGCATGTCA AAAATTAAAC ATATACAAGA ACCATTTGTT GATTTGCAGG TGCCTATTAT ATACTAATAA TTTAATTTTA

**Stander**  AGTGCCACAA CCACTTACTA ACGCATGTCA AAAATTAAAC ATATACAAGA ACCATTTGTT GATTTGCAGG TGCCTATTAT ATACTAATAA TTTAATTTTA

**Ashqelon**  AGTGCCACAA CCACTTACTA ACGCATGTCA AAAATTAAAC ATATACAAGA ACCATTTGTT GATTTGCAGG TGCCCATTAT ATACTAATAA TTTAATTTTA

**L46**  AGTGCCACAA CCACTTACTA ACGCATGTCA AAAATTAAAC ATATACAAGA ACCATTTGTT GATTTGCAGG TGCCTATTAT ATACTAATAA TTTAATTTTA

**....|....| ....|....| ....|....| ....|....| ....|....| ....|....| ....|....| ....|....| ....|....| ....|....|**

**4910 4920 4930 4940 4950 4960 4970 4980 4990 5000**

**Morex**  TTGTTTTCAG CCTCGTGACC CATATGTTGA TCCAATGGCG CCTTTGCCAA GATCAGGGCC AGAAATATCG ATTGAGATGA TCCTACAAGC AGCACAGCCA

**Harrington**  TTGTTTTCAG CCTCGTGACC CATATGTTGA TCCAATGGCG CCTTTGCCAA GATCAGGGCC AGAAATATCG ATTGAGATGA TCCTACAAGC AGCACAGCCA

**HA52**  TTGTTTTCAG CCTCGTGACC CATATGTTGA TCCAATGGCG CCTTTGCCAA GATCAGGGCC AGAAATATCG ATTGAGATGA TCCTACAAGC AGCACAGCCA

**z043**  TTGTTTTCAG CCTCGTGACC CATATGTTGA TCCAATGGCG CCTTTGCCAA GATCAGGGCC AGAAATATCG ATTGAGATGA TCCTACAAGC AGCACAGCCA

**L47**  TTGTTTTCAG CCTCGTGACC CATATGTTGA TCCAATGGCG CCTTTGCCAA GATCAGGGCC AGAAATATCG ATTGAGATGA TCCTACAAGC AGCACAGCCA

**Strider**  TTGTTTTCAG CCTCGTGACC CATATGTTGA TCCAATGACG CCTTTGCCAA GATCAGGGCC AGAAATATCG ATTGAGATGA TCCTACAAGC AGCACAGCCA

**W127**  TTGTTTTCAG CCTCGTGACC CATATGTTGA TCCAATGGCG CCTTTGCCAA GATCAGGGCC AGAAATATCG ATTGAGATGA TCCTACAAGC AGCACAGCCA

**Adorra**  TTGTTTTCAG CCTCGTGACC CATATGTTGA TCCAATGGCG CCTTTGCCAA GATCAGGGCC AGAAATATCG ATTGAGATGA TCCTACAAGC AGCACAGCCA

**Haruna Nijo**  TTGTTTTCAG CCTCGTGACC CATATGTTGA TCCAATGGCG CCTTTGCCAA GATCAGGGCC AGAAATATCG ATTGAGATGA TCCTACAAGC AGCACAGCCA

**L35**  TTGTTTTCAG CCTCGTGACC CATATGTTGA TCCAATGGCG CCTTTGCCAA GATCAGGGCC AGAAATATCG ATTGAGATGA TCCTACAAGC AGCACAGCCA

**L48**  TTGTTTCCAG CCTCGTGACC CATATGTTGA TCCAATGACG CCTTTGCCAA GATCAGGGCC AGAAATATCG ATTGAGATGA TCCTACAAGC AGCACAGCCA

**L68**  TTGTTTTCAG CCTCGTGACC CATATGTTGA TCCAATGGCG CCTTTGCCAA GATCAGGGCC AGAAATATCG ATTGAGATGA TCCTACAAGC AGCACAGCCA

**PI296897**  TTGTTTTCAG CCTCGTGACC CATATGTTGA TCCAATGGCG CCTTTGCCAA GATCAGGGCC AGAAATATCG ATTGAGATGA TCCTACAAGC AGCACAGCCA

**AB75**  TTGTTTTCAG CCTCGTGACC CATATGTTGA TCCAATGGCG CCTTTGCCAA GATCAGGGCC AGAAATATCG ATTGAGATGA TCCTACAAGC AGCAAAGCCA

**m279**  TTGTTTTCAG CCTCGTGACC CATATGTTGA TCCAATGGCG CCTTTGCCAA GATCAGGGCC AGAAATATCG ATTGAGATGA TCCTACAAGC AGCACAGCCA

**Legacy**  TTGTTTTCAG CCTCGTGACC CATATGTTGA TCCAATGGCG CCTTTGCCAA GATCAGGGCC AGAAATATCG ATTGAGATGA TCCTACAAGC AGCACAGCCA

**Orca**  TTGTTTTCAG CCTCGTGACC CATATGTTGA TCCAATGGCG CCTTTGCCAA GATCAGGGCC AGAAATATCG ATTGAGATGA TCCTACAAGC AGCACAGCCA

**Tango**  TTGTTTTCAG CCTCGTGACC CATATGTTGA TCCAATGGCG CCTTTGCCAA GATCAGGGCC AGAAATATCG ATTGAGATGA TCCTACAAGC AGCACAGCCA

**UC958**  TTGTTTTCAG CCTCGTGACC CATATGTTGA TCCAATGGCG CCTTTGCCAA GATCAGGGCC AGAAATATCG ATTGAGATGA TCCTACAAGC AGCACAGCCA

**UC960**  TTGTTTTCAG CCTCGTGACC CATATGTTGA TCCAATGGCG CCTTTGCCAA GATCAGGGCC AGAAATATCG ATTGAGATGA TCCTACAAGC AGCACAGCCA

**Steptoe**  TTGTTTTCAG CCTCGTGACC CATATGTTGA TCCAATGGCG CCTTTGCCAA GATCAGGGCC AGAAATATCG ATTGAGATGA TCCTACAAGC AGCACAGCCA

**Stander**  TTGTTTTCAG CCTCGTGACC CATATGTTGA TCCAATGGCG CCTTTGCCAA GATCAGGGCC AGAAATATCG ATTGAGATGA TCCTACAAGC AGCACAGCCA

**Ashqelon**  TTGTTTTCAG CCTCGTGACC CATATGTTGA TCCAATGGCG CCTTTGCCAA GATCAGGGCC AGAAATATCG ATTGAGATGA TCCTACAAGC AGCACAGCCA

**L46**  TTGTTTCCAG CCTCGTGACC CATATGTTGA TCCAATGACG CCTTTGCCAA GATCAGGGCC AGAAATATCG ATTGAGATGA TCCTACAAGC AGCACAGCCA

**....|....| ....|....| ....|....| ....|....| ....|....| ....|....| ....|....| ....|....| ....|....| ....|....|**

**5010 5020 5030 5040 5050 5060 5070 5080 5090 5100**

**Morex**  AAACTGCAGC CATTCCCCTT CCAGGAGCAC ACCGACCTGC CAGTAGGCCC TACTGGTGGC ATGGGTGGGC AGGCTGAAGG CCCCACCTGT GGCATGGGTG

**Harrington**  AAACTGCAGC CATTCCCCTT CCAGGAGCAC ACCGACCTGC CAGTAGGCCC TACTGGTGGC ATGGGTGGGC AGGCTGAAGG CCCCACCTGT GGCATGGGTG

**HA52**  AAACTGCAGC CATTCCCCTT CCAGGAGCAC ACCGACCTGC CAGTAGGCCC TACTGGTGGC ATGGGTGGGC AGGCTGAAGG CCCCACCTGT GGCATGGGTG

**z043**  AAACTGCAGC CATTCCCCTT CCAGGAGCAC ACCGACCTGC CAGTAGGCCC TACTGGTGGC ATGGGTGGGC AGGCTGAAGG CCCCACCTGT GGCATGGGTG

**L47**  AAACTGCAGC CATTCCCCTT CCAGGAGCAC ACCGACCTGC CAGTAGGCCC TACTGGTGGC ATGGGTGGGC AGGCTGAAGG CCCCACCTGT GGCATGGGTG

**Strider**  AAACTACAGC CATTCCCCTT CCAGGAGCAC ACCGACCTGC CAATAGGCCC TACTGGTGGC ATGGGTGGGC AGGCTGAAGG CCCCACCTGT GGCATGGGTG

**W127**  AAACTGCAGC CATTCCCCTT CCAGGAGCAC ACCGACCTGC CAGTAGGCCC TACTGGTGGC ATGGGTGGGC AGGCTGAAGG CCCCACCTGT GGCATGGGTG

**Adorra**  AAACTGCAGC CATTCCCCTT CCAGGAGCAC ACCGACCTGC CAGTAGGCCC TACTGGTGGC ATGGGTGGGC AGGCTGAAGG CCCCACCTGT GGCATGGGTG

**Haruna Nijo**  AAACTGCAGC CATTCCCCTT CCAGGAGCAC ACCGACCTGC CAGTAGGCCC TACTGGTGGC ATGGGTGGGC AGGCTGAAGG CCCCACCTGT GGCATGGGTG

**L35**  AAACTGCAGC CATTCCCCTT CCAGGAGCAC ACCGACCTGC CAGTAGGCCC TACTGGTGGC ATGGGTGGGC AGGCTGAAGG CCCCACCTGT GGCATGGGTG

**L48**  AAACTACAGC CATTCCCCTT CCAGGAGCAC ACCGACCTGC CAATAGGCCC TACTGGTGGC ATGGGTGGGC AGGCTGAAGG CCCCACCTGT GGCATGGGTG

**L68**  AAACTGCAGC CATTCCCCTT CCAGGAGCAC ACCGACCTGC CAGTAGGCCC TACTGGTGGC ATGGGTGGGC AGGCTGAAGG CCCCACCTGT GGCATGGGTG

**PI296897**  AAACTGCAGC CATTCCCCTT CCAGGAGCAC ACCGACCTGC CAGTAGGCCC TACTGGTGGC ATGGGTGGGC AGGCTGAAGG CCCCACCTGT GGCATGGGTG

**AB75**  AAACTGCAGC CATTCCCCTT CCAGGAGCAC ACCGACCTGC CAGTAGGCCC TACTGGTGGC ATGGGTGGGC AGGCTGAAGG CCCCACCTGT GGCATGGGTG

**m279**  AAACTGCAGC CATTCCCCTT CCAGGAGCAC ACCGACCTGC CAGTAGGCCC TACTGGTGGC ATGGGTGGGC AGGCTGAAGG CCCCACCTGT GGCATGGGTG

**Legacy**  AAACTGCAGC CATTCCCCTT CCAGGAGCAC ACCGACCTGC CAGTAGGCCC TACTGGTGGC ATGGGTGGGC AGGCTGAAGG CCCCACCTGT GGCATGGGTG

**Orca**  AAACTGCAGC CATTCCCCTT CCAGGAGCAC ACCGACCTGC CAGTAGGCCC TACTGGTGGC ATGGGTGGGC AGGCTGAAGG CCCCACCTGT GGCATGGGTG

**Tango**  AAACTGCAGC CATTCCCCTT CCAGGAGCAC ACCGACCTGC CAGTAGGCCC TACTGGTGGC ATGGGTGGGC AGGCTGAAGG CCCCACCTGT GGCATGGGTG

**UC958**  AAACTGCAGC CATTCCCCTT CCAGGAGCAC ACCGACCTGC CAGTAGGCCC TACTGGTGGC ATGGGTGGGC AGGCTGAAGG CCCCACCTGT GGCATGGGTG

**UC960**  AAACTGCAGC CATTCCCCTT CCAGGAGCAC ACCGACCTGC CAGTAGGCCC TACTGGTGGC ATGGGTGGGC AGGCTGAAGG CCCCACCTGT GGCATGGGTG

**Steptoe**  AAACTGCAGC CATTCCCCTT CCAGGAGCAC ACCGACCTGC CAGTAGGCCC TACTGGTGGC ATGGGTGGGC AGGCTGAAGG CCCCACCTGT GGCATGGGTG

**Stander**  AAACTGCAGC CATTCCCCTT CCAGGAGCAC ACCGACCTGC CAGTAGGCCC TACTGGTGGC ATGGGTGGGC AGGCTGAAGG CCCCACCTGT GGCATGGGTG

**Ashqelon**  AAACTGCAGC CATTCCCCTT CCAGGAGCAC ACCGACCTGC CAGTAGGCCC TACTGGTGGC ATGGGTGGGC AGGCTGAAGG CCCCACCTGT GGCATGGGTG

**L46**  AAACTACAGC CATTCCCCTT CCAGGAGCAC ACCGACCTGC CAATAGGCCC TACTGGTGGC ATGGGTGGGC AGGCTGAAGG CCCCACCTGT GGCATGGGTG

**....|....| ....|....| ....|....| ....|....| ....|....| ....|....| ....|....| ....|....| ....|....| ....|.**

**5110 5120 5130 5140 5150 5160 5170 5180 5190**

**Morex**  GGCAAGTTAA AGGCCCTACT GGTGGCATGG GTGGGCAGGC TGAAGACCCT ACTAGTGGCA TGGGTGGGGA GCTCCCTGCC ACCATGTAAT GGAACC

**Harrington**  GGCAAGTTAA AGGCCCTACT GGTGGCATGG GTGGGCAGGC TGAAGACCCT ACTAGTGGCA TGGGTGGGGA GCTCCCTGCC ACCATGTAAT GGAACC

**HA52**  GGCAAGTTAA AGGCCCTACT GGTGGCATGG GTGGGCAGGC TGAAGACCCT ACTAGTGGCA TGGGTGGGGA GCTCCCTGCC ACCATGTAAT GGAACC

**z043**  GGCAAGTTAA AGGCCCTACT GGTGGCATGG GTGGGCAGGC TGAAGACCCT ACTAGTGGCA TGGGTGGGGA GCTCCCTGCC ACCATGTAAT GAATTC

**L47**  GGCAAGTTAA AGGCCCTACT GGTGGCATGG GTGGGCAGGC TGAAGACCCT ACTAGTGGCA TGGGTGGGGA GCTCCCTGCC ACCATGTAAT GAATT-

**Strider**  GGCAAGTTAA AGGCCCTACT GGTGGCATGG GTAGGCAGGC TGAAGACCCT ACTAGTGGCA TGGGTGGGGA GCTCCCTGCC ACCATGTAAT GGAACC

**W127**  GGCAAGTTAA AGGCCCTACT GGTGGCATGG GTGGGCAGGC TGAAGACCCT ACTAGTGGCA TGGGTGGGGA GCTCCCTGCC ACCATGTAAT G-----

**Adorra**  GGCAAGTTAA AGGCCCTACT GGTGGCATGG GTGGGCAGGC TGAAGACCCT ACTAGTGGCA TGGGTGGGGA GCTCCCTGCC ACCATGTAAT GGATCC

**Haruna Nijo**  GGCAAGTTAA AGGCCCTACT GGTGGCATGG GTGGGCAGGC TGAAGACCCT ACTAGTGGCA TGGGTGGGGA GCTCCCTGCC ACCATGTAAT GGAACC

**L35**  GGCAAGTTAA AGGCCCTACT GGTGGCATGG GTGGGCAGGC TGAAGACCCT ACTAGTGGCA TGGGTGGGGA GCTCCCTGCC ACCATGTAAT GAATTC

**L48**  GGCAAGTTAA AGGCCCTACT GGTGGCATGG GTAGGCAGGC TGAAGACCCT ACTAGTGGCA TGGGTGGGGA GCTCCCTGCC ACCATGTAAT GAATTC

**L68**  GGCAAGTTAA AGGCCCTACT GGTGGCATGG GTGGGCAGGC TGAAGACCCT ACTAGTGGCA TGGGTGGGGA GCTCCCTGCC ACCATGTAAT GAATT-

**PI296897**  GGCAAGTTAA AGGCCCTACT GGTGGCATGG GTGGGCAGGC TGAAGACCCT ACTAGTGGCA TGGGTGGGGA GCTCCCTGCC ACCATGTAAT GGATCC

**AB75**  GGCAAGTTAA AGGCCCTACT GGTGGCATGG GTGGGCAGGC TGAAGACCCT ACTAGTGGCA TGGGTGGGGA GCTCCCTGCC ACCATGTAAT GGAACC

**m279**  GGCAAGTTAA AGGCCCTACT GGTGGCATGG GTGGGCAGGC TGAAGACCCT ACTAGTGGCA TAGGTGGGGA GCTCCCTGCC ACCATGTAAT G-----

**Legacy**  GGCAAGTTAA AGGCCCTACT GGTGGCATGG GTGGGCAGGC TGAAGACCCT ACTAGTGGCA TGGGTGGGGA GCTCCCTGCC ACCATGTAAT GGAACC

**Orca**  GGCAAGTTAA AGGCCCTACT GGTGGCATGG GTGGGCAGGC TGAAGACCCT ACTAGTGGCA TGGGTGGGGA GCTCCCTGCC ACCATGTAAT GGAACC

**Tango**  GGCAAGTTAA AGGCCCTACT GGTGGCATGG GTGGGCAGGC TGAAGACCCT ACTAGTGGCA TGGGTGGGGA GCTCCCTGCC ACCATGTAAT GGAACC

**UC958**  GGCAAGTTAA AGGCCCTACT GGTGGCATGG GTGGGCAGGC TGAAGACCCT ACTAGTGGCA TGGGTGGGGA GCTCCCTGCC ACCATGTAAT GGAACC

**UC960**  GGCAAGTTAA AGGCCCTACT GGTGGCATGG GTGGGCAGGC TGAAGACCCT ACTAGTGGCA TGGGTGGGGA GCTCCCTGCC ACCATGTAAT GGAACC

**Steptoe**  GGCAAGTTAA AGGCCCTACT GGTGGCATGG GTGGGCAGGC TGAAGACCCT ACTAGTGGCA TGGGTGGGGA GCTCCCTGCC ACCATGTAAT GGAACC

**Stander**  GGCAAGTTAA AGGCCCTACT GGTGGCATGG GTGGGCAGGC TGAAGACCCT ACTAGTGGCA TGGGTGGGGA GCTCCCTGCC ACCATGTAAT GGAACC

**Ashqelon**  GGCAAGTTAA AGGCCCTACT GGTGGCATGG GTGGGCAGGC TGAAGACCCT ACTAGTGGCA TGGGTGGGGA GCTCCCTGCC ACCATGTAAT GGAACC

**L46**  GGCAAGTTAA AGGCCCTACT GGTGGCATGG GTAGGCAGGC TGAAGACCCT ACTAGTGGCA TGGGTGGGGA GCTCCCTGCC ACCATGTAAT GAATTC

The *Bmy1* haplotypes and GeneBank accessions numbers are as follows. *Sd1*: Morex (EU589328), Harrington (FJ161079), HA52 (AJ301645), Strider (EU589327) z043 (KF302668), L46 (L302672) and L47 (KF302673); *Sd2H* Haruna Nijo (D49999), PI296897 (AF061204), L35 (KF302671), L48 (KF302674) and L68 (KF302675); *Sd2L*: Adorra (AF061203), Hiproly (X52321) and m279 (KF302670); *Sd3:* AB75; *Sd4*: Steptoe (EF175470.1), Orca (EF175468), Legacy (FJ161080), Stander (EF175469), Tango (EF175471), UC958 (EF175472.1), UC960 (EF175473.1), Schooner (AF300799), Franklin (AF300800); *Sd5*: Ashqelon (FJ161078) and W127 (KF302669)

The unique nucleotide polymorphism identified in this study was marked in rectangles. The exons of the *Bmy1* gene were underlined with sold line
