# Supplementary material for: Discovery of Novel Bmy1 Alleles Increasing β-Amylase Activity in Chinese Landraces and Tibetan Wild Barley for Improvement of Malting Quality via MAS
Source: PLoS One. 2013 Sep 3;8(9):e72875. doi: 10.1371/journal.pone.0072875 (PMC3760831; doi:10.1371/journal.pone.0072875)
Supplement: Table S5 — INDELs positions and unique SNPs identified in Bmy1 compared to 7 Bmy1 haplotypes. (DOC) [file pone.0072875.s005.doc]

**Table S5**. INDELs (insertion/deletion) position and unique SNPs identified in *Bmy1* compared to 7 *Bmy1* haplotypes.

| Position  bp | SNP/INDELS | Sd1a | | | | Sd1b | Sd1c | Sd2L | | Sd2H | | | | Sd2Ha | Sd3 | Sd4 | Sd5 | |
| --- | --- | --- | --- | --- | --- | --- | --- | --- | --- | --- | --- | --- | --- | --- | --- | --- | --- | --- |
|  |  | Harrington | HA52 | z043 | L47 | Strider | L46 | Adorra | m279 | Haruna  Nijo | L35 | L48 | L68 | PI  296897 | AB75 | legacy | Ashquelon | W127 |
| 111 | G → T | - | - | + | - | NA | + | - | + | NA | - | - | - | - | - | - | - | - |
| 148 | 1 bp | - | + | - | - | NA | - | - | - | NA | - | - | - | - | - | - | - | - |
| 177 | 1 bp | + | - | - | + | NA | - | + | - | NA | - | - | - | - | - | - | - | - |
| 205 | 11 bp | + | + | - | + | NA | - | + | - | NA | + | + | + | + | + | + | + | + |
| 266 | 1 bp | + | + | - | + | NA | - | + | - | NA | + | + | + | + | + | - | + | + |
| 275 | 3 bp | - | - | - | - | NA | - | + | - | NA | - | - | - | - | + | - | - | - |
| 341 | 1 bp | + | - | + | + | NA | + | + | + | NA | + | + | + | + | + | + | + | + |
| 343 | 2 BP | + | - | + | + | NA | + | + | + | NA | + | + | + | + | + | + | + | + |
| 400 | 1 bp | + | + | + | + | NA | - | + | - | NA | + | + | + | - | + | + | + | + |
| 411 | 4 bp | + | + | + | + | NA | - | + | + | NA | - | + | - | - | - | - | - | - |
| 428 | 8 bp | - | - | - | - | NA | + | - | + | NA | + | + | + | + | + | + | + | + |
| 491 | 11 bp | + | + | + | + | NA | + | + | + | NA | - | - | - | + | - | + | - | - |
| 505 | 8 bp | + | + | + | + | NA | + | + | + | NA | - | - | - | + | - | + | - | - |
| 523 | A | - | - | + | + | NA | + | + | + | NA | + | + | + | + | + | + | + | + |
| 558 | T → C | - | + | - | - | NA | + | - | - | NA | - | - | - | - | - | - | - | - |
| 595 | 1 bp | - | + | - | - | NA | - | - | - | NA | - | - | + | - | - | - | - | - |
| 605 | 1 bp | - | + | - | - | NA | - | - | - | NA | - | - | - | - | - | - | - | - |
| 675 | 1 bp | - | - | - | - | NA | - | - | - | NA | - | - | - | - | - | + | - | - |
| 700 | 1 bp | + | + | + | + | NA | + | - | - | NA | - | - | - | - | - | - | - | - |
| 714 | C → G | - | - | - | - | NA | - | - | - | NA | + | + | + | - | - | - | - | + |
| 734 | 92 bp | - | - | + | - | NA | + | + | + | NA | + | + | + | + | + | + | + | + |
| 765 | T → C | - | - | - |  | NA | + | - | + | NA | + | + | + | - | - | - | - | + |
| 801 | T → C | - | - | + | - | NA | - | - | - | NA | - | - | - | - | - | - | - | - |
| 830 | A → G | - | - | - | - | NA | + | - | - | NA | - | - | - | - | - | - | - | - |
| 967 | 1 bp | + | + | + | + | NA | + | - | - | NA | - | - | - | - | - | + | - | - |
| 995 | T → G | - | - | - | + | - | - | - | + | - | + | + | + | - | - | - | - | + |
|  |  | Harrington | HA52 | z043 | L47 | Strider | L46 | Adorra | m279 | Haruna  Nijo | L35 | L48 | L68 | PI  296897 | AB75 | legacy | Ashquelon | W127 |
| 1085 | T → C | - | - | - | - | NA | - | - | + | NA | + | + | + | - | - | - | - | + |
| 1097 | C → T | - | - | - | - | NA | - | - | + | NA | + | + | + | - | - | - | - | + |
| 1203 | 1 bp | - | - | - | - | NA | - | + | - | NA | - | - | - | - | - | - | - | - |
| 1216 | 1 bp | - | + | - | - | NA | - | - | - | NA | - | - | - | - | - | - | - | - |
| 1300 | T → A | - | - | + | - | NA | - | - | - | NA | - | - | - | - | - | - | - | - |
| 1426 | T → C | - | - | - | - | - | - | - | - | - | + | - | - | - | - | - | - | - |
| 1621 | 4 bp | - | - | - | - | + | - | + | + | + | + | + | + | + | + | + | - | - |
| 2063 | 14 bp | + | + | + | + | + | - | - | - | - | - | - | - | - | - | - | - | + |
| 2123 | 1 bp | + | + | + | + | + | - | - | - | - | - | - | - | - | - | - | - | + |
| 2207 | T → C | - | - | - | - | - | - | - | - | - | + | + | + | - | - | - | - | - |
| 2314 | 1 bp | - | - | - | - | - | - | - | - | - | - | - | - | - | + | - | - | - |
| 2364 | 1 bp | - | - | - | - | - | - | - | - | - | - | - | - | - | + | - | - | - |
| 2635 | 126 bp | - | - | - | - | - | - | + | + | - | - | - | - | - | - | + | - | - |
| 2797 | G → C | - | - | - | - | - | - | - | - | - | + | - | + | - | - | - | - | - |
| 2831 | 1 bp | + | + | + | + | + | + | + | + | + | + | + | + | + | + | - | + | + |
| 2839 | 38 bp | + | + | + | - | + | + | + | + | + | + | + | + | - | + | + | + | + |
| 2939 | T → C | - | - | - | - | - | - | - | - | - | - | + | - | - | - | - | - | - |
| 2954 | 1 bp | + | + | + | - | + | + | + | + | + | + | + | + | + | - | + | + | + |
| 3023 | 1 bp | - | - | - | + | - | + | - | - | + | + | + | + | + | + | - | + | + |
| 3079 | 2 bp | + | + | + | + | + | + | - | - | + | + | + | + | + | + | - | + | + |
| 3086 | 1 bp | + | - | + | + | + | + | + | + | + | + | + | + | + | + | + | + | + |
| 3124 | A → G | - | - | - | - | - | - | - | - | - | - | - | + | - | - | - | - | - |
| 3209 | 11 bp | - | - | - | + | - | + | - | - | + | + | + | + | + | + | - | + | + |
| 3301 | 4 bp | + | + | + | - | + | - | + | + | - | - | - | - | - | - | + | - | - |
| 3337 | 21 bp | - | - | - | + | - | + | + | + | + | + | + | + | + | + | + | + | + |
| 3399 | 1 bp | - | - | - | - | - | - | + | - | - | - | - | - | - | - | - | - | - |
| 3470 | 1 bp | - | - | - | - | - | - | + | + | - | - | - | - | - | - | - | - | - |
| 3529 | 1 bp | - | - | - | + | - | + | + | + | + | + | - | + | + | + | + | + | + |
|  |  | Harrington | HA52 | z043 | L47 | Strider | L46 | Adorra | m279 | Haruna  Nijo | L35 | L48 | L68 | PI  296897 | AB75 | legacy | Ashquelon | W127 |
| 3532 | 1 bp | + | + | + | + | + | + | + | + | + | + | + | + | + | + | - | + | + |
| 3602 | 1 bp | + | + | + | + | + | + | + | + | + | + | + | + | + | + | + | - | - |
| 3634 | 1 bp | + | + | + | - | + | - | + | + | - | - | + | - | - | - | + | - | - |
| 3645 | 6 bp | + | + | + | + | + | - | + | + | - | - | + | - | - | - | + | - | - |
| 3653 | A → T | - | - | - | - | - | - | - | - | - | - | + | - | - | - | - | - | - |
| 3658 | T → G | - | - | - | + | - | - | - | - | - | - | + | - | - | - | - | - | - |
| 3663 | C → A | - | - | - | + | - | - | - | - | - | - | + | - | - | - | - | - | - |
| 4104 | 6 bp | - | - | - | - | - | + | - | - | - | - | + | - | - | - | + | - | - |
| 4135 | 1 bp | - | + | - | - | - | - | - | - | - | - | - | - | - | - | - | - | - |
| 4389 | T → C | - | - | + | - | - | - | - | - | - | - | - | - | - | - | - | - | - |
| 4496 | 10 | + | + | + | + | + | + | + | + | + | + | + | + | + | - | + | + | + |
| 4502 | 1 bp | - | + | - | + | - | - | - | - | - | - | - | - | - | - | - | - | - |
| 4507 | 1 bp | + | + | + | + | - | - | - | - | - | - | - | - | - | - | - | - | + |
| 4627 | A → G | - | - | - | - | - | - | - | - | - | - | - | - | - | - | - | - | + |
| 4697 | A → G | - | - | - | + | - | - | - | - | - | - | - | - | - | - | - | - | - |
| 4907 | T → C | - | - | - | - | - | - | - | - | - | - | + | + | - | - | - | - | - |
