# Supplementary material for: Discovery of Novel Bmy1 Alleles Increasing β-Amylase Activity in Chinese Landraces and Tibetan Wild Barley for Improvement of Malting Quality via MAS
Source: PLoS One. 2013 Sep 3;8(9):e72875. doi: 10.1371/journal.pone.0072875 (PMC3760831; doi:10.1371/journal.pone.0072875)
Supplement: Table S6 — The position, size and sequence of Bmy1 genomic INDELs (insertion/deletion). (DOC) [file pone.0072875.s006.doc]

**Table S6**. The position, size and sequence of *Bmy1* genomic wide INDELs (insertion/deletion).

| Position | Size  bp | Sequence | Position | Size  bp | Sequence |
| --- | --- | --- | --- | --- | --- |
| 148 | 1 | A | 2364 | 1 | T |
| 177 | 1 | A | 2635 | 126 | CTCCCTTCGTCCTAAAATTCTTGTCTTAGCTTTGTCTAGAAATTGATGTATC  TAAATACTAAAACTTAACTAGATACATTCATATCTAAACAAATCTAAGACA  AGAATTTTGGAACGGAGGTAGTA |
| 205 | 11 | TGAGAAGTGAA | 2831 | 1 | T |
| 266 | 1 | G | 2839 | 38 | CAAACACCCAAATGAAAAGTGATTCTTAAAGGAAAAAA |
| 275 | 3 | GAA | 2954 | 1 | T |
| 341 | 1 | T | 3023 | 1 | A |
| 342 | 2 | TT | 3079 | 2 | CA / T |
| 400 | 1 | C | 3086 | 1 | T |
| 411 | 4 | TCTA | 3125 | 1 | C |
| 428 | 8 | CAAATATT | 3143 | 1 | C |
| 491 | 11 | GCGAAAAGGAG | 3209 | 11 | TTGAAAGTAGG |
| 505 | 8 | CGGTTCGG | 3306 | 4 | GCAC |
| 523 | 1 | A | 3337 | 21 | AGAAAGGTGGGCTATGCATTT |
| 527 | 1 | A | 3399 | 1 | A |
| 595 | 1 | T | 3470 | 1 | A |
| 605 | 1 | T | 3529 | 1 | C |
| 675 | 1 | T | 3532 | 1 | T |
| 700 | 1 | G | 3602 | 1 | C |
| 734 | 92 | CGGTCATGATGTGGCTTGGATCCCAAGTTAGCTATACAGATAAGG  ATATATCTTACCTCAACCGAATCTAGGTTACAACAA GCTTAACATTC | 3634 | 1 | A |
| 967 | 1 | G | 3645 | 6 | CGTACT |
| 1203 | 1 | T | 4104 | 6 | CGCATT |
| 1216 | 1 | G | 4135 | 1 | G |
| 1236 | 1 | C | 4427 | 1 | A |
| 1419 | 1 | C | 4457 | 2 | T or TT |
| 1621 | 4 | AATA | 4496 | 10 | ATGCATACGG |
| 2063 | 14 | ATAGATATACATAT | 4502 | 1 | T |
| 2314 | 1 | T | 4507 | 1 | G |
